# Palladium(ii) complexes bearing mesoionic carbene ligands: catalytic application in domino Sonogashira coupling/cyclization reactions for one-pot synthesis of benzofuran and indole derivatives†

**DOI:** 10.1039/d4ra03485f

**Published:** 2024-08-27

**Authors:** Om Prakash Joshi, Ramalingam Thirumoorthi, Ram T. Pardasani, Sriparna Ray, Chandrakanta Dash

**Affiliations:** a Department of Chemistry, School of Chemical Sciences and Pharmacy, Central University of Rajasthan Bandarsindri Ajmer 305817 Rajasthan India ckdash@curaj.ac.in; b Catalytic Applications Laboratory, Department of Chemistry, School of Basic Sciences, Faculty of Science, Manipal University Jaipur Dehmi Kalan Jaipur 303007 Rajasthan India sriparna.ray@gmail.com

## Abstract

Bioactive heterocycles such as benzofuran and indole derivatives were synthesized from commercially available 2-iodoarenes and alkynes *via* domino Sonogashira coupling followed by cyclization reaction using well-defined palladium PEPPSI (Pyridine Enhanced Precatalyst Preparation Stabilization and Initiation) complexes (2a and 2b). These reactions tolerate a variety of 2-iodoarenes and diversely substituted terminal alkynes, resulting in the corresponding product in moderate to good yields in an open-air atmosphere. In particular, two palladium(ii) PEPPSI complexes 2a and 2b were synthesized in good yields from the reaction of corresponding 1,2,3-triazol-5-ylidene (MIC: mesoionic carbene), PdCl_2_, KI, and K_2_CO_3_ in pyridine at 110 °C and structurally characterized by various spectroscopic techniques including NMR spectroscopy, IR spectroscopy, HRMS and elemental analysis studies. Complex 2b is also characterized by X-ray crystallography.

## Abstract 2

Indole and benzofuran derivatives were synthesized with good diversity in moderate to good yields *via* a domino Sonogashira coupling/cyclization process using 1,2,3-triazolylidene-based palladium PEPPSI complexes.

## Graphical abstract

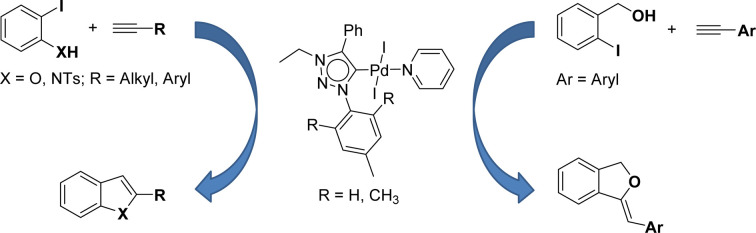

## Introduction

Benzofused five-membered heterocyclic scaffolds such as benzofuran and indole derivatives are extensively spread as a common structural skeleton in many biologically active compounds, natural products, and pharmaceuticals.^1–6^ Over the years, considerable effort has been devoted to synthesizing these heterocycles.^6–10^ Synthesis of benzofuran and indole derivatives *via* a domino approach has received more attention than the traditional multistep approach due to its high atom economy, environmentally benign nature, and operational simplicity.^1,8,11–15^ Along these lines, palladium-catalyzed domino Sonogashira coupling/cyclization reaction has emerged as an efficient method for the construction of the benzofuran and indole framework.^16–19^

N-heterocyclic carbenes (NHCs) have been used as spectator ligands in organometallic chemistry for the past three decades.^20–23^ Their strong metal-NHC bond-forming tendency and high steric demands have made them much more popular and a good alternative to conventional phosphine ligands.^20,24^ An interesting counterpart of normal NHCs, 1,2,3-triazol-5-ylidene mesoionic carbene (1,2,3-triazolylidene) was first reported by Albrecht and co-workers in 2008.^25^ In these MICs, only one adjacent electronegative nitrogen atom at the donor site increased the sigma donor ability compared to NHCs.^25^ Triazolylidenes can be easily synthesized *via* copper-catalyzed Click reaction followed by an alkylation reaction.^26–28^ Several triazolylidene-based transition metal complexes are reported as catalysts in various organic transformations.^27,29–38^ PEPPSI (Pyridine Enhanced Precatalyst Preparation Stabilization and Initiation) themed^39^ palladium complexes bearing 1,2,3-triazolylidene were reported by Albrecht *et al.* in 2012.^40^

**Fig. 1:**
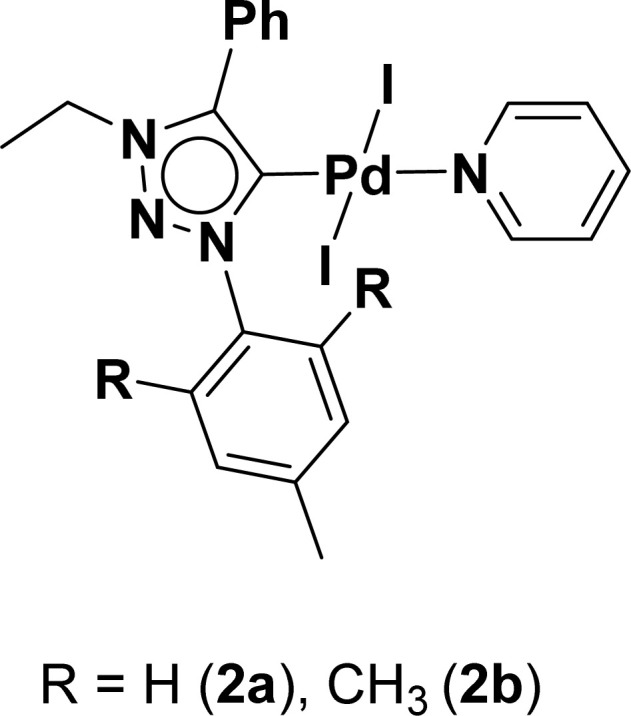
1,2,3-Triazol-5-ylidene based palladium PEPPSI complexes.

Very few well-defined palladium complexes bearing N-heterocyclic carbene ligands are used as catalysts in domino Sonogashira coupling/cyclization reactions for one-pot synthesis of benzofuran and indole derivatives.^41–43^ Mata and coworkers reported the triazole based N-heterocyclic carbene–palladium complex as catalyst for the synthesis of benzofuran derivatives.^41^ Ghosh and coworkers revealed the dipalladium complex stabilized by N-heterocyclic carbene ligand for benzofuran synthesis.^42^ Our group also reported various palladium complexes as catalysts for the synthesis of benzofurans *via* domino Sonogashira coupling and cyclization reactions.^43,44^ Bera and coworkers developed the annellated mesoionic carbene ligand stabilized palladium complexes and explored their catalytic activity for the synthesis of benzofuran, indole, isocoumarin, and isoquinolone derivatives using domino approach.^7^ Herein, we reported the synthesis of 1,2,3-triazol-5-ylidene mesoionic carbene-based palladium PEPPSI complexes, 2a and 2b (Fig. 1) and further utilized these catalysts for the synthesis of biologically active benzofuran and indole derivatives *via* domino Sonogashira coupling/cyclization reaction under copper and amine free condition.

## Results and discussion

### Synthesis and characterization of palladium PEPPSI complexes

**Scheme 1:**
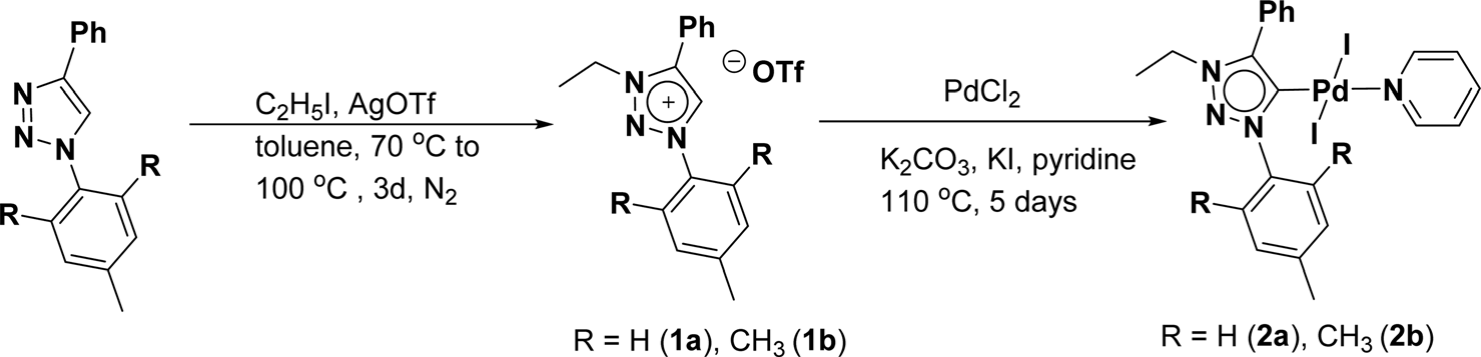
Synthesis of palladium-PEPPSI complex 2a and 2b.

The 1,2,3-triazolium triflate ligands 1a and 1b were synthesized by AgOTf-assisted *N*-ethylation of corresponding 1,2,3-triazole^40,45^ using ethyl iodide as ethylating reagent (Scheme 1). Formation of triazolium ligands was indicated by the appearance of a quartet peak of two protons which corresponds to triazolium methylene (trz–CH_2_–) peak in ^1^H NMR spectrum at 4.64 ppm (1a) and 4.76 ppm (1b), respectively. The 1,2,3-triazolylidene based palladium(ii) complexes (2a and 2b) were synthesized with good yield (2a = 74%, 2b = 83%) from the reaction of corresponding triazolium triflate, PdCl_2_, K_2_CO_3,_ and KI in pyridine at 110 °C in 5 days using a similar method reported earlier.^35,46^ The disappearance of a singlet of triazolium proton and the presence of a set of additional pyridine peaks in the aromatic region suggest the formation of palladium PEPPSI complexes. The ^13^C{^1^H} NMR spectrum of these complexes revealed Pd–C_carbene_ peak at around 134.4 ppm for 2a and 135.6 ppm for 2b, respectively. After purification, both the complexes were obtained as pale-yellow solid.

### X-ray structure

**Fig. 2:**
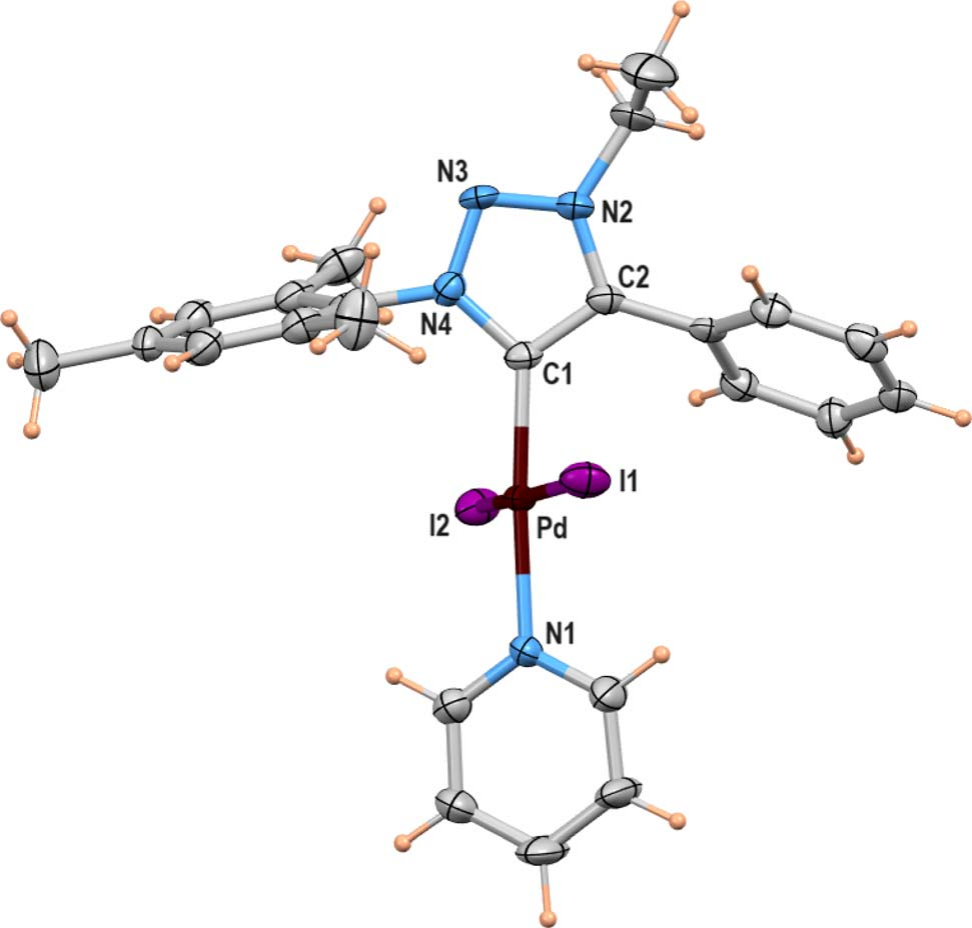
Molecular structure showing [LPdI_2_(pyridine)] (2b), ellipsoids are shown at 50% probability level. Only one of the molecules (out of two) present in the asymmetric unit is shown. Selected bond lengths (Å) and angles (deg): Pd(1)–N(1) 2.116(5), Pd(1)–C(1) 1.970(5), Pd(1)–I(1) 2.6208(6), Pd(1)–I(2) 2.5973(6), N(1)–Pd(1)–I(1) 92.12(14), N(1)–Pd(1)–I(2) 90.49(14), C(1)–Pd(1)–I(1) 87.90(17), C(1)–Pd(1)–I(2) 88.99(17); Pd(2)–N(5) 2.107(5), Pd(2)–C(25) 1.980(6), Pd(2)–I(3) 2.5801(6), Pd(2)–I(4) 2.6281(6), N(5)–Pd(2)–I(3) 90.93(14), N(5)–Pd(2)–I(4) 88.96(14), C(25)–Pd(2)–I(3) 89.24(16), C(25)–Pd(2)–I(4) 90.91(16).

A suitable single crystal for X-ray diffraction analysis was obtained for complex 2b by slow evaporation of solvent from a saturated solution of complex 2b in acetonitrile at room temperature. The formation of palladium PEPPSI complex 2b was confirmed by molecular structure analysis, and the geometry of the central palladium metal is found to be slightly distorted square planar in which 1,2,3-triazolylidene ligand located trans to pyridine and remaining coordination sites are occupied by iodido donors (Fig. 2). The average Pd–C_carbene_ and Pd–N_pyridine_ bond distances are 1.975(5) and 2.111(5), respectively, which are similar to analogous 1,2,3-trizolylidene ligand-based PEPPSI complexes previously reported in the literature.^47–49^ The average Pd–I bond lengths were also observed as close to the other reported PEPPSI type complexes containing iodide ligands.^49^ The average C_carbene_–Pd–N_pyridine_ and I–Pd–I bond angles are 174.4 (2)° and 175.96(2)°, respectively, which are closer to the expected 180° for a square planar geometry.

### Domino Sonogashira coupling/cyclization reactions

Based on our previous study,^43,44^ 2-iodophenol and phenylacetylene were chosen as model substrates for domino Sonogashira coupling/cyclization reactions. In the optimization experiments, the initial reaction condition included 2-iodophenol (0.50 mmol), phenylacetylene (0.60 mmol), K_2_CO_3_ (1 mmol), and catalyst 2a (2 mol%) in DMSO at 110 °C in air. Under this reaction condition, the desired benzofuran was obtained in 81% yield (entry 1, Table 1). Various other bases (entries 2–4, Table 1) and solvents (entries 5 and 6, Table 1) were screened for this reaction, with the result that K_3_PO_4_ base and DMSO solvent were found to be optimum for this reaction. Reducing the reaction time reduced the product yield to 65% (entry 7, Table 1). There was a negligible decrease in the product yield (83%) at 90 °C (entry 8, Table 1), but it decreased significantly to 41% at 60 °C (entry 9, Table 1). Reducing the catalyst loading to 1 mol% significantly decreases the product yield to 63% (entry 10, Table 1). The reaction with palladium precursor, *i.e.*, Pd(CH_3_CN)_2_Cl_2_ gave a yield of 36% (entry 11, Table 1). In continuation, poor yield of the product was obtained under base-free conditions (entry 12, Table 1), while no reaction was observed in absence of a palladium catalyst (entry 13, Table 1).

**Table 1:** Optimization of the reaction conditions for the synthesis of 2-arylbenzofuran^a^

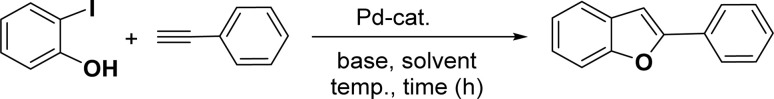						
Entry	Catalyst (mol%)	Base	Solvent	Time (h)	Temp. (°C)	% Yield^b^
1	2a (2.0)	K_2_CO_3_	DMSO	10	110	81
2	2a (2.0)	K_3_PO_4_	DMSO	10	110	84
3	2a (2.0)	KO^*t*^Bu	DMSO	10	110	51
4	2a (2.0)	KOH	DMSO	10	110	42
5	2a (2.0)	K_3_PO_4_	Dioxane	10	110	71
6	2a (2.0)	K_3_PO_4_	Toluene	10	110	37
7	2a (2.0)	K_3_PO_4_	DMSO	4	110	65
8	2a (2.0)	K_3_PO_4_	DMSO	10	90	83
9	2a (2.0)	K_3_PO_4_	DMSO	10	60	41
10	2a (1.0)	K_3_PO_4_	DMSO	10	90	63
11	Pd(CH_3_CN)_2_Cl_2_ (2.0)	K_3_PO_4_	DMSO	10	90	36
12	2a (2.0)	—	DMSO	10	90	10^c^
13	—	K_3_PO_4_	DMSO	10	90	nr^d^

a Reaction conditions: 2-iodophenol (0.50 mmol), phenylacetylene (0.60 mmol), 2.0 mol% of catalyst, base (1.00 mmol), and solvent (2 mL).

b Isolated yield.

c Without base.

d Without catalyst. nr = no reaction.

With the optimized reaction condition in hand (entry 8), various substituted benzofuran and indole derivatives were synthesized in moderate to good yields from commercially available terminal alkynes and 2-iodoarenes (Table 2). In comparison, benzofuran derivatives were obtained a higher yield (entries 1 to 7, Table 2) with respect to indole derivatives (entries 8 to 14, Table 2). Furthermore, there is not much difference in the catalytic activity of the catalysts 2a and 2b under the same reaction conditions was observed.

**Table 2:** Synthesis of benzofuran and indole derivatives *via* domino Sonogashira coupling/cyclization reactions catalyzed by 2a/2b^a^

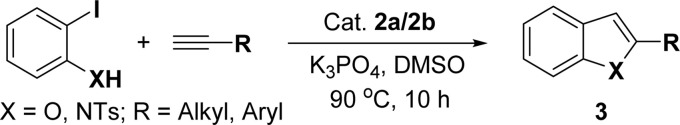					
Entry	2-Iodoarenes	Alkynes	Product	Yield^b^ (%)	
				2a	2b
1	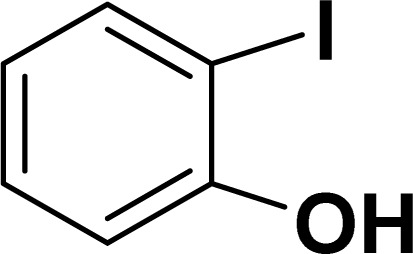	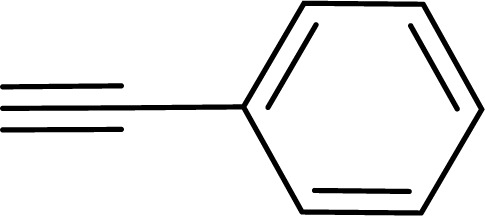	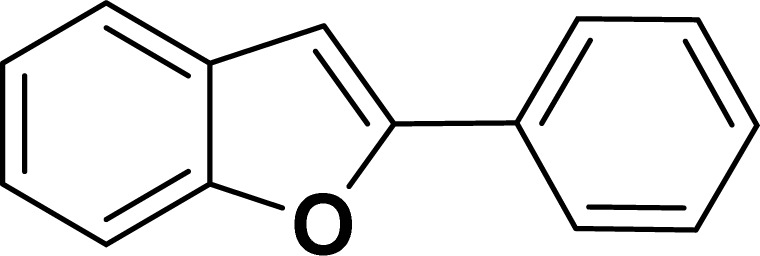	83	87
2	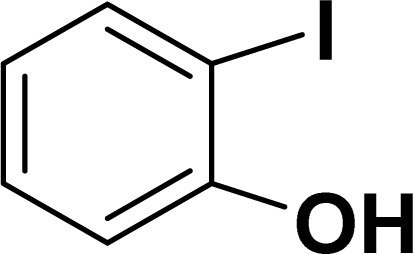	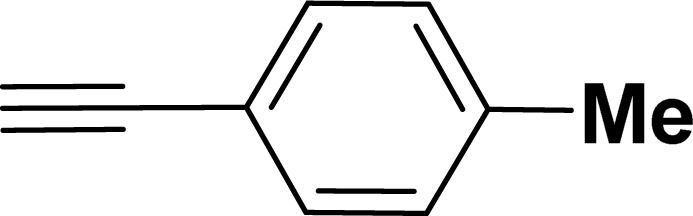	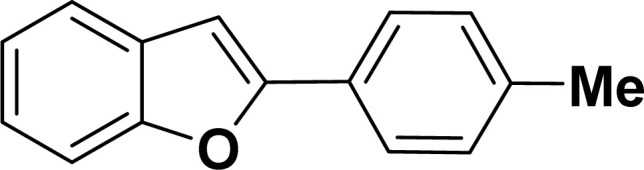	80	85
3	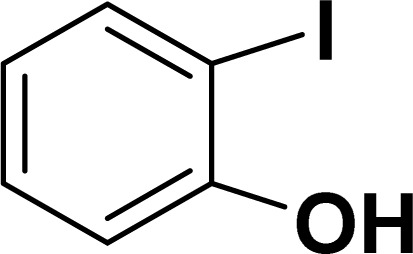	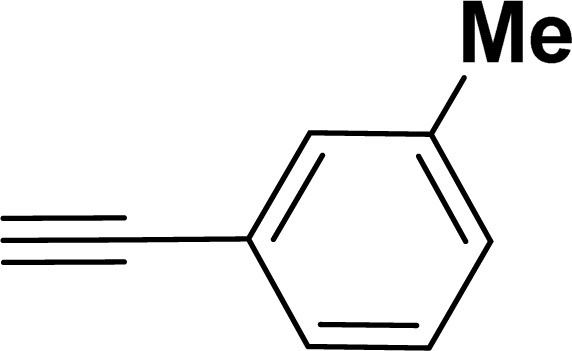	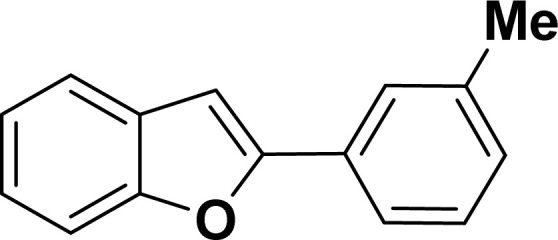	68	71
4	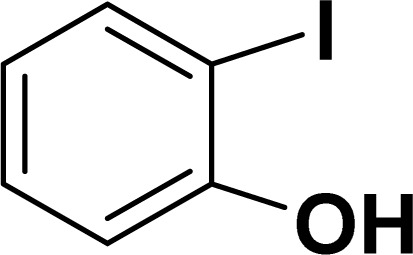	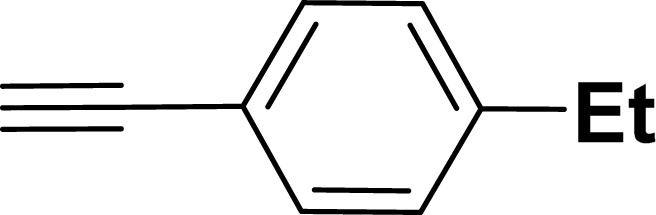	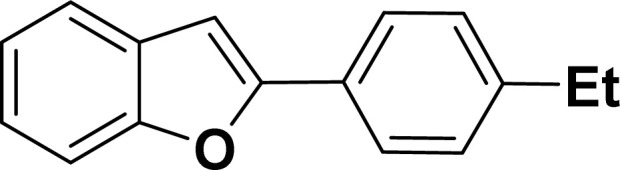	76	83
5	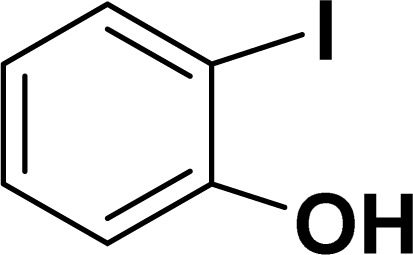	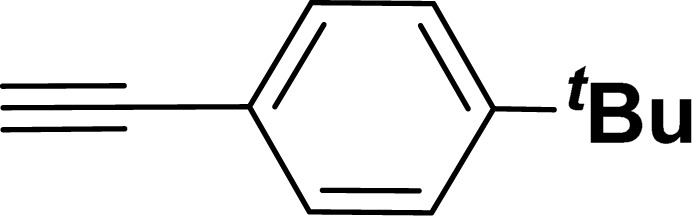	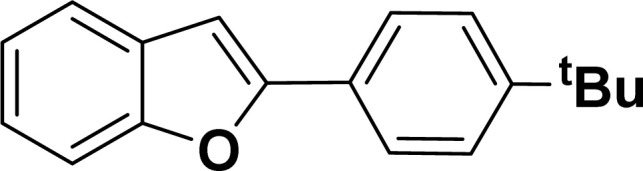	71	77
6	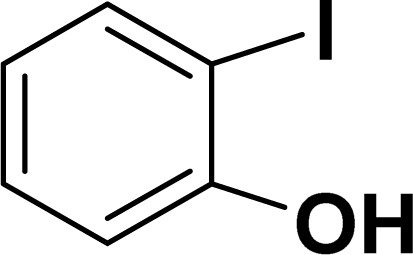	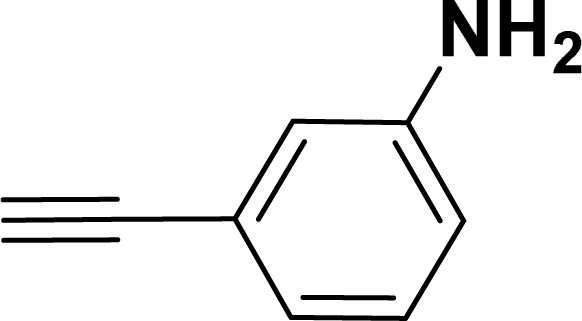	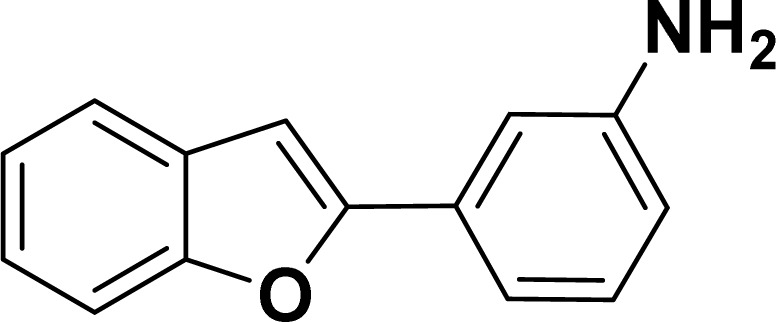	64	65
7	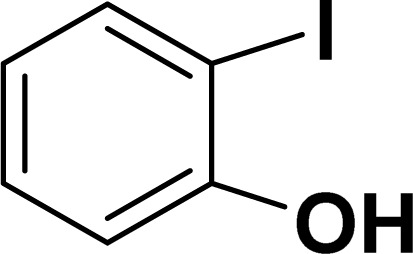	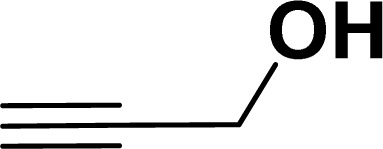	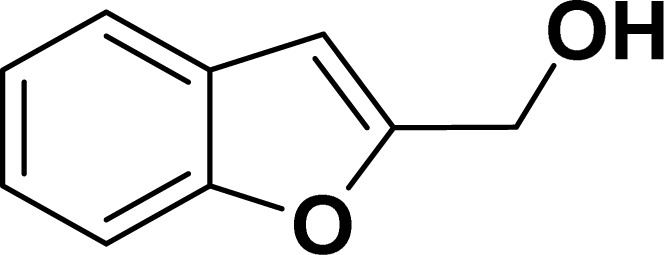	63	65
8	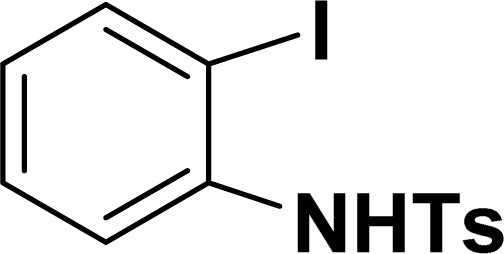	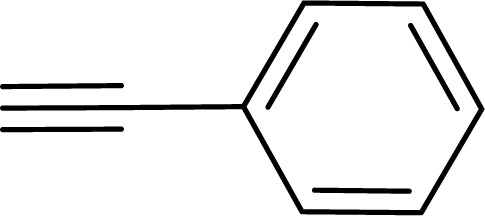	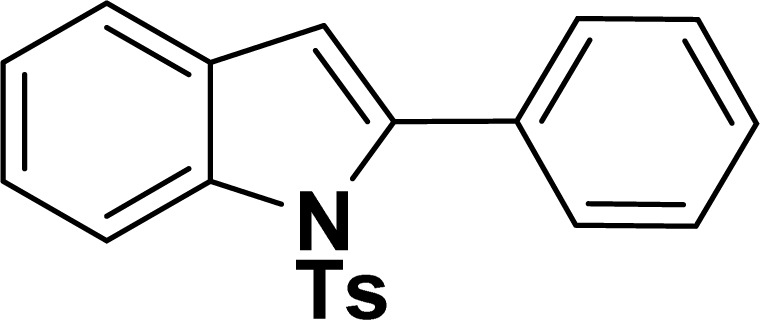	65	57
9	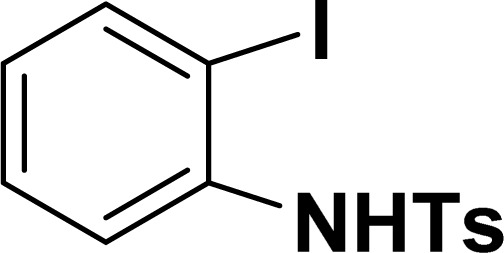	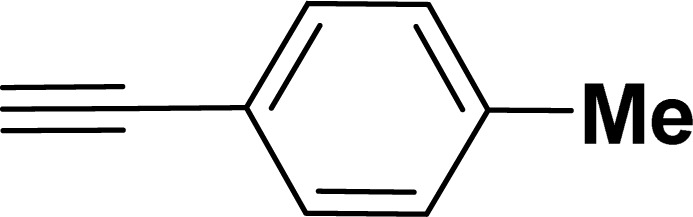	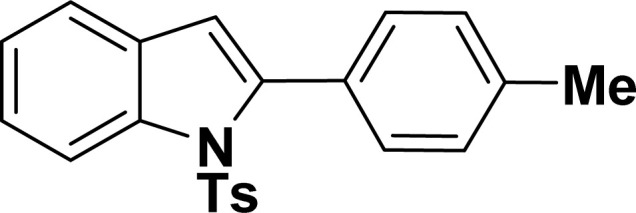	48	53
10	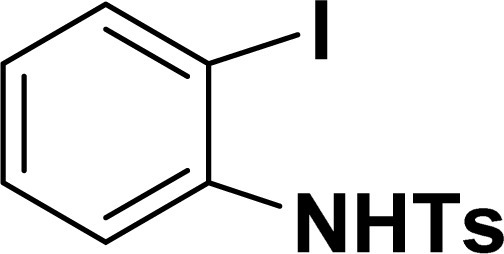	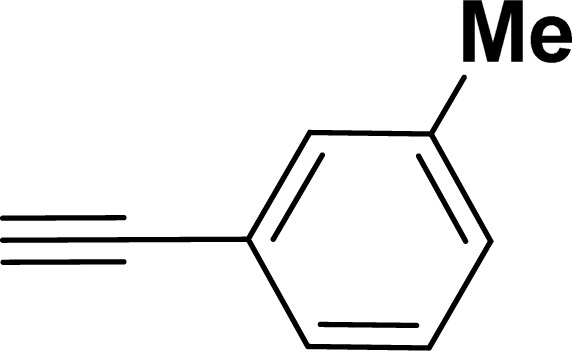	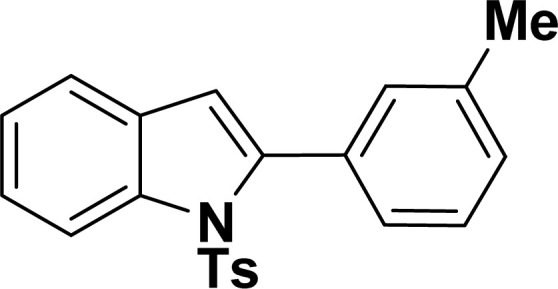	35	39
11	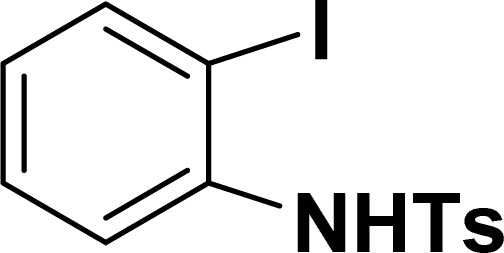	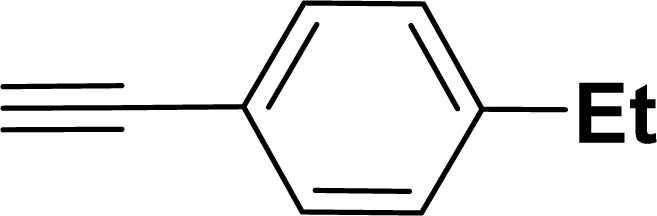	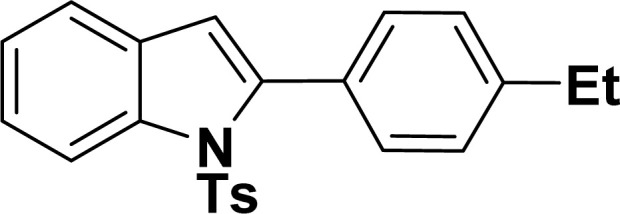	46	49
12	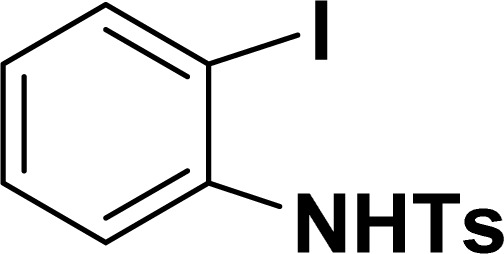	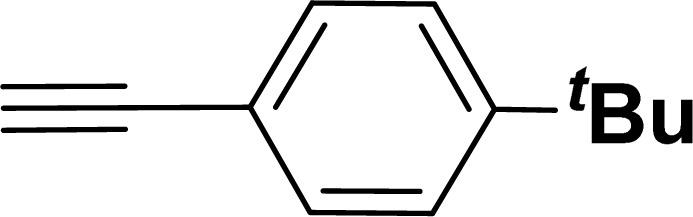	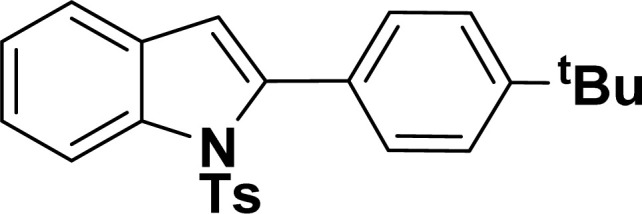	40	38
13	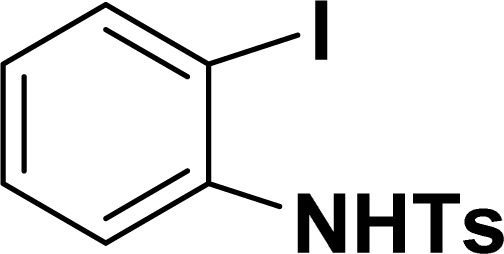	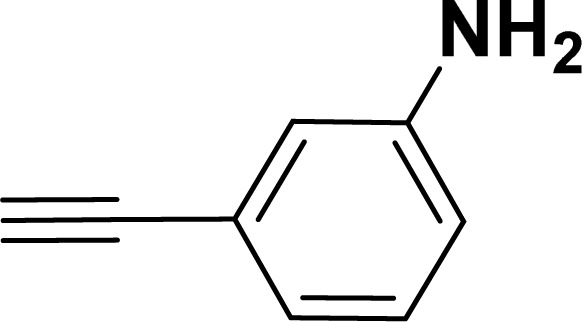	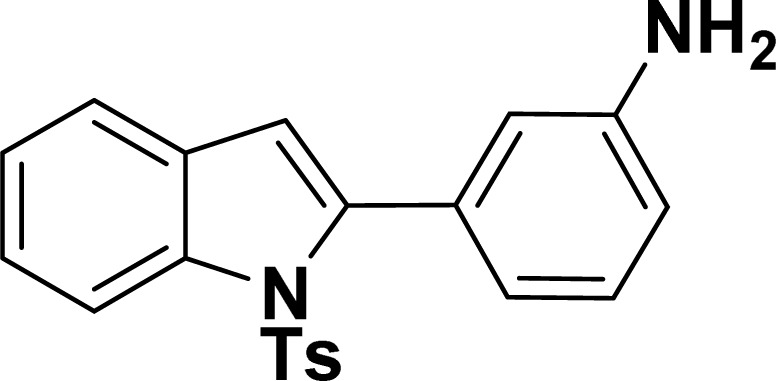	34	38
14	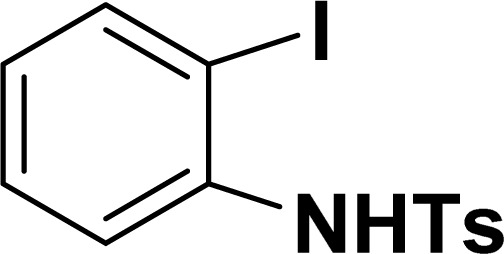	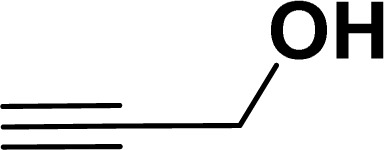	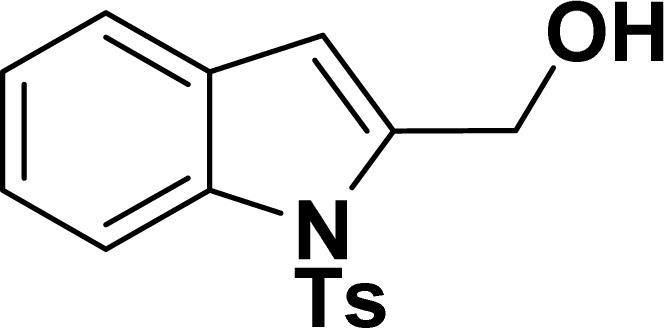	39	37

a Reaction conditions: 2-iodoarene derivative (0.50 mmol), alkyne (0.60 mmol), 2.0 mol% of 2a or 2b, K_3_PO_4_ (1.00 mmol), DMSO (2 mL), 90 °C, 10 h.

b Isolated yield.

Under the same reaction condition, (*Z*)-1-benzylidene-1,3-dihydroisobenzofuran derivatives were also synthesized in good yields from 2-iodobenzyl alcohols and terminal alkynes (Table 3). Formation of (*Z*)-1-benzylidene-1,3-dihydroisobenzofuran was confirmed from the presence of a singlet of one proton at *δ* 5.98 ppm for methine proton and singlet of two protons at *δ* 5.54 ppm for methylene protons, which matches with the previously reported NMR data.^50^

**Table 3:** Synthesis of isobenzofuran derivatives *via* domino Sonogashira coupling/cyclization reactions catalyzed by 2a/2b^a^

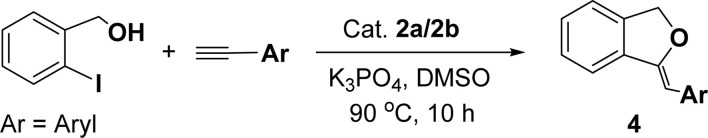					
Entry	Iodobenzyl alcohol	Alkyne	Product	Yield^b^ (%)	
				2a	2b
1	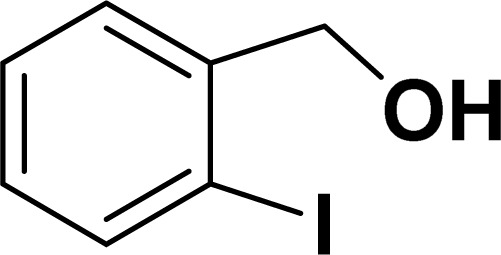	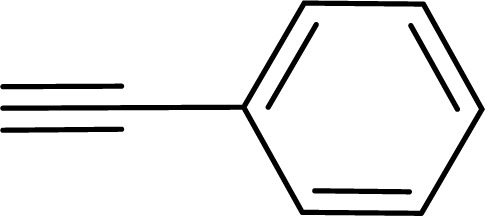	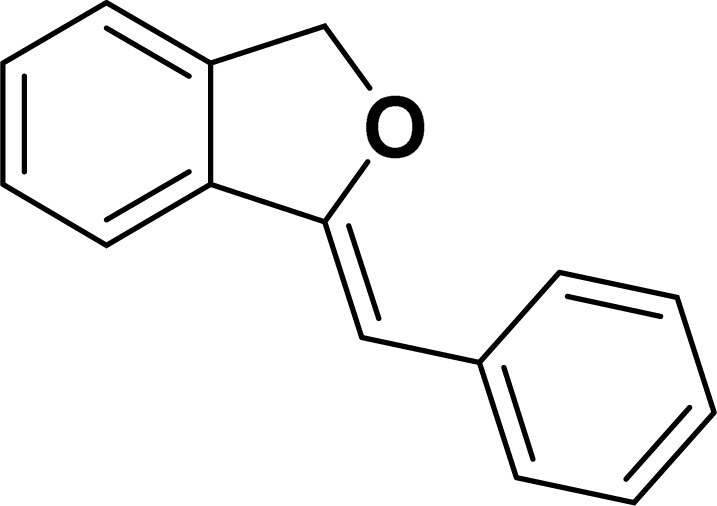	87	86
2	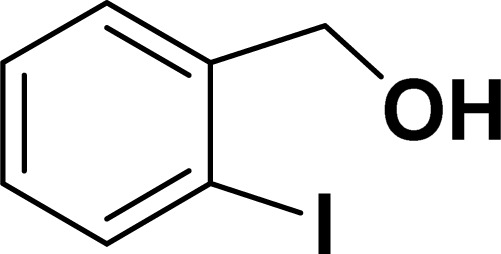	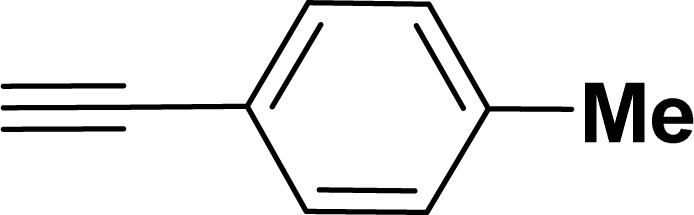	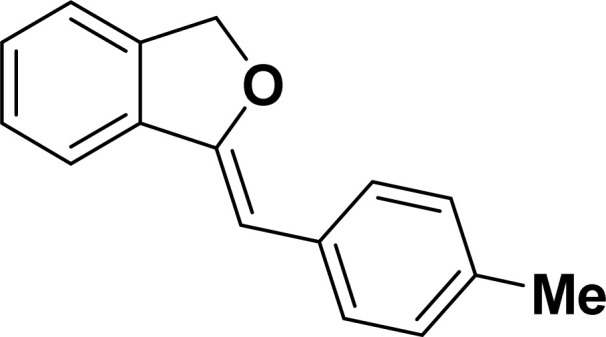	79	83
3	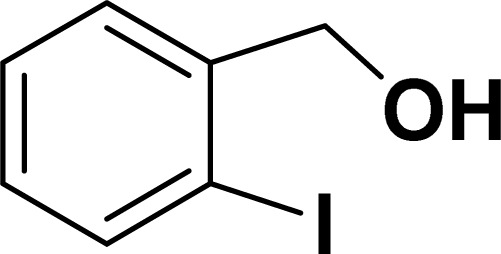	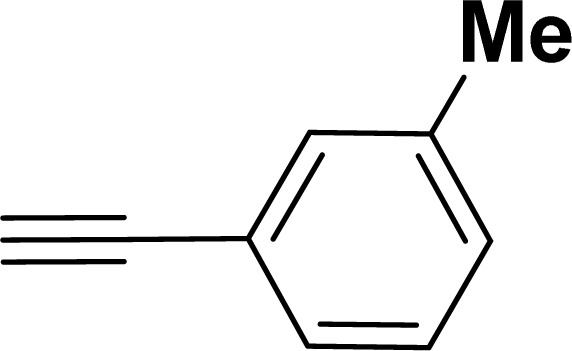	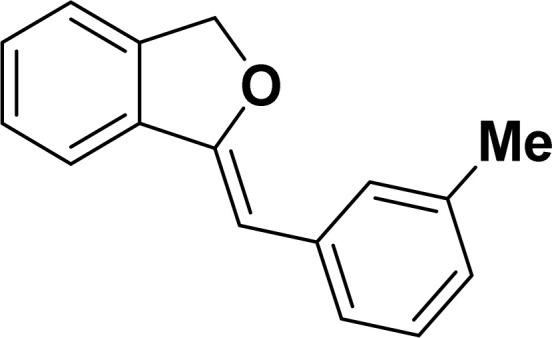	76	72
4	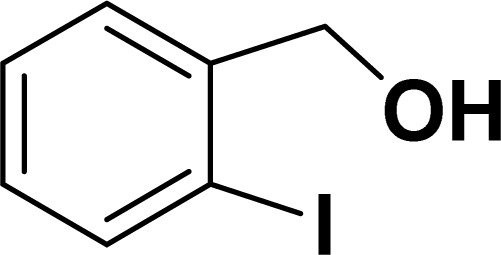	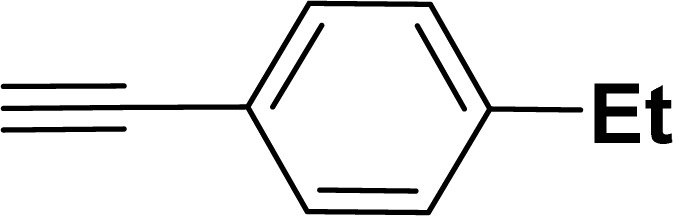	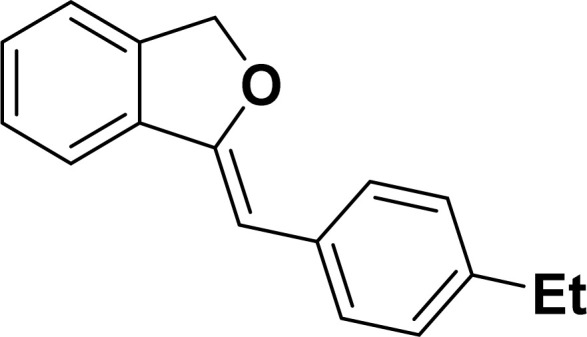	82	80
5	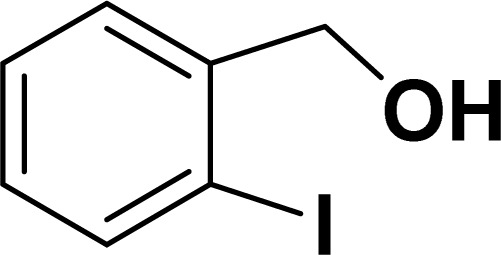	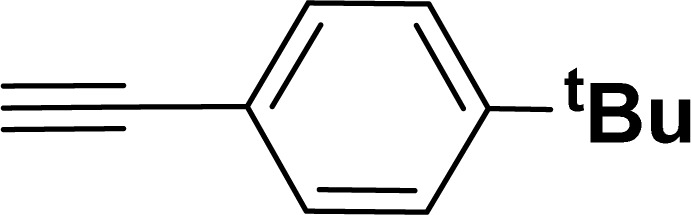	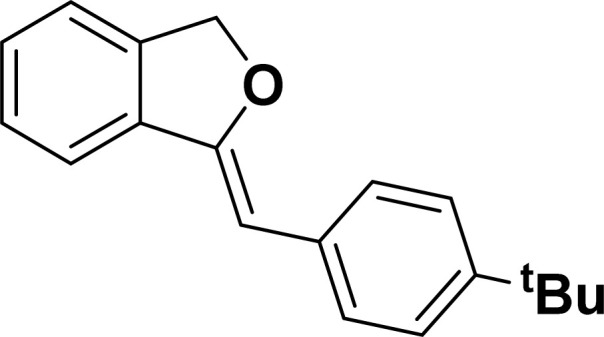	77	80
6	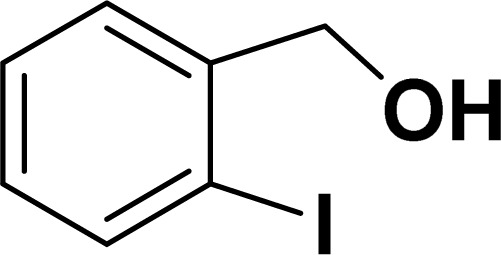	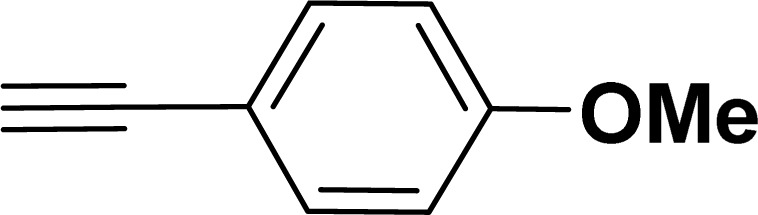	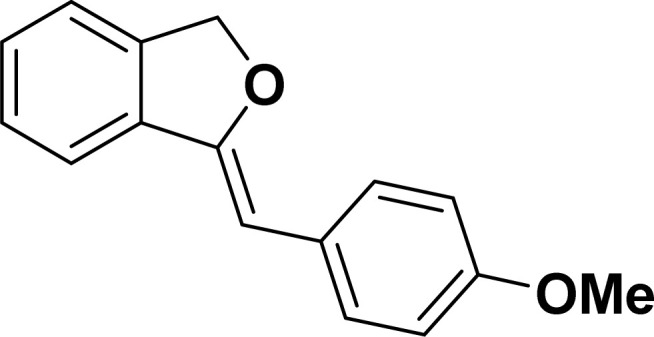	82	83

a Reaction conditions: 2-iodobenzyl alcohol (0.50 mmol), alkyne (0.60 mmol), 2.0 mol% of 2a or 2b, K_3_PO_4_ (1.00 mmol), DMSO (2 mL), 90 °C, 10 h.

b Isolated yield.

**Scheme 2:**
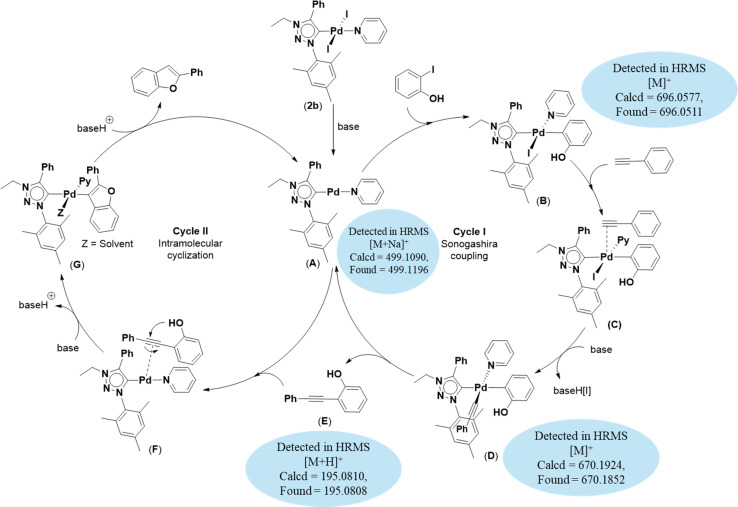
Proposed mechanism for the synthesis of benzofuran derivatives *via* domino Sonogashira coupling/cyclization reactions.

Based on previous literature reports and experimental observations,^7,42,44^ a plausible reaction mechanism pathway for the Sonogashira coupling/cyclization of 2-iodoarenes and terminal alkynes was proposed in Scheme 2.

Initially, the palladium(ii) complex 2b was reduced to palladium(0) intermediate A as an active species. In cycle I, oxidative addition of 2-iodoarene led to the formation of intermediate B. This intermediate B coordinates with phenylacetylene to form intermediate C which is converted to intermediate D upon deprotonation of the coordinated phenylacetylene moiety. Next, through reductive elimination of intermediate D, 2-(phenylethynyl)arene and intermediate A are generated. Now, in cycle II, 2-(phenylethynyl)arene coordinated with intermediate A to give intermediate F, which is converted to intermediate G upon base-assisted deprotonation followed by palladium-assisted 5-*endo*-dig cyclization. Finally, protonolysis of intermediate G afforded the desired benzofuran along with the regeneration of intermediate A. This procedure yielded intermediates A (calcd = 499.1090; found = 499.1196), B (calcd = 696.0577; found = 696.0511), D (calcd = 670.1924; found = 670.1852), and E (calcd = 195.0810; found = 195.0808) detected by high-resolution mass spectrometry (HRMS).

The comparison of catalytic activity of complexes 2a and 2b with the previously reported N-heterocyclic carbene-based palladium complexes for the synthesis of benzofuran from the 2-iodophenol and phenylacetylene *via* domino Sonogashira coupling/cyclization reactions was shown in Table 4. Initially, Peris and coworkers reported the domino synthesis of benzofuran *via* Sonogashira coupling/cyclization reactions using 1 mol% of 1,2,4-triazole derived PEPPSI complexes under copper- and amine-free conditions.^41^ Ghosh and coworkers group reported the same catalytic transformation employing 1 mol% of 1,2,4-triazole derived bis-NHC and phosphine ligand-based dipalladium complexes.^42^ Later, our group reported the same domino synthesis of benzofuran using 0.2 mol% of N-heterocyclic carbene ligated bimetallic palladium PEPPSI complexes.^43^ Recently, Bera group also developed an annellated mesoionic carbene ligand stabilized palladium complex and explored their catalytic activity in benzofuran synthesis *via* domino approach.^7^ Thus, a comparison of the performance with previously reported well-defined N-heterocyclic carbene-based palladium complexes revealed that these types of mesoionic palladium complexes were not as active as other normal N-heterocyclic carbene-based PEPPSI complexes in terms of catalyst loading.

**Table 4:** Comparison with reported well-defined catalysts for domino Sonogashira coupling/cyclization reaction of 2-iodophenol with phenylacetylene for synthesizing 2-phenylbenzofuran

No.	Catalyst	Loading (mol%)	Time (h)	Base/solvent	Temp. (°C)	Yield (%)	Ref.
1	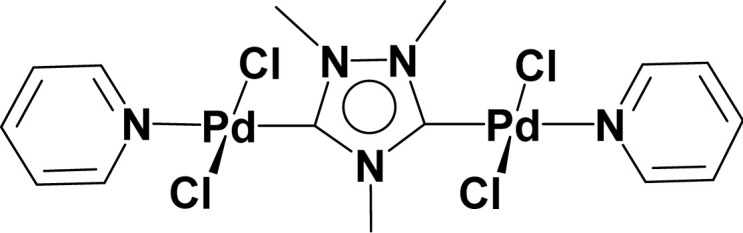	1 mol%	8	Cs_2_CO_3_/DMSO	80	93	41
2	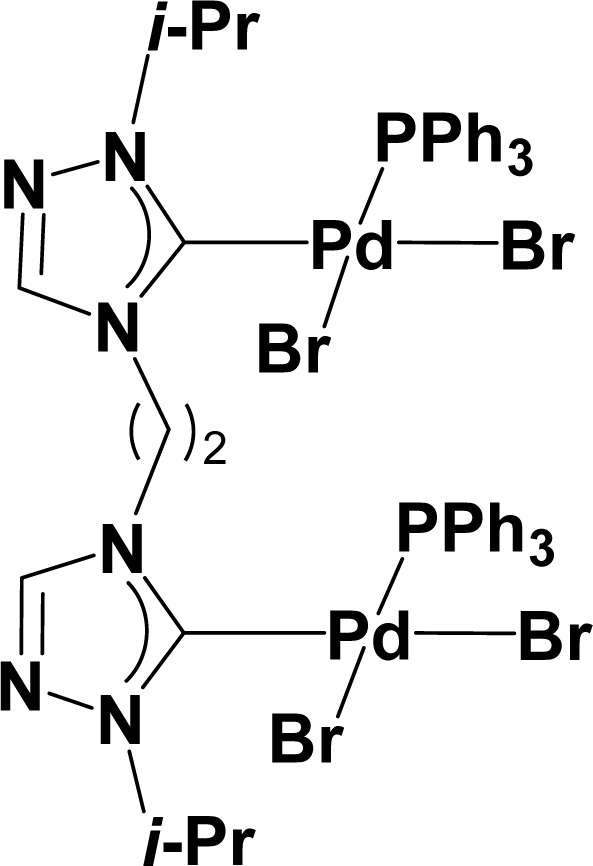	1 mol%	4	Cs_2_CO_3_/DMSO	80	81	42
3	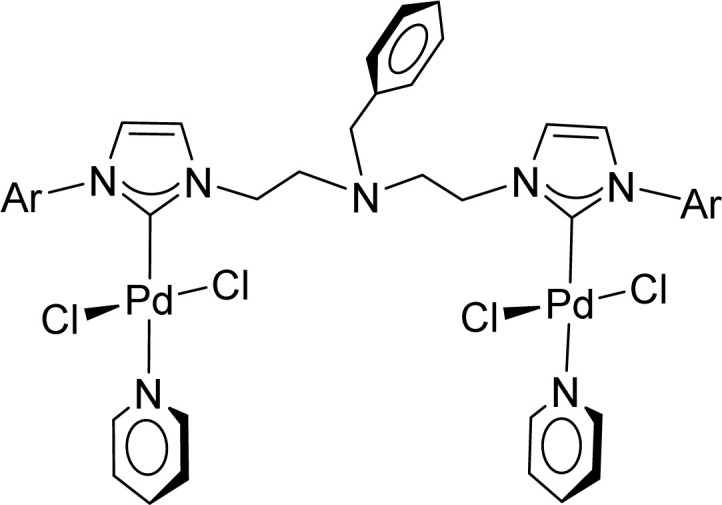	0.2 mol%	16	K_3_PO_4_/DMSO	90	>99	43
4	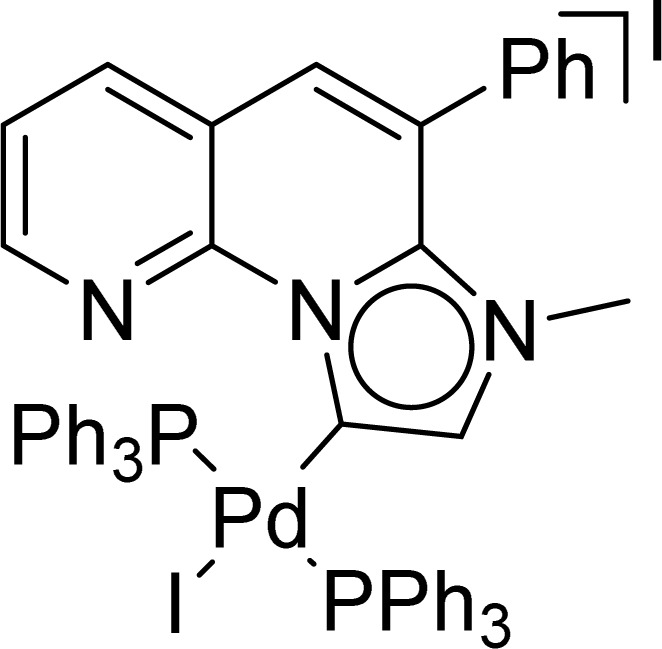	2 mol%	2	K_2_CO_3_/DMSO	90	100 (GC-MS yield)	7
5	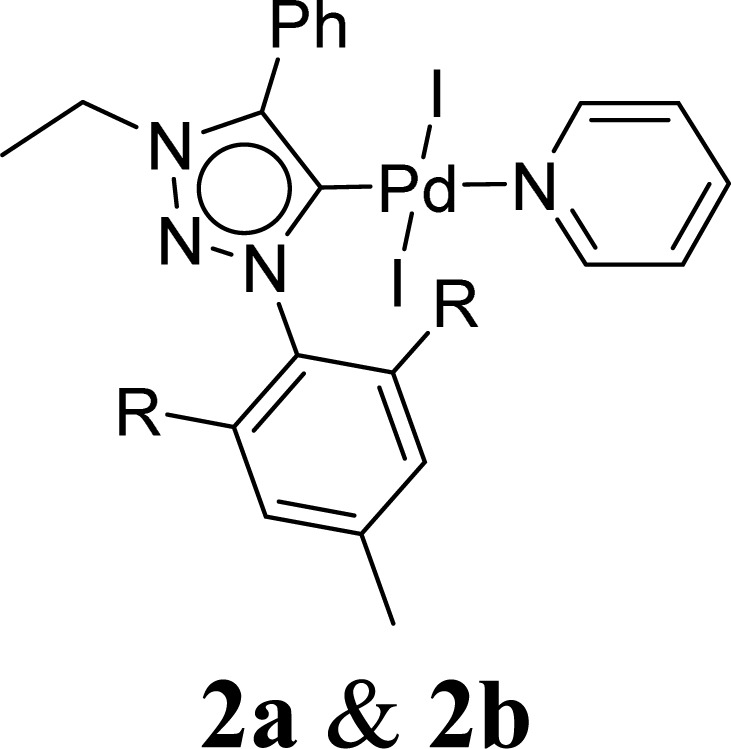	2 mol%	10	K_3_PO_4_/DMSO	90	83	This work

## Conclusions

We have reported the preparation and characterization of two 1,2,3-triazolylidene-based palladium PEPPSI complexes 2a and 2b and their application in domino Sonogashira coupling/cyclization reactions for the synthesis of benzofuran and indole derivatives. Both PEPPSI-themed palladium complexes were well characterized by spectroscopic techniques. The complex 2b was characterized by X-ray crystallography. The desired catalytic products were obtained in moderate to good yields with broad substrate scope at 2 mol% catalyst loading. This work further showed the catalytic potential of PEPPSI-themed complexes as effective catalysts for domino Sonogashira coupling/cyclization reactions.

## Experimental section

### General methods and materials

All manipulations were carried out under an atmosphere of nitrogen or in air. NMR spectra were recorded at 298 K on Bruker 500 MHz and JEOL 400 MHz NMR spectrometer. The chemical shifts of proton and carbon are reported in ppm and referenced using residual proton (7.26 ppm) and carbon signals (77.16 ppm) of CDCl_3_.^51^ NMR annotations used: br. = broad, d = doublet, m = multiplet, s = singlet, t = triplet, sept = septet. Elemental analyses were performed using Thermo Quest FLASH 2000 SERIES (CHNS) Elemental Analyzer. Mass data are collected from Agilent 6545 LC/Q-Tof spectrometer. Solvents were purchased from commercial suppliers and used without further purification. CCDC-2336820 (2b) contains the supplementary crystallographic data for this paper. The data can be obtained free of charge at https://www.ccdc.cam.ac.uk/structures/.† Catalytic substrates 2-iodophenol, (2-iodophenyl)methanol, phenylacetylene, 4-ethynyltoluene, 3-ethynyltoluene, 4-ethylphenylacetylene, 4-*tert*-butylphenylacetylene, 4-ethynylanisole, 3-ethynylaniline, prop-2-yn-1-ol, AgOTf, C_2_H_5_I, and K_3_PO_4_ were purchased from Sigma-Aldrich and used as received without further purification. 1-(*p*-Tolyl)-4-phenyl-1*H*-1,2,3-triazole,^45^ phenyl-1*H*-1,2,3-triazole,^40^ and *N*-(2-iodophenyl)-4-methylbenzenesulfonamide^52^ were synthesised using literature procedures.

### Synthesis of ligand 1a

C_2_H_5_I (0.955 g, 6.12 mmol), AgOTf (1.57 g, 6.12 mmol) and anhydrous toluene (20 mL) were added in an aluminum foil covered 50 mL round bottom flask. The resulting mixture was heated at 60 °C for 5 hours under nitrogen atmosphere. In another 50 mL round bottom flask, 1-(*p*-tolyl)-4-phenyl-1*H*-1,2,3-triazole (1.20 g, 5.10 mmol) was mixed with anhydrous toluene (10 mL). After 5 hours, the mixture of C_2_H_5_I and AgOTf was transferred into the triazole containing round bottom flask by glass syringe, and the resulting reaction mixture was heated at 100 °C for 3 days under nitrogen atmosphere. The reaction mixture was cooled to room temperature and was decanted off the solvent. The residue was purified by column chromatography (MeOH/CHCl_3_ = 2 : 98), affording the product as a dark brownish solid (1.48 g, 70%). ^1^H NMR (400 MHz, CDCl_3_): *δ* 8.97 (s, 1H), 7.81 (d, *J* = 8.0 Hz, 2H), 7.66 (d, *J* = 8.0 Hz, 2H), 7.50 (d, *J* = 8.0 Hz, 3H), 7.33 (d, *J* = 8.0 Hz, 2H), 4.64 (q, *J* = 7.0 Hz, 2H), 2.39 (s, 3H), 1.58 (t, *J* = 7.0 Hz, 3H). ^13^C{^1^H} NMR (100 MHz, CDCl_3_): *δ* 143.7, 142.5, 132.6, 131.8, 130.8, 129.6, 129.6, 126.5, 121.7, 121.4, 47.9, 21.3, 14.1 ppm. IR data (KBr pellet) cm^−1^: 3094 (w), 2994 (w), 2947 (w), 2923 (w), 1613 (w), 1571 (w), 1518 (w), 1494 (w), 1457 (w), 1378 (w), 1265 (s), 1224 (m), 1153 (s), 1032 (m), 821 (w), 765 (w), 695 (w), 637 (w), 573 (w). HRMS (ESI) calcd for C_18_H_18_F_3_N_3_O_3_S^+^ [M + H]^+^*m*/*z* 414.1093; found 414.1116.

#### Synthesis of (1-ethyl-3(*p*-tolyl)5-phenyl-2,3-dihydro-1*H*-1,2,3-triazol-4-yl) (pyridin-2yl) palladium(ii) iodide (2a)

Ligand 1a (0.349 g, 0.844 mmol), PdCl_2_ (0.125 g, 0.704 mmol), KI (0.246 g, 1.48 mmol), K_2_CO_3_ (0.486 g, 3.52 mmol) and pyridine (4 mL) were added in 50 mL round bottom flask and the reaction mixture was heated at 110 °C for 5 days. The reaction mixture was cooled down to room temperature and washed with saturated copper sulphate solution (5 × 10 mL) (for the removal of unbounded pyridine) and the organic fraction was extracted in chloroform (3 × 20 mL). Afterwards, the organic fraction was dried on MgSO_4_ and filtered on a Celite-pad. The resulting crude product was further purified by column chromatography (20 : 80, EtOAc : petroleum ether), affording desired product as an orange solid (0.366 g, 74%). ^1^H NMR (400 MHz, CDCl_3_): 8.78 (d, *J* = 8.0 Hz, 2H), 8.34 (d, *J* = 8.0 Hz, 2H), 7.95 (d, *J* = 7.0 Hz, 2H), 7.60–7.55 (dd, *J* = 14.6, 7.2 Hz, 4H), 7.42 (d, *J* = 8.0 Hz, 2H), 7.22–7.15 (m, 2H), 4.34 (q, *J* = 7.3 Hz, 2H), 2.48 (s, 3H), 1.57–1.53 (m, 3H). ^13^C{^1^H} NMR (100 MHz, CDCl_3_): *δ* 153.8, 143.6, 140.1, 137.5, 137.3, 134.4 (MIC-Pd), 130.8, 130, 129.5, 128.8, 127.6, 125.2, 124.2, 46.2, 21.5, 14.7 ppm. IR data (KBr pellet) cm^−1^: 2984 (w), 1740 (w), 1599 (w), 1509 (w), 1444 (m), 1374 (w), 1291 (m), 1188 (m), 1071 (m), 1019 (m), 815 (m), 764 (s), 692 (s), 595 (w), 519 (w), 475 (w), 421 (w). HRMS (ESI) calcd for C_22_H_22_I_2_N_4_Pd^+^ [M + Na]^+^*m*/*z* 724.8867; found 724.8820. Anal. calcd for C_22_H_22_I_2_N_4_Pd*:* C, 37.61; H, 3.16; N, 7.97; found: C, 37.59; H, 3.12; N, 7.42%.

#### Synthesis of ligand 1b

C_2_H_5_I (0.853 g, 5.46 mmol), AgOTf (1.40 g, 5.46 mmol) and anhydrous toluene (20 mL) were added in an aluminum foil covered 50 mL round bottom flask and the mixture was heated at 60 °C for 5 hours under nitrogen atmosphere. In another 50 mL round bottom flask, 1-mesityl-4-phenyl-1*H*-1,2,3-triazole (1.20 g, 4.55 mmol) was suspended in anhydrous toluene (10 mL). After 5 hours, the reaction mixture of C_2_H_5_I and AgOTf was transferred into the triazole containing round bottom flask by glass syringe, and the resulting reaction mixture was heated at 100 °C for 3 days under nitrogen atmosphere. The reaction mixture was cooled to room temperature and solvent was decanted off. The residue was purified by column chromatography (SiO_2_ : MeOH/CHCl_3_ = 2 : 98), affording the product as a dark brownish solid (1.61 g, 80%). ^1^H NMR (500 MHz, CDCl_3_): 8.60 (s, 1H), 7.76 (d, *J* = 6.0 Hz, 2H), 7.55 (s, 3H), 7.02 (s, 2H), 4.76 q, *J* = 7.0 Hz, 2H), 2.34 (s, 3H), 2.08 (s, 6H), 1.58 (t, *J* = 7.0 Hz, 3H). ^13^C{^1^H} NMR(125 MHz, CDCl_3_): *δ* 143.8, 142.5, 134.4, 132.0, 131.4, 130.3, 129.89, 129.84, 129.77, 121.5, 48.3, 21.1, 17.2, 14.0 ppm. IR data (KBr pellet) cm^−1^: 3096 (m), 2924 (w), 2855 (w), 1615 (w), 1459 (w), 1267 (s), 1155 (m), 1030 (m), 637 (w). HRMS (ESI) calcd for C_20_H_22_F_3_N_3_O_3_S^+^ [M + H]^+^*m*/*z* 442.1406; found 442.1396.

#### Synthesis of (1-ethyl-3-mesityl-5-phenyl-2,3-dihydro-1*H*-1,2,3-triazol-4-yl)(pyridin-2yl)palladium(ii) iodide (2b)

Ligand 1b (0.267 g, 0.609 mmol), PdCl_2_ (0.090 g, 0.507 mmol), KI (0.176 g, 1.06 mmol), K_2_CO_3_ (0.351 g, 2.54 mmol) and pyridine (*ca.* 4 mL) were added in 50 mL round bottom flask and the reaction mixture was heated at 110 °C for 5 days. The reaction mixture was cooled down to room temperature and washed with saturated copper sulphate solution (5 × 10 mL) (to remove unbounded pyridine), and the organic fraction was extracted in chloroform. Afterward, the organic fraction was dried on MgSO_4_ and filtered on a Celite-pad. The resulting crude product was further purified by column chromatography (EtOAc : petroleum ether = 20 : 80) affording the desired product as an orange solid (0.308 g, 83%). ^1^H NMR (400 MHz, CDCl_3_): *δ* 8.67–8.65 (m, 2H), 8.00–7.98 (m, 2H), 7.63–7.53 (m, 4H), 7.14–7.11 (m, 2H), 7.05 (s, 2H), 4.42 (q, *J* = 7.0 Hz, 2H), 2.40 (s, 3H), 2.39 (s, 6H), 1.50 (t, *J* = 7.0 Hz, 3H). ^13^C{^1^H} NMR (100 MHz, CDCl_3_): *δ* 153.7, 144.7, 140.3, 137.5, 137.1, 135.7, 135.6 (MIC-Pd), 131.1, 130, 129.5, 128.6, 128.1, 124, 46.1, 21.4, 21.3, 14.8 ppm. IR data (KBr pellet) cm^−1^: 2921 (w), 1739 (w), 1600 (w), 1442 (m), 1371(m), 1284 (m), 1228 (w), 1174 (m), 1084 (m), 1025 (m), 853 (w), 760 (s), 693 (s), 600 (w), 548 (w), 489 (w). HRMS (ESI) calcd for C_24_H_26_I_2_N_4_Pd^+^ [M + K]^+^*m*/*z* 768.8919; found 768.8943. Anal. calcd for C_24_H_26_I_2_N_4_Pd: C, 39.45; H, 3.59; N, 7.67; found: C, 38.90; H, 3.56; N, 7.20. Suitable single crystal for X-ray diffraction analysis of 2b was obtained by slow evaporation of acetonitrile solution at room temperature.

### General procedure for Sonogashira coupling/cyclization reactions of 2-iodoarenes and alkyne

In a reaction vial, 2-iodoarenes (0.50 mmol), phenylacetylene (0.60 mmol), and K_3_PO_4_ (1.00 mmol) were added. A palladium complex (2a or 2b, 2 mol%) and DMSO (*ca*. 2 mL) were added, and the resulting reaction mixture was heated at 90 °C for 10 hours. The reaction mixture was cooled to room temperature and quenched with water (*ca*. 20 mL). The resulting reaction mixture was extracted with EtOAc (3 × 20 mL) and dried on anhydrous Na_2_SO_4_. Solvent was evaporated under reduced pressure and further purification was done by column chromatography.

#### 2-Phenylbenzofuran (3aa: Table 2, entry 1)^44^


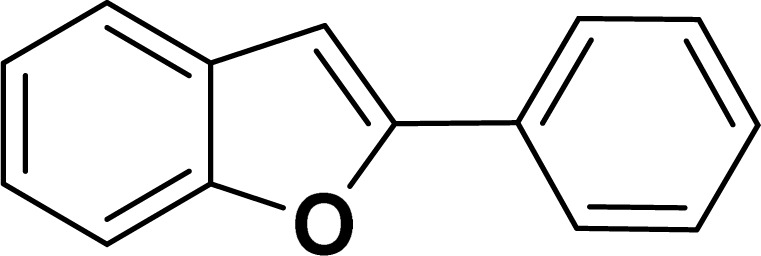
 ^1^H NMR (500 MHz, CDCl_3_): *δ* 7.92–7.87 (m, 2H), 7.60 (d, *J* = 7.5 Hz, 1H), 7.56–7.54 (m, 1H), 7.48–7.45 (m, 2H), 7.40–7.35 (m, 1H), 7.33–7.28 (m, 1H), 7.28–7.23 (m, 1H), 7.04 (s, 1H) ppm.

#### 2-(*p*-Tolyl)benzofuran (3ab: Table 2, entry 2)^44^


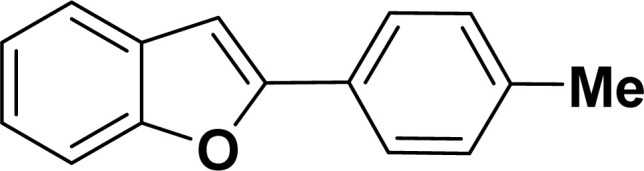
 ^1^H NMR (500 MHz, CDCl_3_): *δ* 7.79 (d, *J* = 8.0 Hz, 2H), 7.61–7.58 (m, 1H), 7.54 (d, *J* = 8.0 Hz, 1H), 7.45 (d, *J* = 8.0 Hz, 1H), 7.31–7.23 (m, 2H), 7.16 (d, *J* = 8.0 Hz, 1H), 6.98 (s, 1H), 2.42 (s, 3H) ppm.

#### 2-(*m*-Tolyl)benzofuran (3ac: Table 2, entry 3)^44^


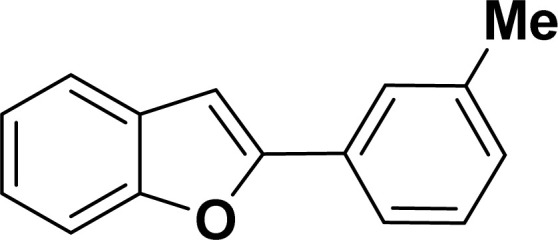
 ^1^H NMR (500 MHz, CDCl_3_): *δ* 7.76 (s, 1H), 7.73 (d, *J* = 8.0 Hz, 1H), 7.63 (d, *J* = 8.0 Hz, 1H), 7.58 (d, *J* = 8.0 Hz, 1H), 7.40 (d, *J* = 8.0 Hz, 1H), 7.34 (t, *J* = 8.0 Hz, 1H), 7.29–7.26 (m, 1H), 7.22 (d, *J* = 8.0 Hz, 1H), 7.06 (s, 1H), 2.48 (s, 3H) ppm.

#### 2-(4-Ethylphenyl)benzofuran (3ad: Table 2, entry 4)^44^


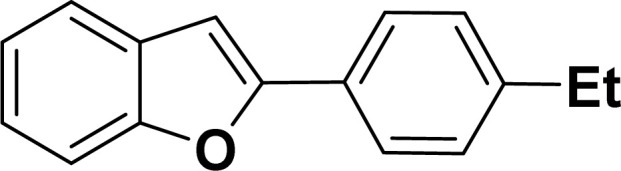
 ^1^H NMR (500 MHz, CDCl_3_): *δ* 7.88 (d, *J* = 8.0 Hz, 2H), 7.66 (d, *J* = 7.5 Hz, 1H), 7.62 (d, *J* = 8.0 Hz, 1H), 7.55 (d, *J* = 8.0 Hz, 1H), 7.37 (d, *J* = 8.0 Hz, 2H), 7.34–7.30 (m, 1H), 7.05 (s, 1H), 2.78 (q, *J* = 7.0 Hz, 2H), 1.37 (t, *J* = 7.0 Hz, 3H) ppm.

#### 2-(4-*tert*-Butylphenyl)benzofuran (3ae: Table 2, entry 5)^44^


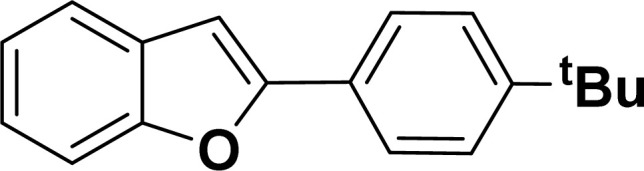
 ^1^H NMR (500 MHz, CDCl_3_): *δ* 7.86 (d, *J* = 8.0 Hz, 2H), 7.62 (d, *J* = 7.5 Hz, 1H), 7.58 (d, *J* = 8.0 Hz, 1H), 7.53 (d, *J* = 8.0 Hz, 2H), 7.32 (t, *J* = 7.5 Hz, 1H), 7.28–7.26 (m, 1H), 7.03 (s, 1H), 1.42 (s, 9H) ppm.

#### 3-(Benzofuran-2-yl)aniline (3af: Table 2, entry 6)^44^


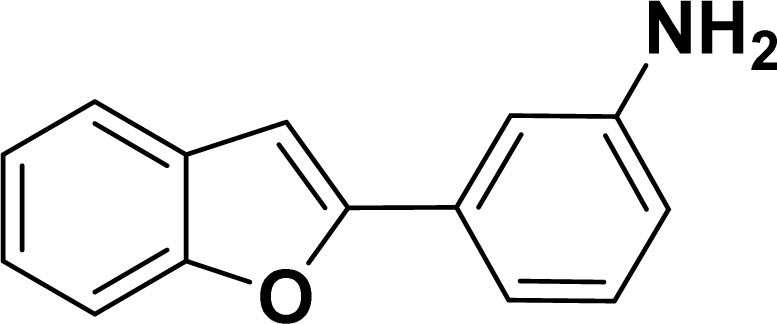
 ^1^H NMR (500 MHz, CDCl_3_): *δ* 7.60 (d, *J* = 7.5 Hz, 1H), 7.55 (d, *J* = 8.0 Hz, 1H), 7.34–7.21 (m, 5H), 7.00 (s, 1H), 6.70–6.69 (m, 1H), 3.70 (br s, 2H) ppm.

#### Benzofuran-2-ylmethanol (3ag: Table 2, entry 7)^43^


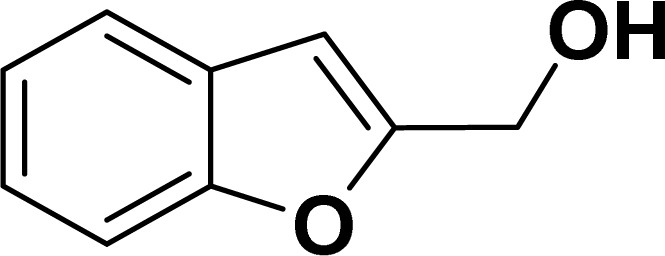
 ^1^H NMR (500 MHz, CDCl_3_): *δ* 7.58 (d, *J* = 8.0 Hz, 1H), 7.49 (d, *J* = 8.0 Hz, 1H), 7.37–7.27 (m, 2H), 7.26–7.16 (m, 1H), 6.69 (s, 1H), 4.80 (s, 2H) ppm.

#### 2-Phenyl-1-tosyl-1*H*-indole (3ah: Table 2, entry 8)^19^


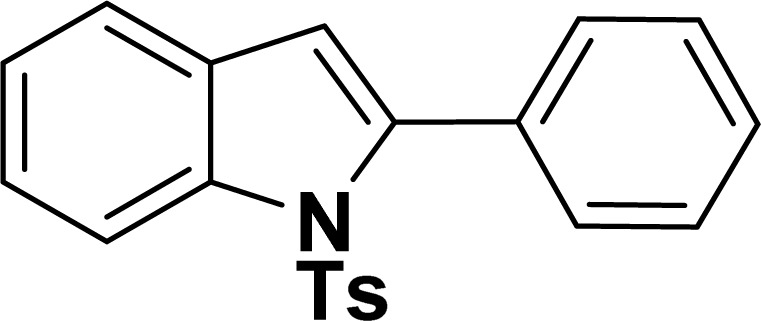
 ^1^H NMR (500 MHz, CDCl_3_): *δ* 8.23 (d, *J*_HH_ = 7.0 Hz, 1H), 7.42–7.35 (m, 6H), 7.27–7.18 (m, 4H), 6.96–6.95 (m, 2H), 6.46 (s, 1H), 2.20 (s, 3H) ppm. ^13^C{^1^H} NMR (125 MHz, CDCl_3_): *δ* 144.6, 142.2, 138.4, 134.9, 132.5, 130.6, 130.4, 129.3, 128.7, 127.6, 126.9, 124.8, 124.4, 120.8, 116.7, 113.6, 21.6 ppm.

#### 2-(4-Tolyl)-1-tosyl-1*H*-indole (3ai: Table 2, entry 9)^53^


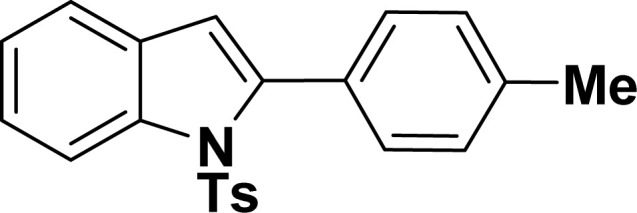
 ^1^H NMR (500 MHz, CDCl_3_): *δ* 8.22 (d, *J*_HH_ = 8.0 Hz, 2H), 7.35–7.31 (m, 3H), 7.27–7.24 (m, 1H), 7.21–7.14 (m, 5H), 6.96–6.94 (m, 2H), 6.42 (s, 1H), 2.36 (s, 3H), 2.19 (s, 3H) ppm. ^13^C{^1^H} NMR (125 MHz, CDCl_3_): *δ* 144.5, 142.4, 138.7, 138.3, 134.8, 130.7, 130.3, 129.6, 129.2, 128.3, 126.9, 124.7, 124.3, 120.7, 116.8, 113.3, 29.8, 21.5 ppm.

#### 2-(3-Tolyl)-1-tosyl-1*H*-indole (3aj: Table 2, entry 10)^54^


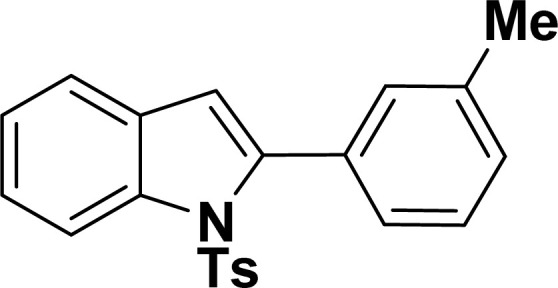
 ^1^H NMR (500 MHz, CDCl_3_): *δ* 8.22 (d, *J*_HH_ = 8.0 Hz, 1H), 7.35–7.34 (m, 1H), 7.27–7.24 (m, 1H), 7.23–7.18 (m, 5H), 7.16–7.15 (m, 2H), 6.96–6.94 (m, 2H), 6.44 (s, 1H), 2.33 (s, 3H), 2.20 (s, 3H) ppm. ^13^C{^1^H} NMR (125 MHz, CDCl_3_): *δ* 144.5, 142.4, 138.3, 137.0, 134.9, 132.4, 131.1, 130.6, 129.5, 129.2, 127.55, 127.5, 126.9, 124.7, 124.3, 120.7, 116.7, 113.4, 29.8, 21.6 ppm.

#### 2-(4-Ethylphenyl)-1-tosyl-1*H*-indole (3ak: Table 2, entry 11)^55^


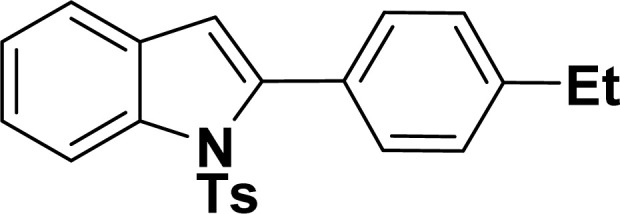
 ^1^H NMR (500 MHz, CDCl_3_): *δ* 8.21 (d, *J*_HH_ = 8.0 Hz, 1H), 7.33–7.31 (m, 3H), 7.25–7.22 (m, 1H), 7.18–7.15 (m, 5H), 6.93–6.91 (m, 2H), 6.41 (s, 1H), 2.64 (q, *J*_HH_ = 7.0 Hz, 2H), 2.17 (s, 3H), 1.21 (*t*, *J*_HH_ = 7.0 Hz, 3H) ppm. ^13^C{^1^H} NMR (125 MHz, CDCl_3_): *δ* 144.9, 144.5, 142.4, 138.3, 134.8, 130.7, 130.3, 129.8, 129.2, 127.1, 126.9, 124.7, 124.3, 120.6, 116.7, 113.3, 28.8, 21.5, 15.4 ppm.

#### 2-(4-(*tert*-Butyl)phenyl)-1-tosyl-1*H*-indole (3al: Table 2, entry 12)^56^


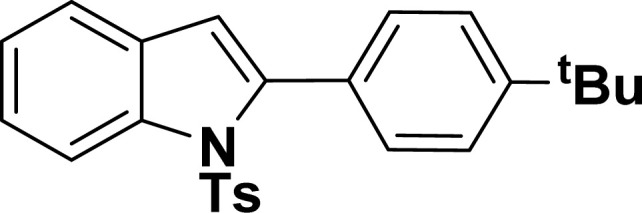
 ^1^H NMR (500 MHz, CDCl_3_): *δ* 8.22 (d, *J*_HH_ = 8.0 Hz, 1H), 7.35–7.34 (m, 4H), 7.27–7.24 (m, 1H), 7.20–7.16 (m, 4H), 6.96–6.94 (m, 2H), 6.44 (s, 1H), 2.20 (s, 3H), 1.31 (s, 9H) ppm. ^13^C{^1^H} NMR (125 MHz, CDCl_3_): *δ* 151.8, 144.5, 142.4, 138.4, 134.8, 130.7, 130.1, 129.5, 129.2, 127.0, 124.7, 124.5, 124.3, 120.7, 116.8, 113.4, 34.8, 31.5, 21.6 ppm.

#### 3-(1-Tosyl-1*H*-indol-2-yl)aniline (3am: Table 2, entry 13)


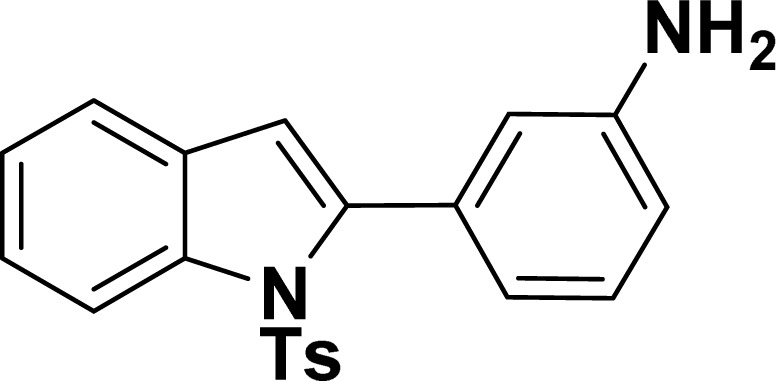
 ^1^H NMR (500 MHz, CDCl_3_): *δ* 8.21 (d, *J*_HH_ = 8.0 Hz, 1H), 7.34–7.33 (m, 1H), 7.27–7.23 (m, 3H), 7.18–7.14 (m, 1H), 7.12–7.09 (m, 1H), 6.96–6.94 (m, 2H), 6.79–6.77 (m, 1H), 6.75 (s, 1H), 6.67–6.66 (m, 1H), 6.44 (s, 1H), 3.54 (br s, 2H), 2.19 (s, 3H) ppm. ^13^C{^1^H} NMR (125 MHz, CDCl_3_): *δ* 145.6, 144.5, 142.5, 138.4, 134.8, 133.4, 130.6, 129.2, 128.5, 127.0, 124.7, 124.3, 120.77, 120.76, 117.4, 116.7, 115.6, 113.3, 21.6 ppm. HRMS (ESI) calcd for C_21_H_18_N_2_O_2_S^+^ [M + H]^+^*m*/*z* 363.1169; found 363.1163.

#### 2-Hydromethanene-1-tosyl-1*H*-indole (3an: Table 2, entry 14)^19^


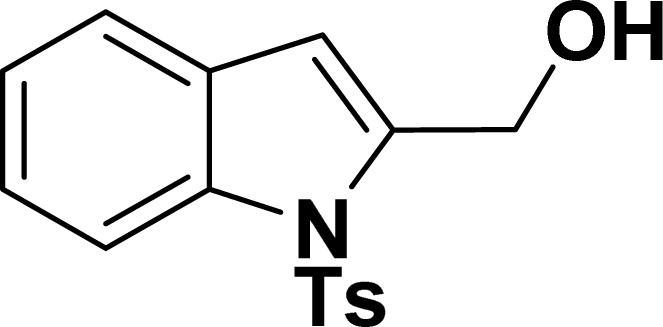
 ^1^H NMR (500 MHz, CDCl_3_): *δ* 7.97 (d, *J*_HH_ = 8.0 Hz, 1H), 7.63 (d, *J*_HH_ = 8.0 Hz, 2H), 7.39 (d, *J*_HH_ = 8.0 Hz, 1H), 7.23–7.19 (m, 1H), 7.17–7.10 (m, 3H), 6.55 (s, 1H), 4.82 (s, 2H), 3.10 (br s, 1H), 2.24 (s, 3H) ppm. ^13^C{^1^H} NMR (125 MHz, CDCl_3_): *δ* 145.2, 140.3, 137.1, 135.7, 130, 129.2, 126.5, 125, 123.8, 121.2, 114.4, 111.3. 58.6, 21.6 ppm.

#### 1-[(*Z*)-Phenylmethylidene]isobenzofuran (4aa: Table 3, entry 1)^57^


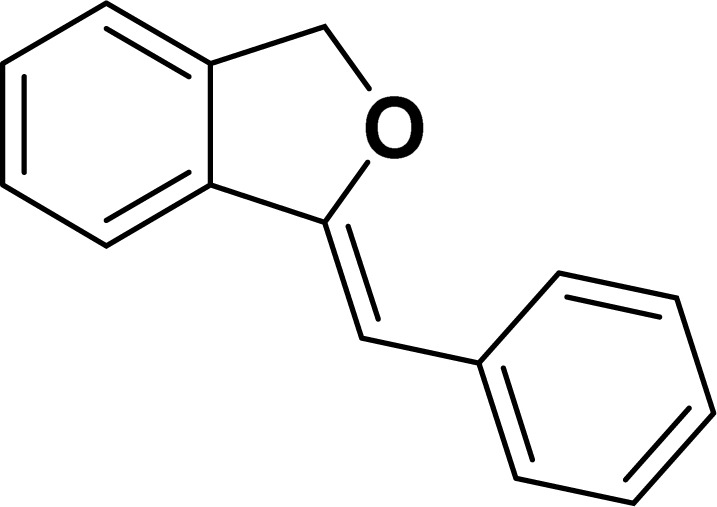
 ^1^H NMR (500 MHz, CDCl_3_): *δ* 7.78–7.77 (m, 2H), 7.60–7.59 (m, 1H), 7.38–7.35 (m, 5H), 7.19–7.16 (m, 1H), 5.98 (s, 1H), 5.54 (s, 2H) ppm. ^13^C{^1^H} NMR (125 MHz, CDCl_3_): *δ* 156.3, 139.3, 136.4, 134.9, 128.8, 128.4, 128.1, 127.8, 125.3, 121.2, 120.0, 96.3, 74.9, 29.7 ppm.

#### 1-[(*Z*)-4-(Methylphenyl)methylidene]isobenzofuran (4ab: Table 3, entry 2)^57^


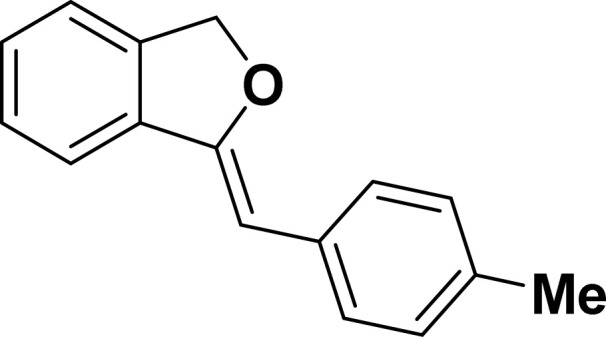
 ^1^H NMR (500 MHz, CDCl_3_): *δ* 7.56–7.54 (m, 2H), 7.47–7.46 (m, 1H), 7.26–7.23 (m, 3H), 7.07–7.06 (m, 2H), 5.84 (s, 1H), 5.42 (s, 2H), 2.26 (s, 3H) ppm. ^13^C{^1^H} NMR (125 MHz, CDCl_3_): *δ* 155.7, 139.2, 135.1, 135.0, 133.6, 129.2, 128.6, 128.1, 127.8, 121.2, 119.9, 96.3, 74.9, 21.3 ppm.

#### 1-[(*Z*)-3-(Methylphenyl)methylidene]isobenzofuran (4ac: Table 3, entry 3)^50^


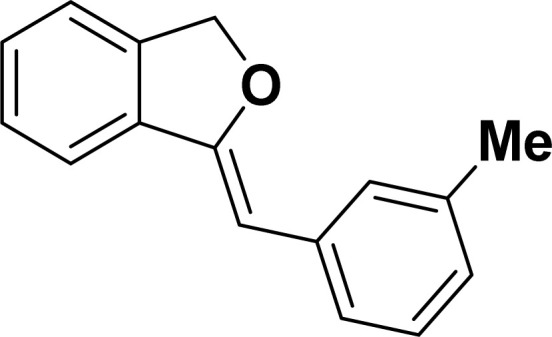
 ^1^H NMR (500 MHz, CDCl_3_): *δ* 7.48–7.44 (m, 3H), 7.26–7.20 (m, 3H), 7.15–7.12 (m, 1H), 6.88–6.87 (m, 1H), 5.82 (s, 1H), 5.40 (s, 2H), 2.28 (s, 3H) ppm. ^13^C{^1^H} NMR (125 MHz, CDCl_3_): *δ* 156.2, 139.3, 137.8, 136.3, 135.0, 128.7, 128.5, 128.3, 128.1, 126.2, 125.0, 121.2, 120.0, 96.4, 74.9, 21.6 ppm.

#### 1-[(*Z*)-4-(Ethylphenyl)methylidene]isobenzofuran (4ad: Table 3, entry 4)^57^


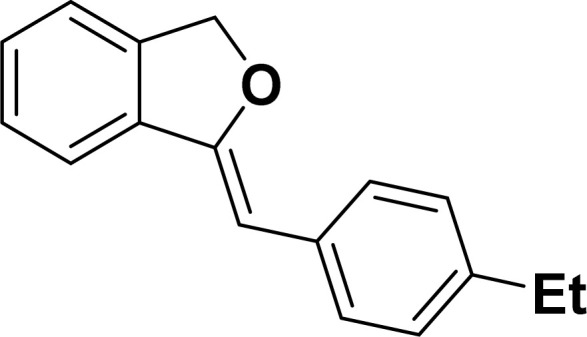
 ^1^H NMR (500 MHz, CDCl_3_): *δ* 7.59–7.57 (m, 2H), 7.48–7.47 (m, 1H), 7.28–7.24 (m, 3H), 7.10–7.08 (m, 2H), 5.85 (s, 1H), 5.42 (s, 2H), 2.56 (q, *J*_HH_ = 7.5 Hz, 2H), 1.16 (t, *J*_HH_ = 7.5 Hz, 3H) ppm. ^13^C{^1^H} NMR (125 MHz, CDCl_3_): *δ* 155.7, 141.5, 139.2, 135.1, 133.8, 128.6, 128.1, 128.0, 127.8, 121.2, 119.9, 96.3, 74.8, 28.7, 15.7 ppm.

#### 1-[(*Z*)-4-(*tert*-Butylphenyl)methylidene]isobenzofuran (4ae: Table 3, entry 5)


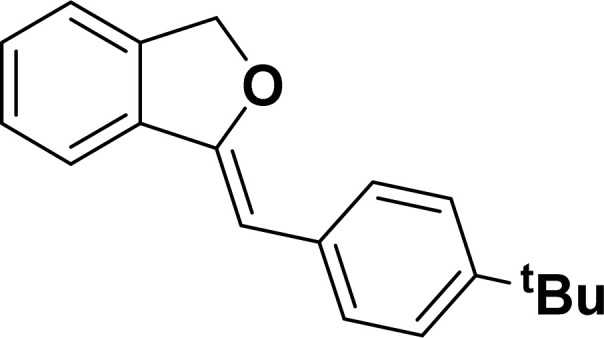
 ^1^H NMR (500 MHz, CDCl_3_): *δ* 7.60–7.58 (m, 2H), 7.47–7.45 (m, 1H), 7.29–7.27 (m, 2H), 7.26–7.21 (m, 3H), 5.85 (s, 1H), 5.40 (s, 2H), 1.24 (s, 9H) ppm. ^13^C{^1^H} NMR (125 MHz, CDCl_3_): *δ* 155.9, 148.3, 139.3, 135.1, 133.6, 128.6, 128.1, 127.6, 125.3, 121.2, 120, 96.1, 74.8, 34.6, 31.4 ppm. HRMS (ESI) calcd for C_19_H_20_O^+^ [M − H]^+^*m*/*z* 263.1434; Found 263.1432.

#### 1-[(*Z*)-4-(Methoxyphenyl)methylidene]isobenzofuran (4af: Table 3, entry 6)^58^


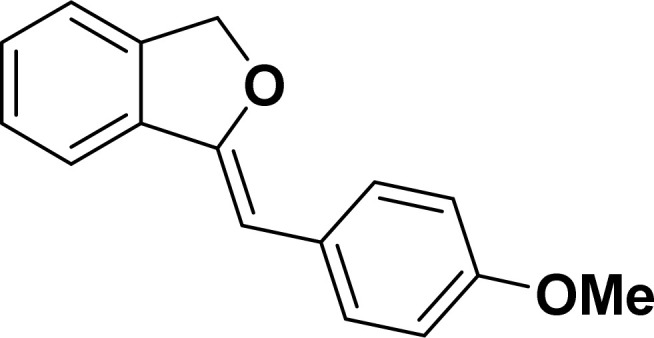
 ^1^H NMR (500 MHz, CDCl_3_): *δ* 7.60–7.59 (m, 2H), 7.45–7.44 (m, 1H), 7.25–7.22 (m, 3H), 6.81–6.80 (m, 2H), 5.82 (s, 1H), 5.41 (s, 2H), 3.73 (s, 3H) ppm. ^13^C{^1^H} NMR (125 MHz, CDCl_3_): *δ* 157.5, 154.8, 139, 135.1, 129.3, 129, 128.4, 128.1, 121.2, 119.7, 114, 95.9, 74.7, 55.3 ppm.

## Conflicts of interest

There are no conflicts to declare.

## Supplementary Material

RA-014-D4RA03485F-s001

RA-014-D4RA03485F-s002
