# Supplementary material for: Palladium(ii) complexes bearing mesoionic carbene ligands: catalytic application in domino Sonogashira coupling/cyclization reactions for one-pot synthesis of benzofuran and indole derivatives
Source: RSC Adv. 2024 Aug 27;14(37):27141–52. doi: 10.1039/d4ra03485f (PMC11348387; doi:10.1039/d4ra03485f)
Supplement: RA-014-D4RA03485F-s001 [file RA-014-D4RA03485F-s001.pdf]

Supplementary Information (SI) for RSC Advances.
This journal is © The Royal Society of Chemistry 2024

Supporting Information

Palladium(II) Complexes bearing Mesoionic Carbene Ligands: Catalytic Application in Domino Sonogashira Coupling/Cyclization Reactions for One-pot Synthesis of Benzofuran and Indole Derivatives

Om Prakash Joshi,[a] Ramalingam Thirumoorthi,[a] Ram T. Pardasani,[a] Sriparna Ray,[b]* and Chandrakanta Dash*[a]

[a]Department of Chemistry, School of Chemical Sciences and Pharmacy, Central University of Rajasthan, Bandarsindri, Ajmer-305817, Rajasthan, India.

[b]Catalytic Applications Laboratory, Department of Chemistry, School of Basic Sciences, Faculty of Science, Manipal University Jaipur, Dehmi Kalan, Jaipur – 303007, Rajasthan, India.

*Corresponding author:
Email: ckdash@curaj.ac.in; sriparna.ray@gmail.com

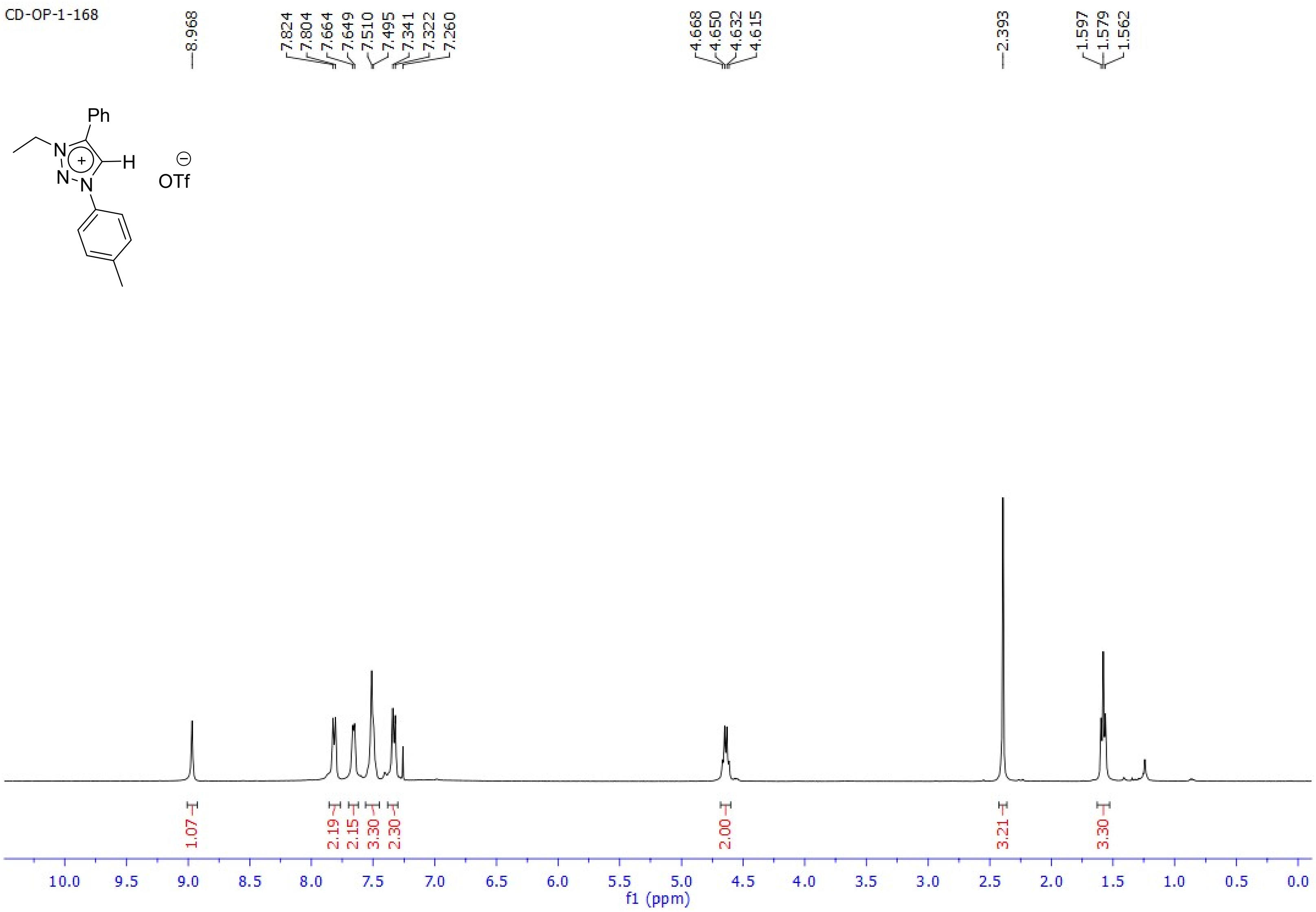

Figure S1. ^{1}H NMR spectrum of **1a** in $CDCl_3$

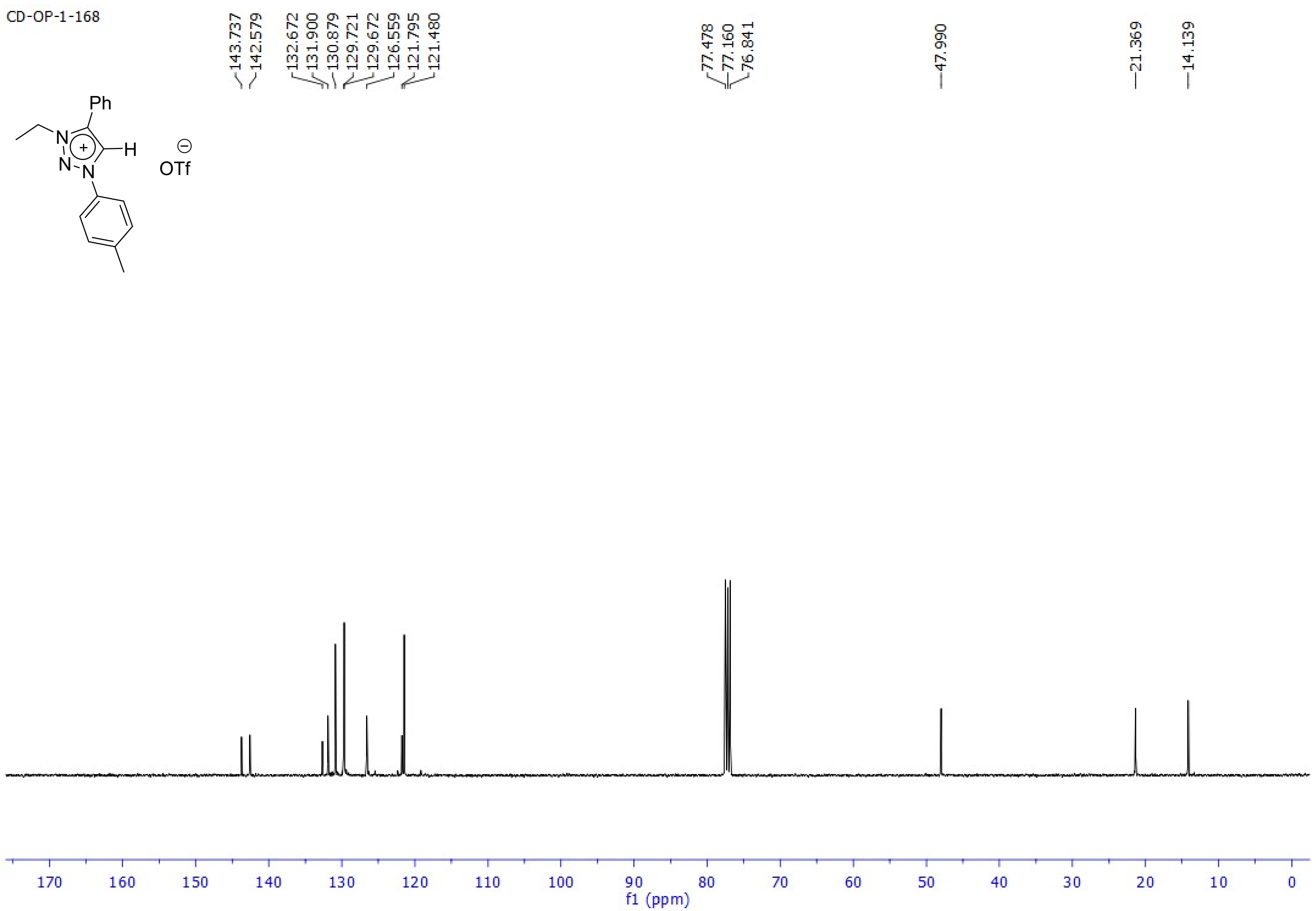

Figure S2. $^{13}C\{^{1}H\}$ NMR spectrum of **1a** in $CDCl_3$

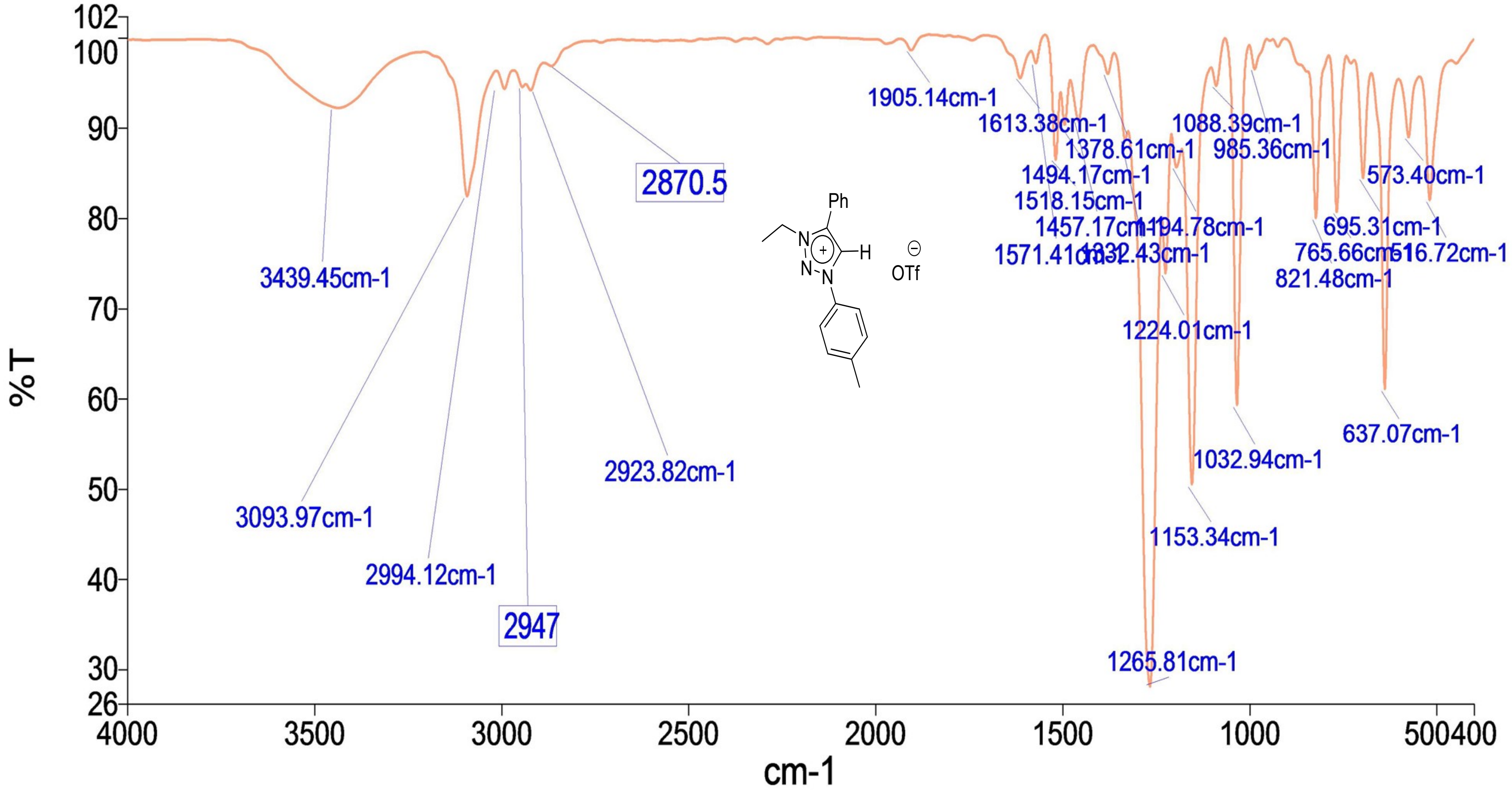

Figure S3. IR spectrum of **1a** in KBr

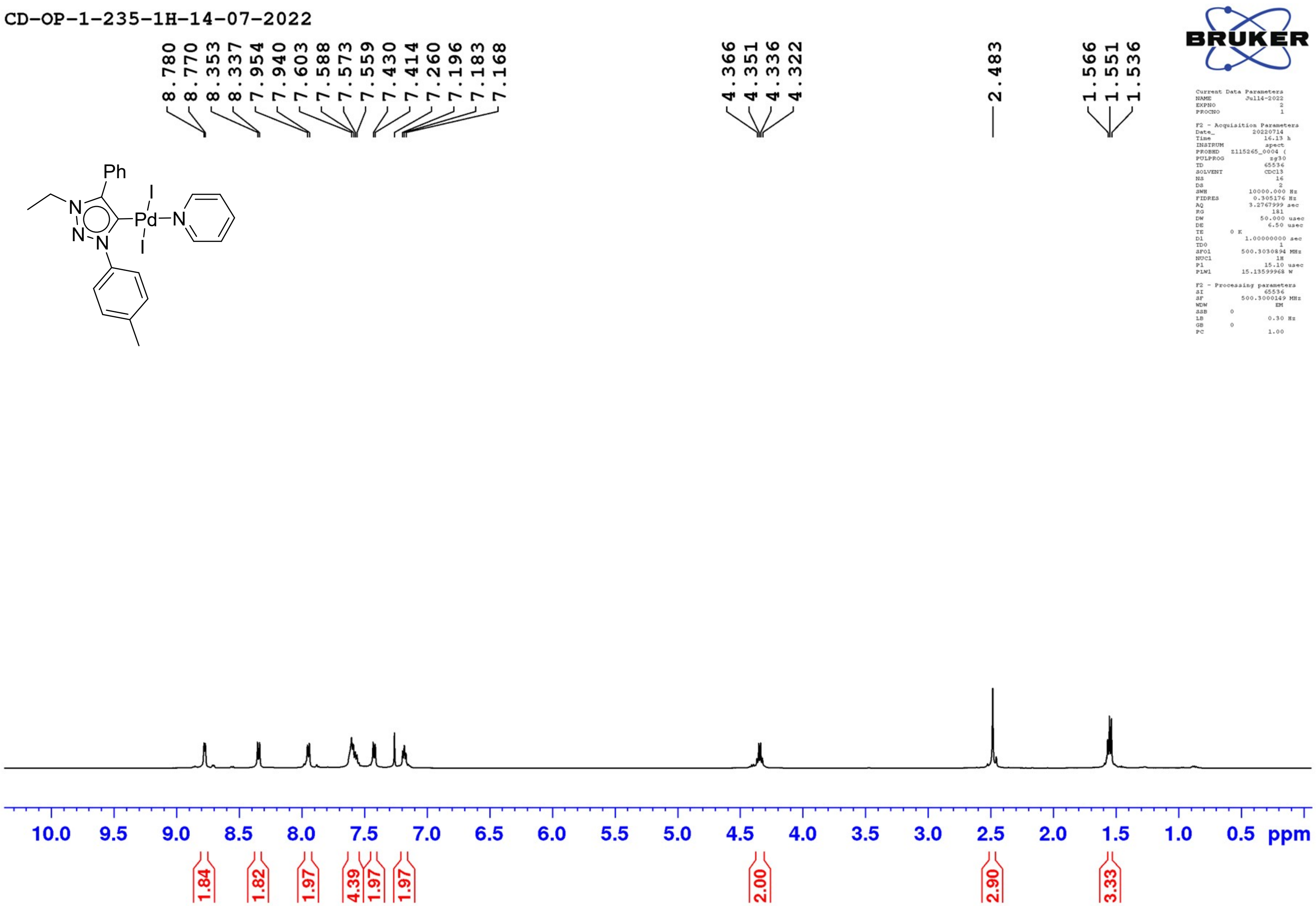

Figure S4. ^{1}H NMR spectrum of **2a** in $CDCl_3$

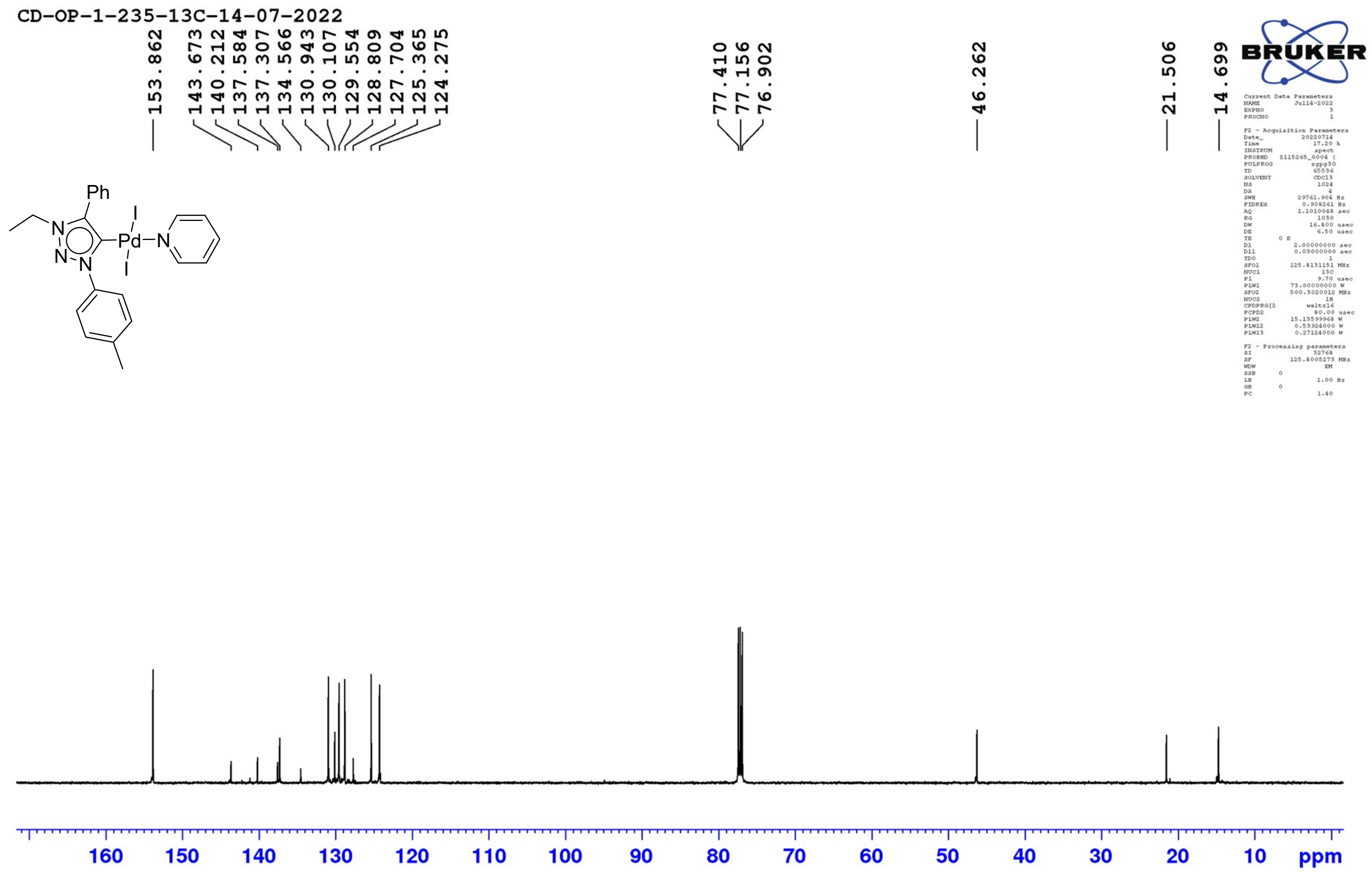

Figure S5. $^{13}C\{^{1}H\}$ NMR spectrum of **2a** in $CDCl_3$

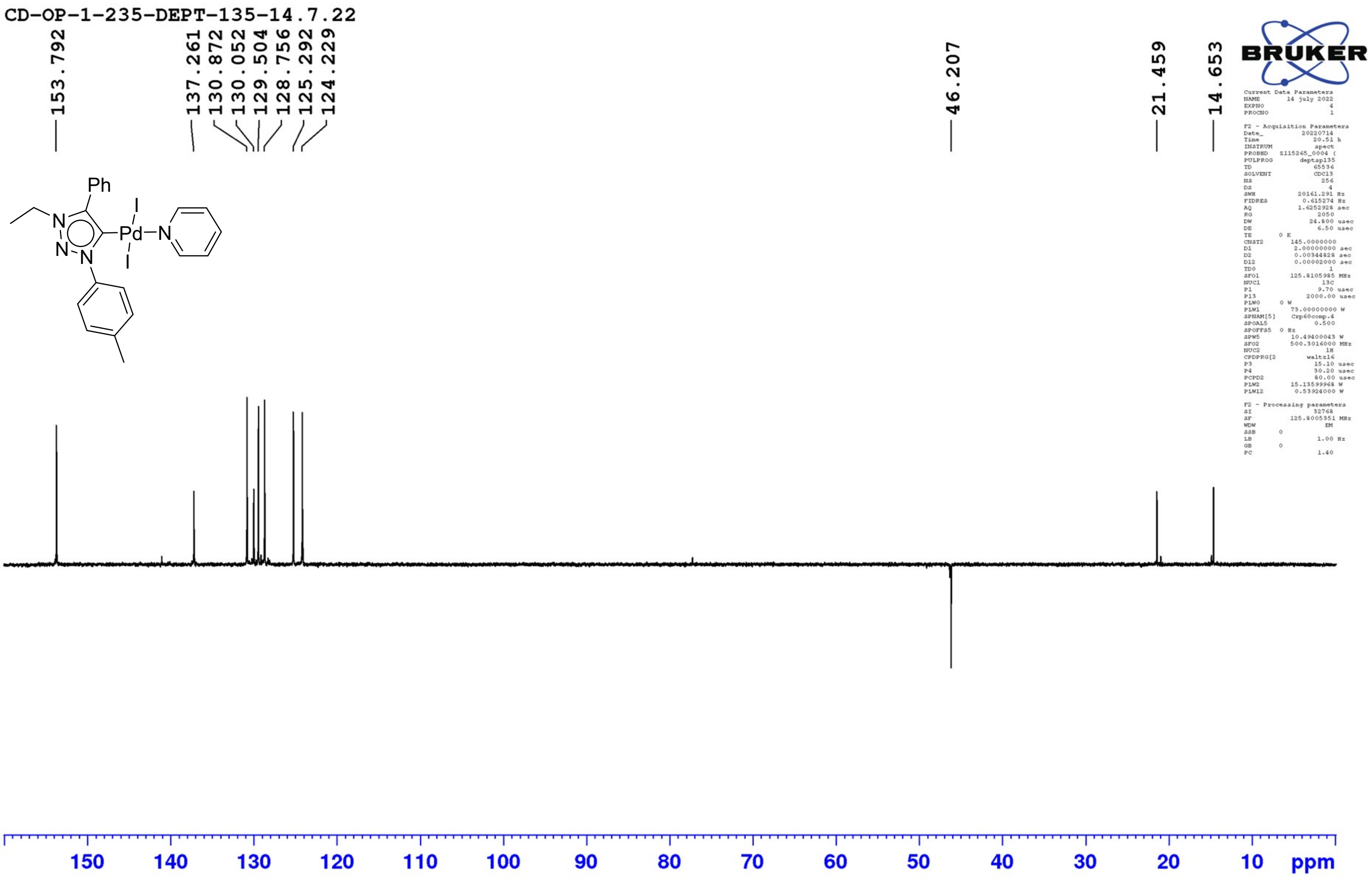

Figure S6. DEPT135 NMR spectrum of **2a** in $CDCl_3$

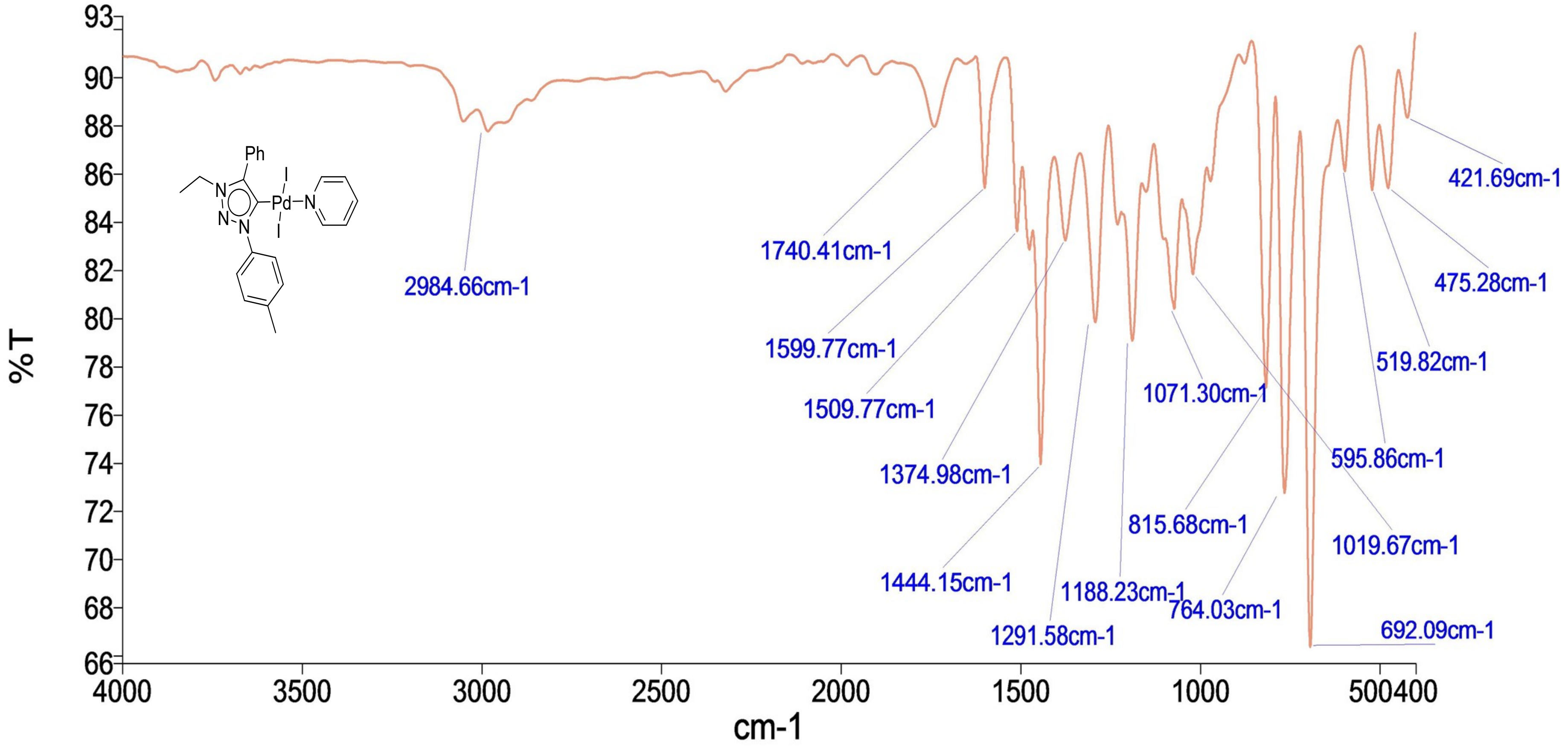

Figure S7. IR spectrum of **2a** in KBr

CHNS Report

Operator ID:	
Company name:	
Method filename:	E:\Method\19-02-2021.mth
Method name:	19-02-2021
Analysed:	02/19/2021 15:47
Printed:	02-19-2021 18:33
Elemental Analyser method:	
Sampler method:	
Sample ID:	CD-OP-1-226 (# 28)
Analysis type:	UnkNown
Chromatogram filename:	CD-OP-1-226-19-2--2021.dat
Calibration method:	K Factors
Sample weight:	2.666
Protein factor:	6.25

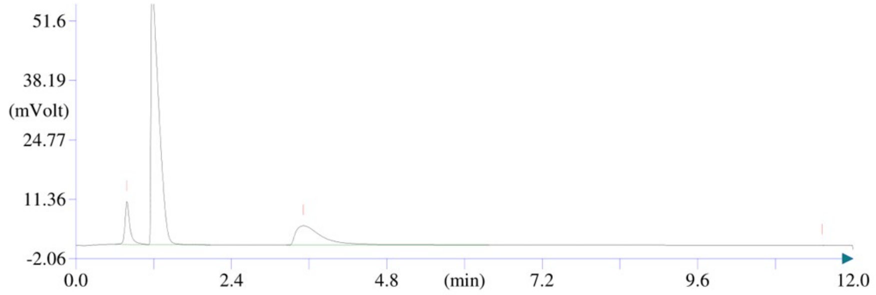

Retention Time (min)	Area (.1*uV*sec)	Component Name	Element %
0.783	505311	Nitrogen	7.428
1.175	4919907	Carbon	37.591
3.508	1256201	Hydrogen	3.123
11.525	9913		0.000
	6691331		48.142

Figure S8. Elemental Analysis data of **2a**

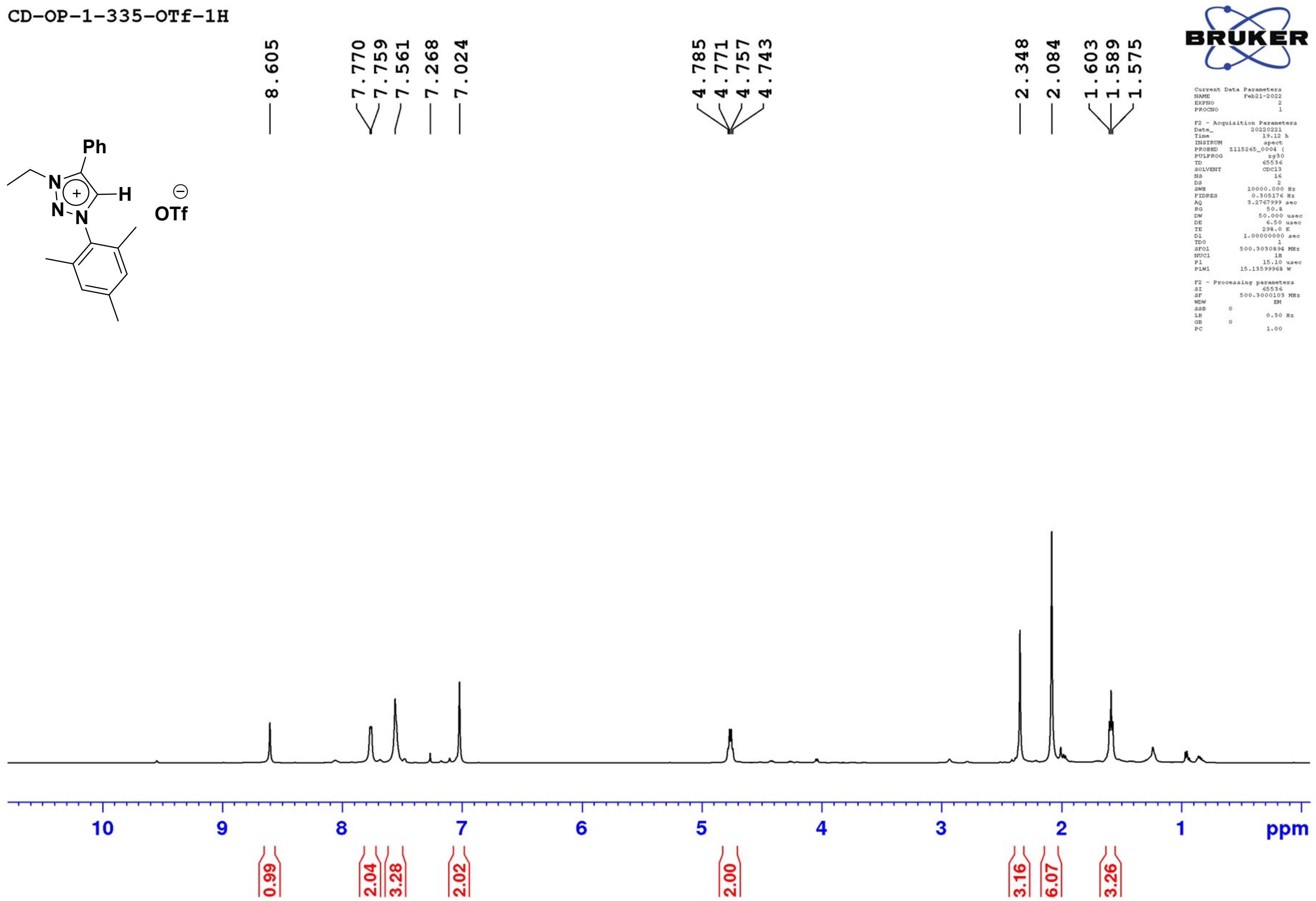

Figure S9. ^{1}H NMR spectrum of **1b** in $CDCl_3$

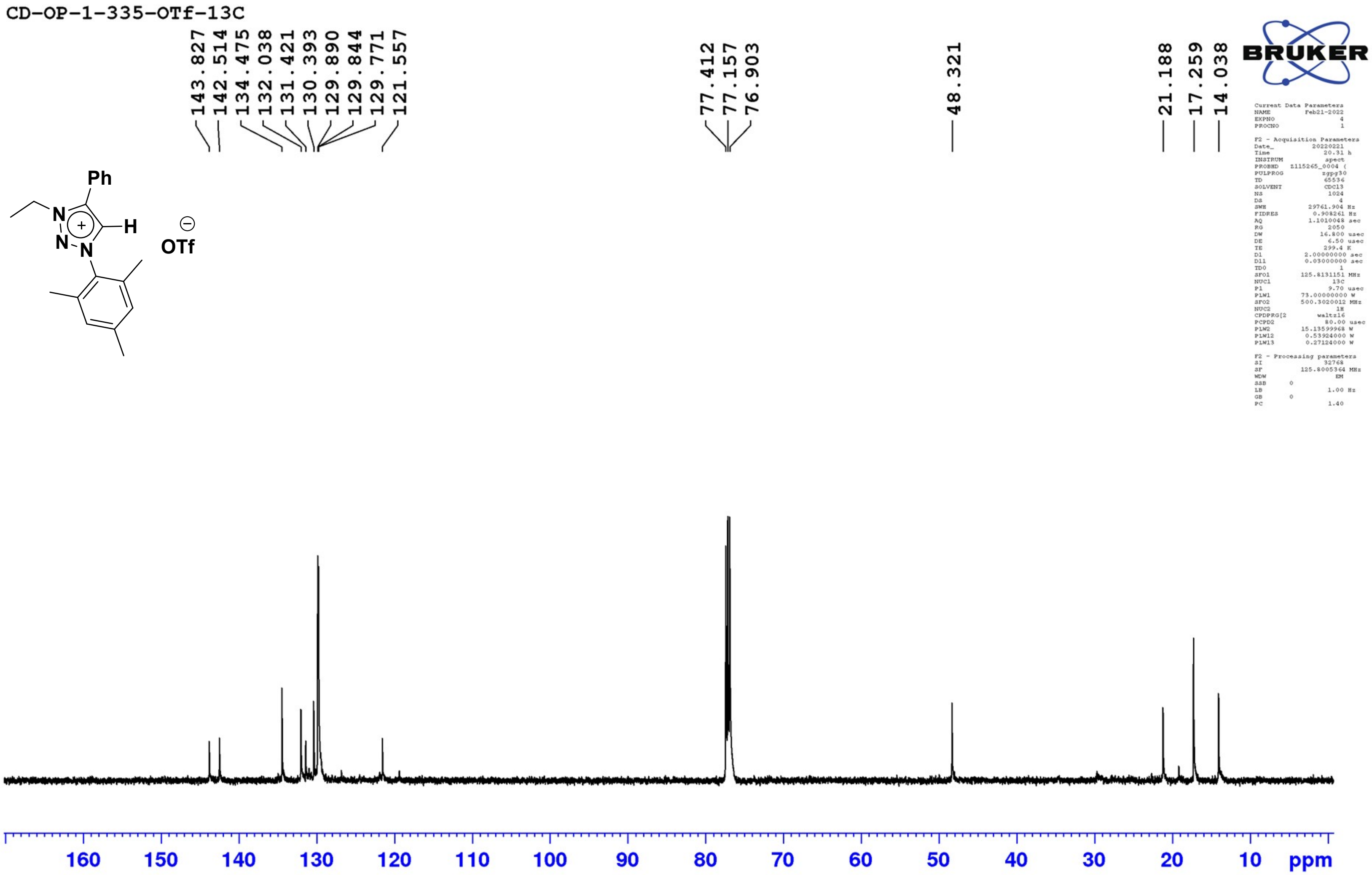

Figure S10. $^{13}C\{^{1}H\}$ NMR spectrum of **1b** in $CDCl_3$

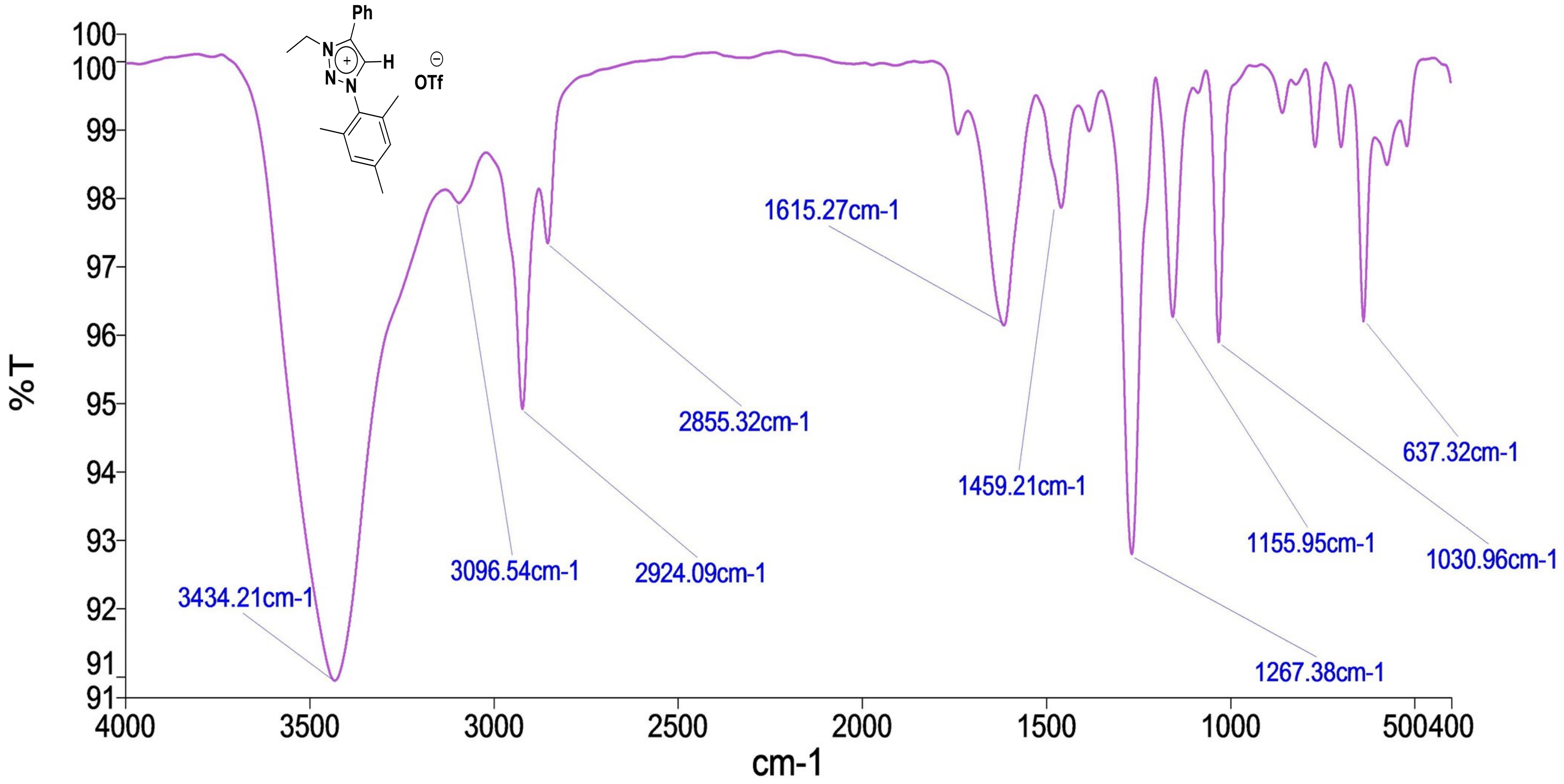

Figure S11. IR spectrum of **1b** in KBr

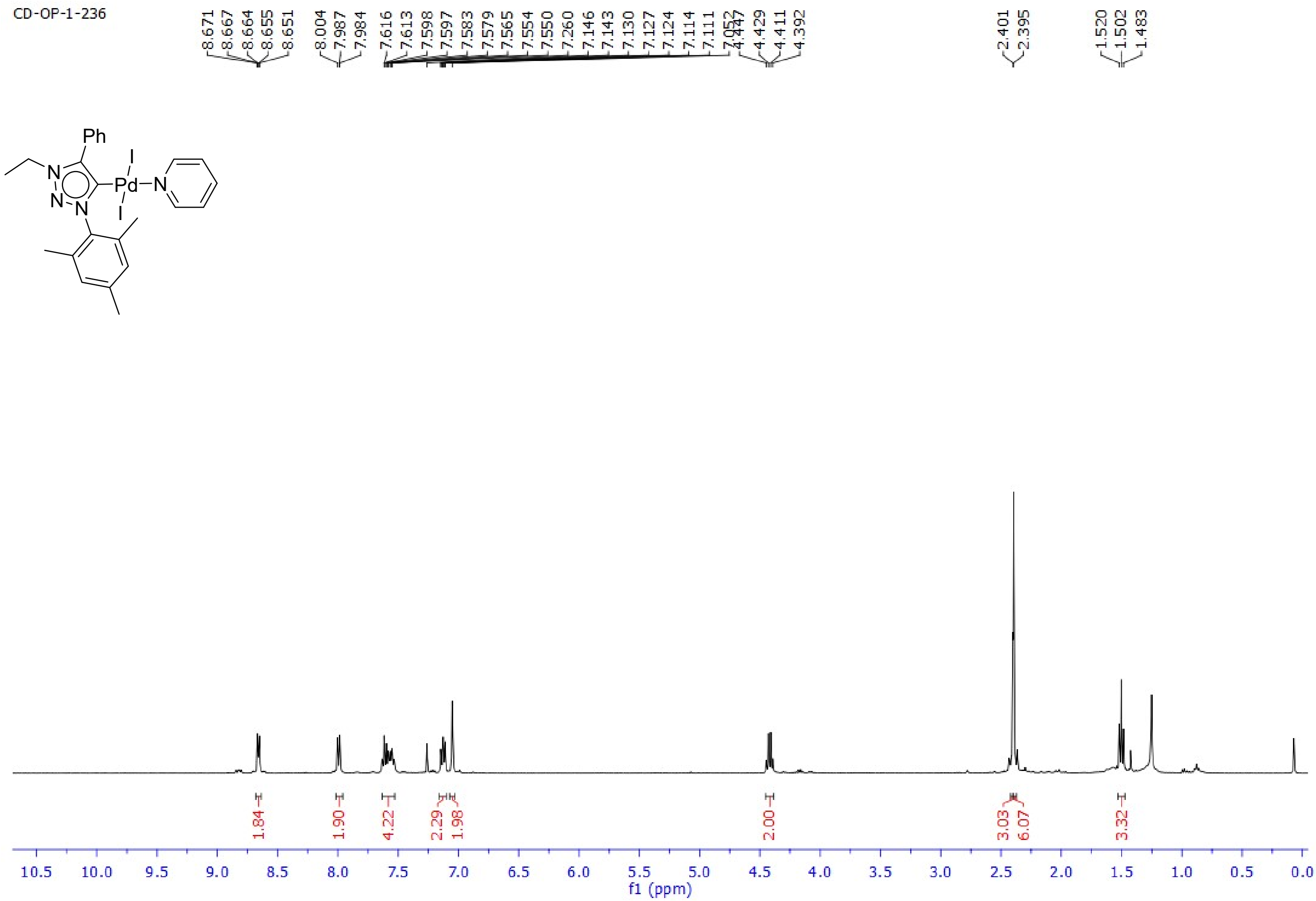

Figure S12. ^{1}H NMR spectrum of **2b** in $CDCl_3$

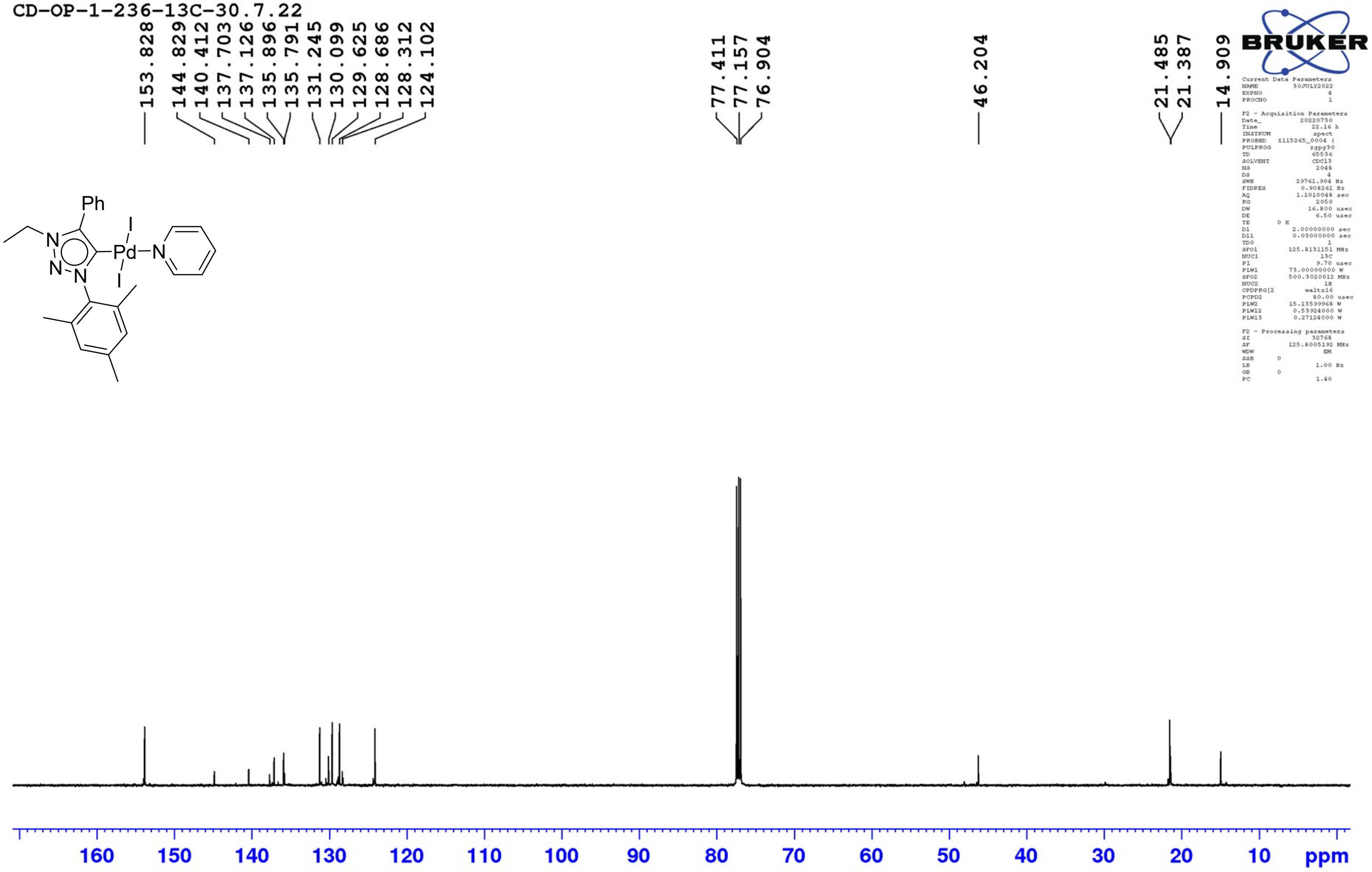

Figure S13. $^{13}C\{^{1}H\}$ NMR spectrum of **2b** in $CDCl_3$

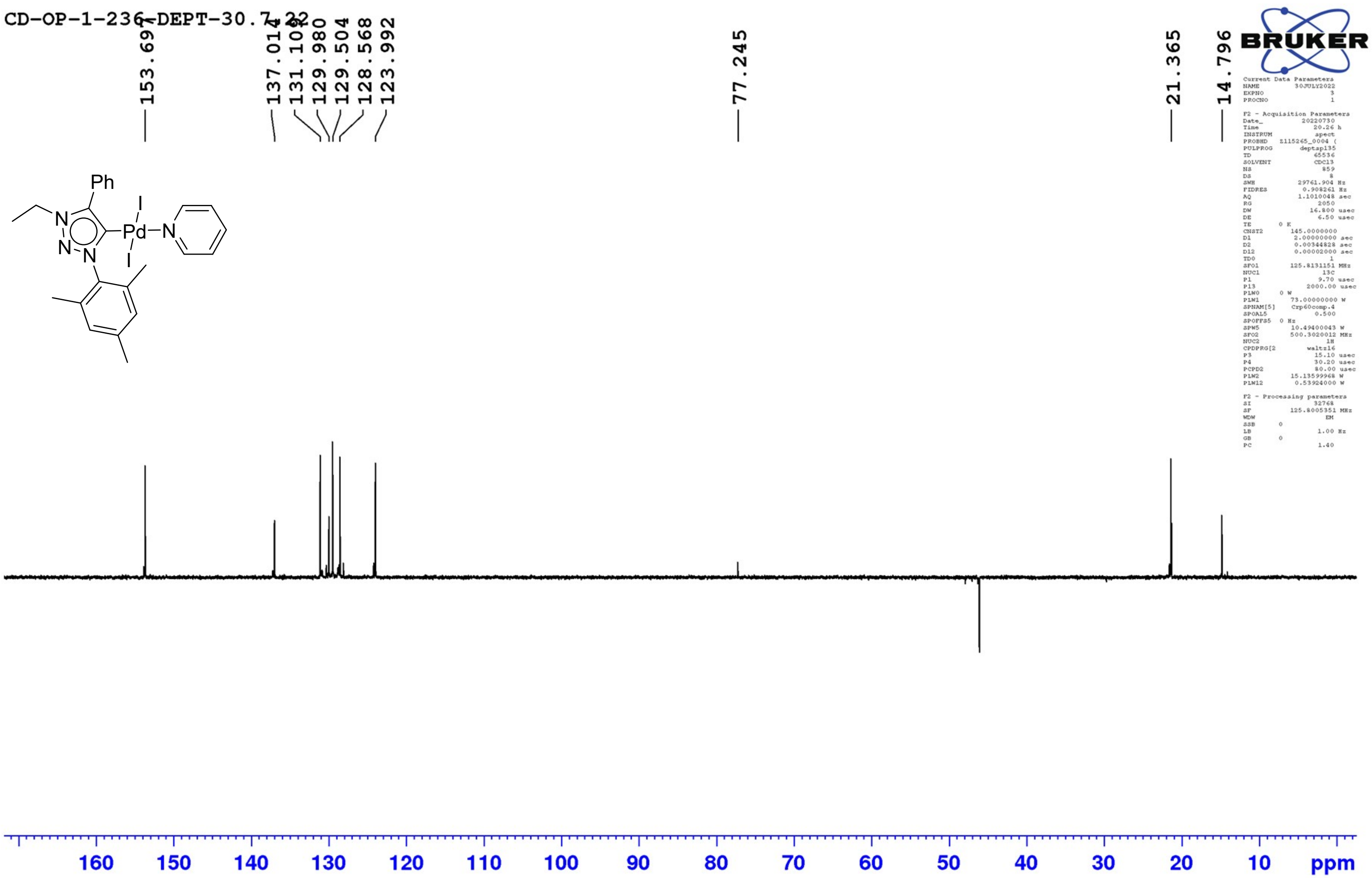

Figure S14. DEPT135 NMR spectrum of **2b** in $CDCl_3$

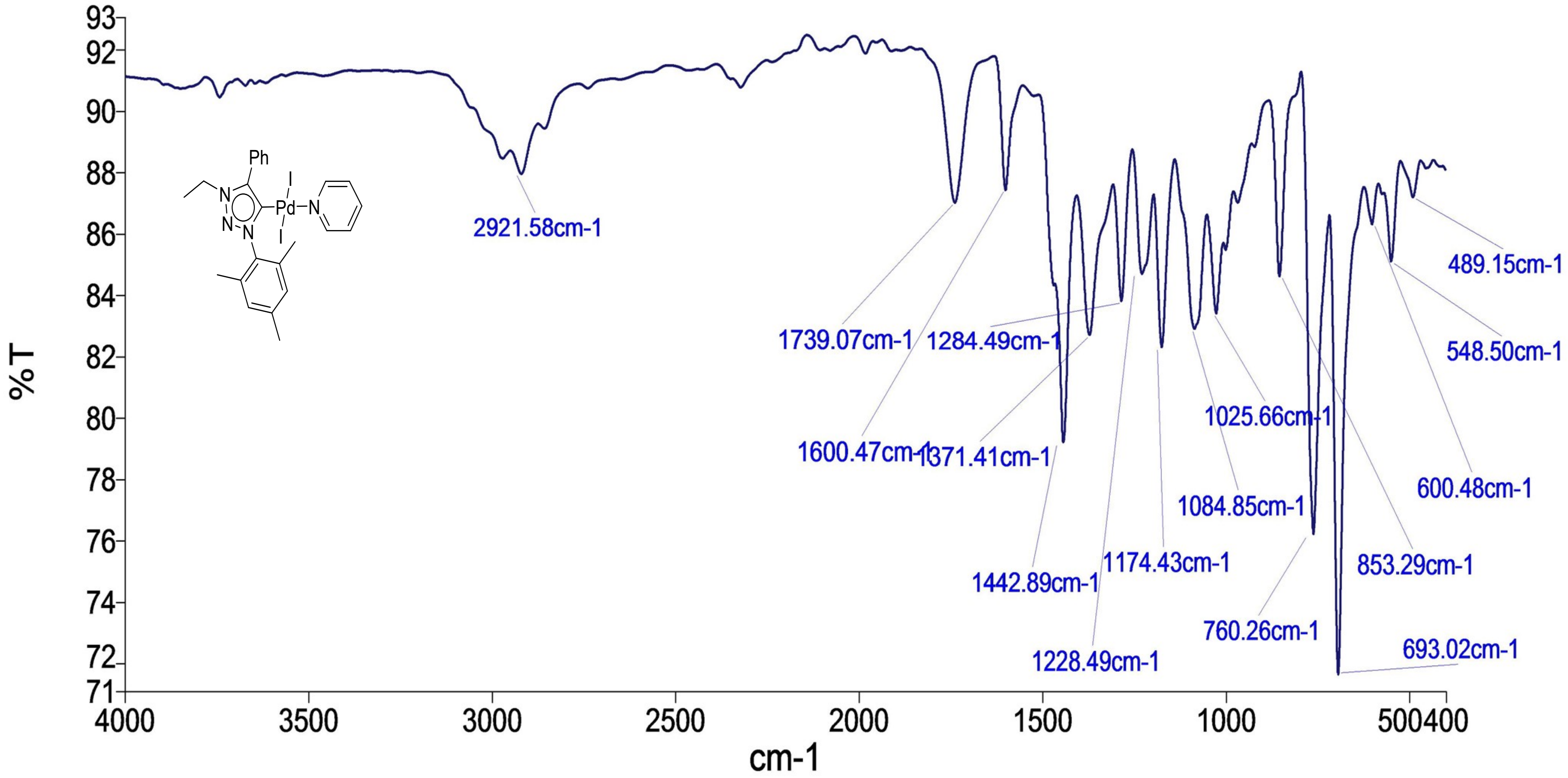

Figure S15. IR spectrum of **2b** in KBr

CHNS Report

Operator ID:	
Company name:	
Method filename:	E:\Method\19-02-2021.mth
Method name:	19-02-2021
Analysed:	02/19/2021 15:23
Printed:	02-19-2021 18:32
Elemental Analyser method:	
Sampler method:	
Sample ID:	CD-OP-1-215 (# 26)
Analysis type:	UnkNown
Chromatogram filename:	CD-OP-1-215-19-02-2021.dat
Calibration method:	K Factors
Sample weight:	3.175
Protein factor:	6.25

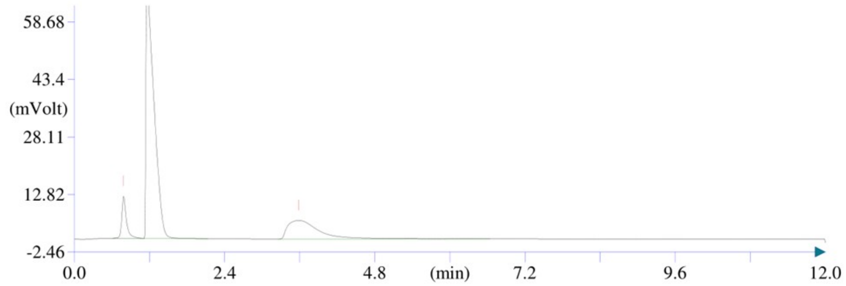

Retention Time (min)	Area (.1*uV*sec)	Component Name	Element %
0.783	567889	Nitrogen	7.208
1.158	6062293	Carbon	38.901
3.583	1704203	Hydrogen	3.563
	8334385		49.672

Figure S16. Elemental Analysis data of **2b**

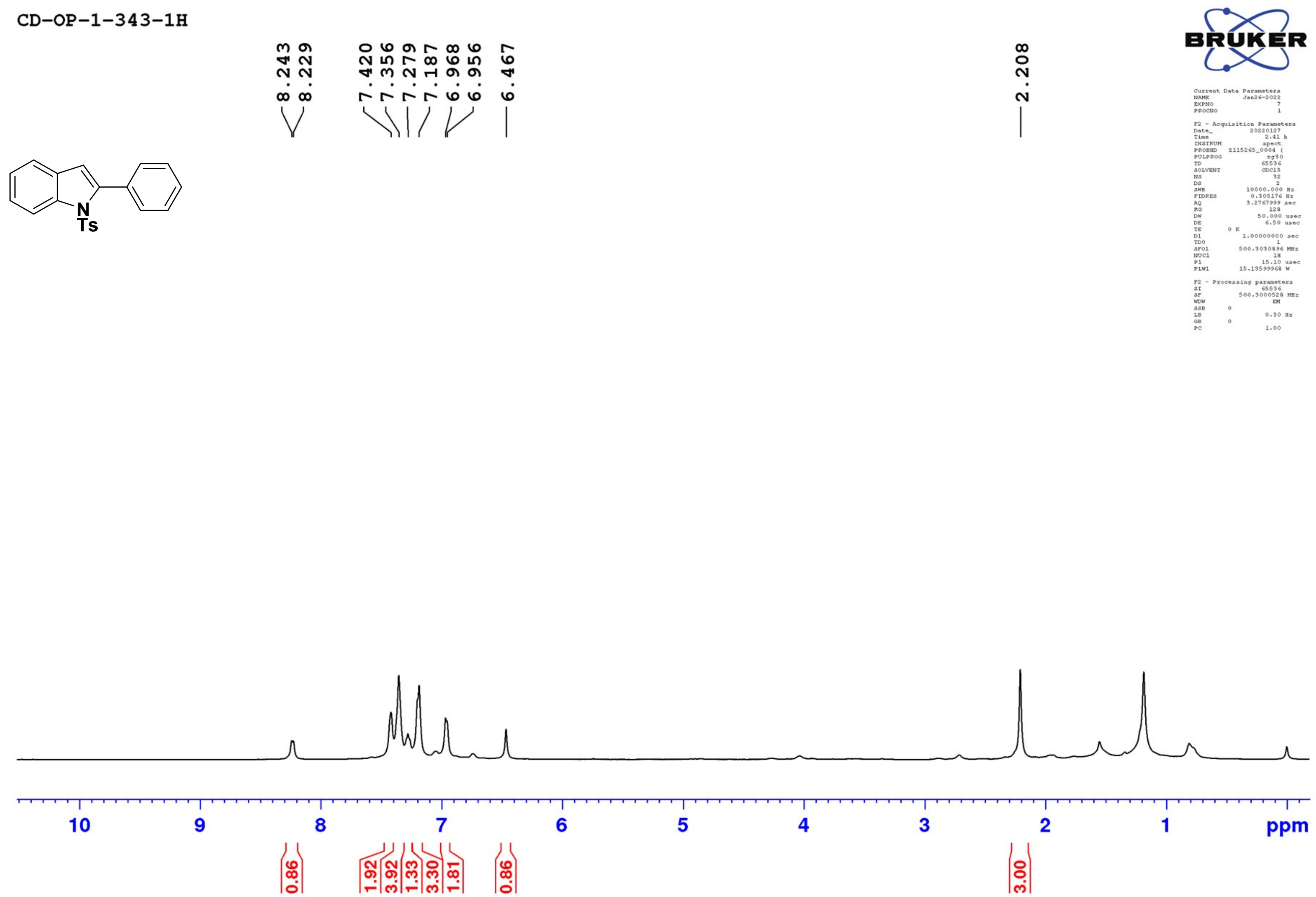

Figure S17. ^{1}H NMR spectrum of **3ah** in $CDCl_3$

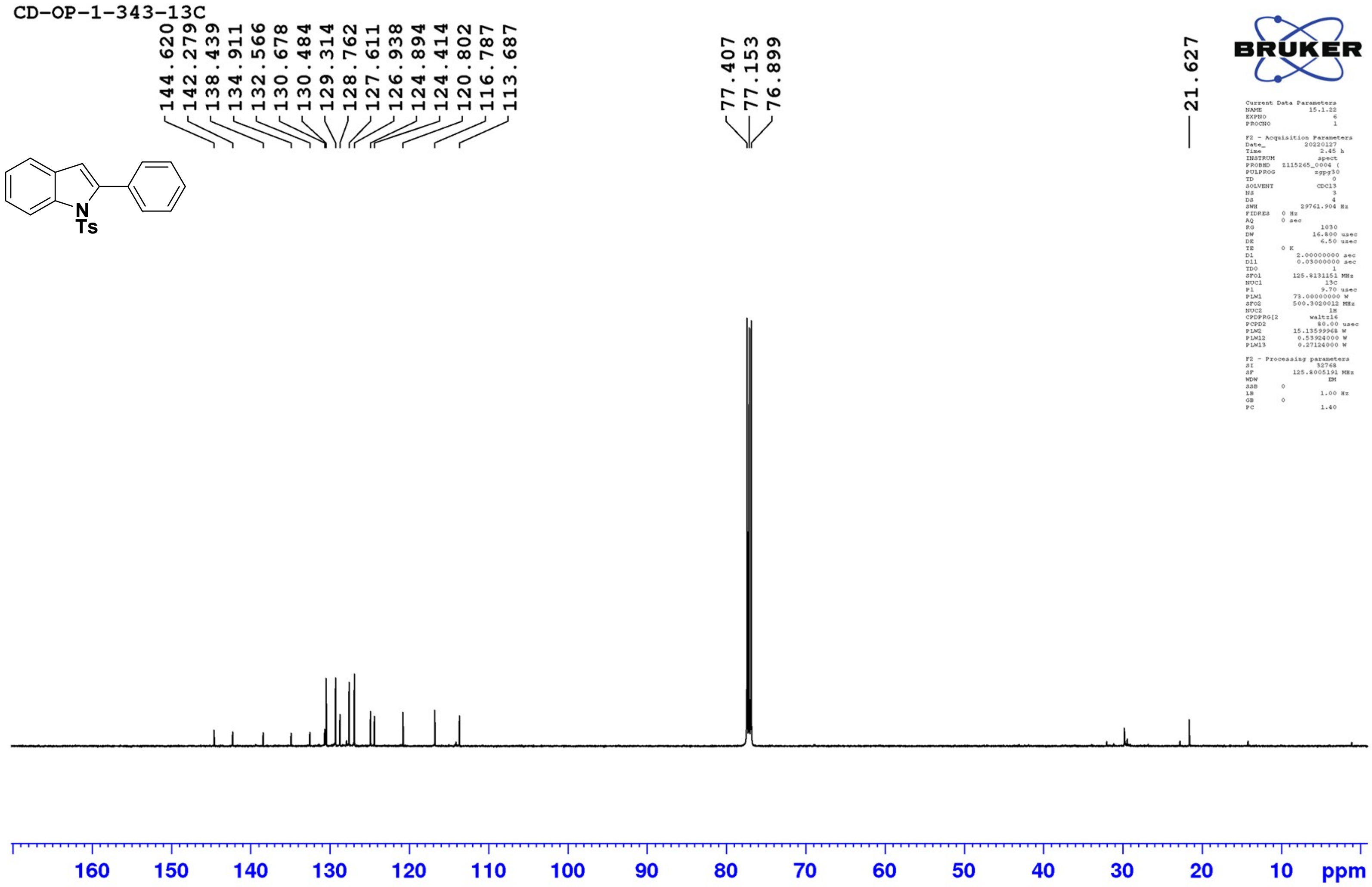

Figure S18. $^{13}C\{^{1}H\}$ NMR spectrum of **3ah** in $CDCl_3$

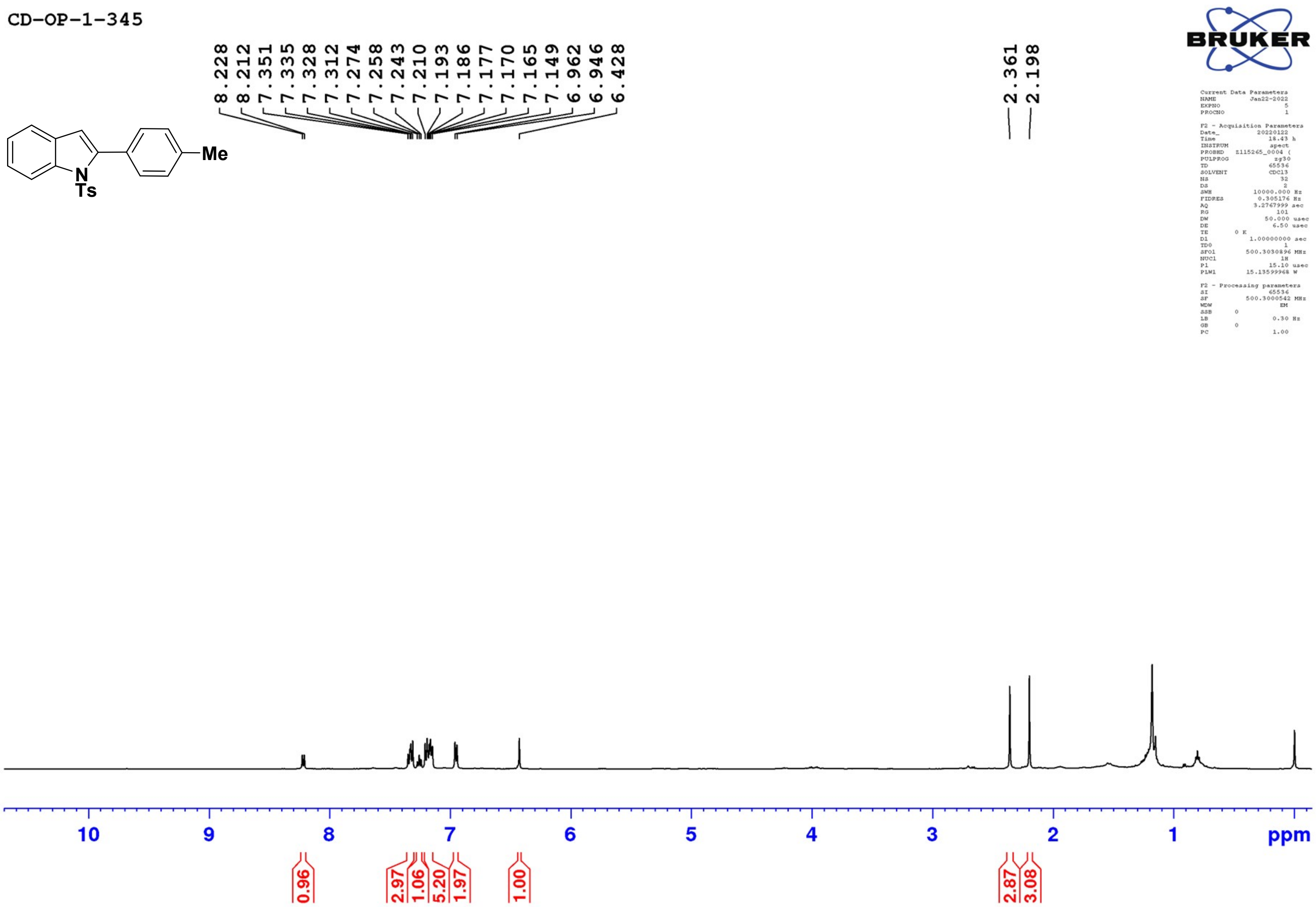

Figure S19. ^{1}H NMR spectrum of **3ai** in $CDCl_3$

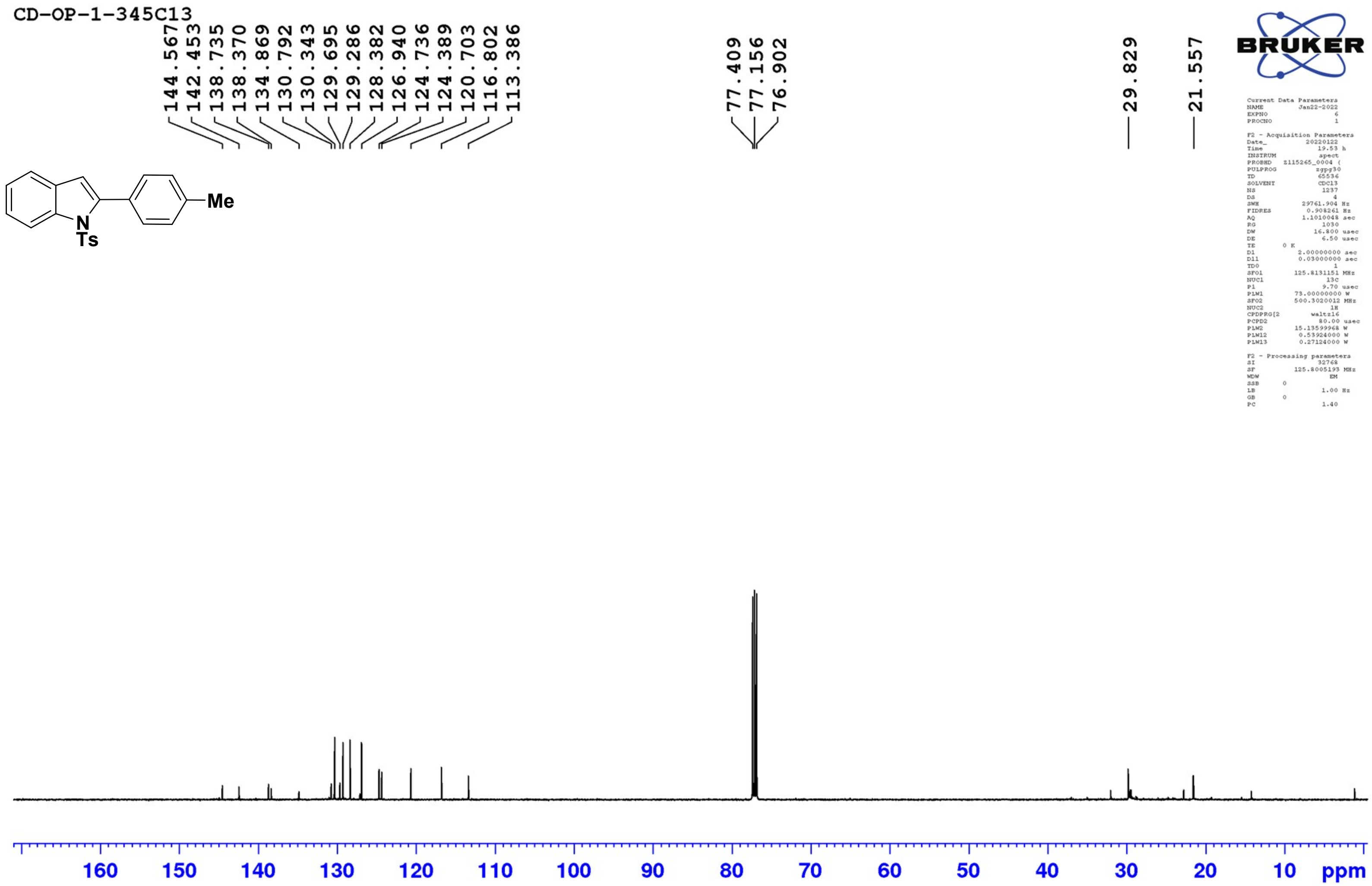

Figure S20. $^{13}C\{^{1}H\}$ NMR spectrum of **3ai** in $CDCl_3$

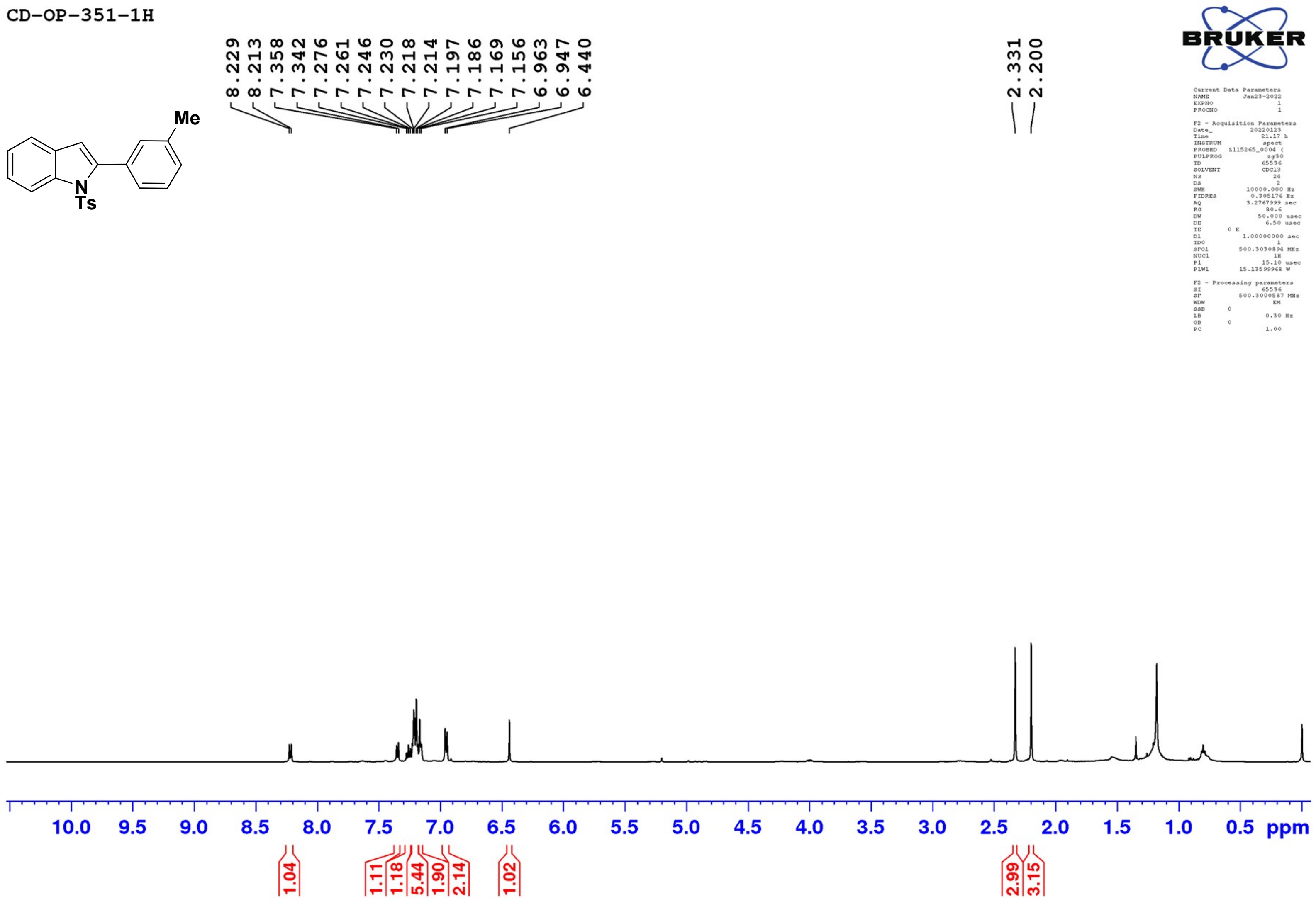

Figure S21. ^{1}H NMR spectrum of **3aj** in $CDCl_3$

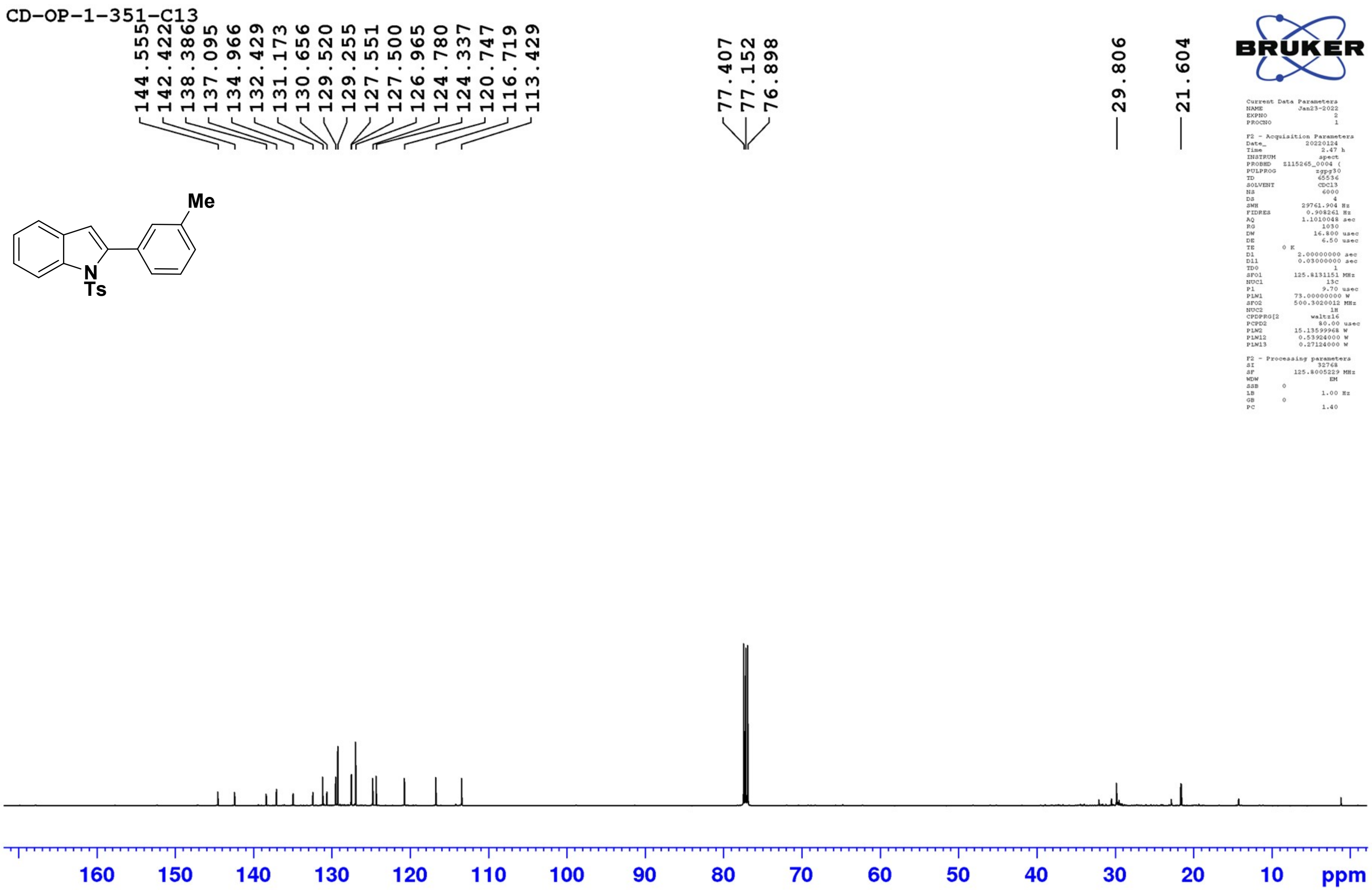

Figure S22. $^{13}C\{^{1}H\}$ NMR spectrum of **3aj** in $CDCl_3$

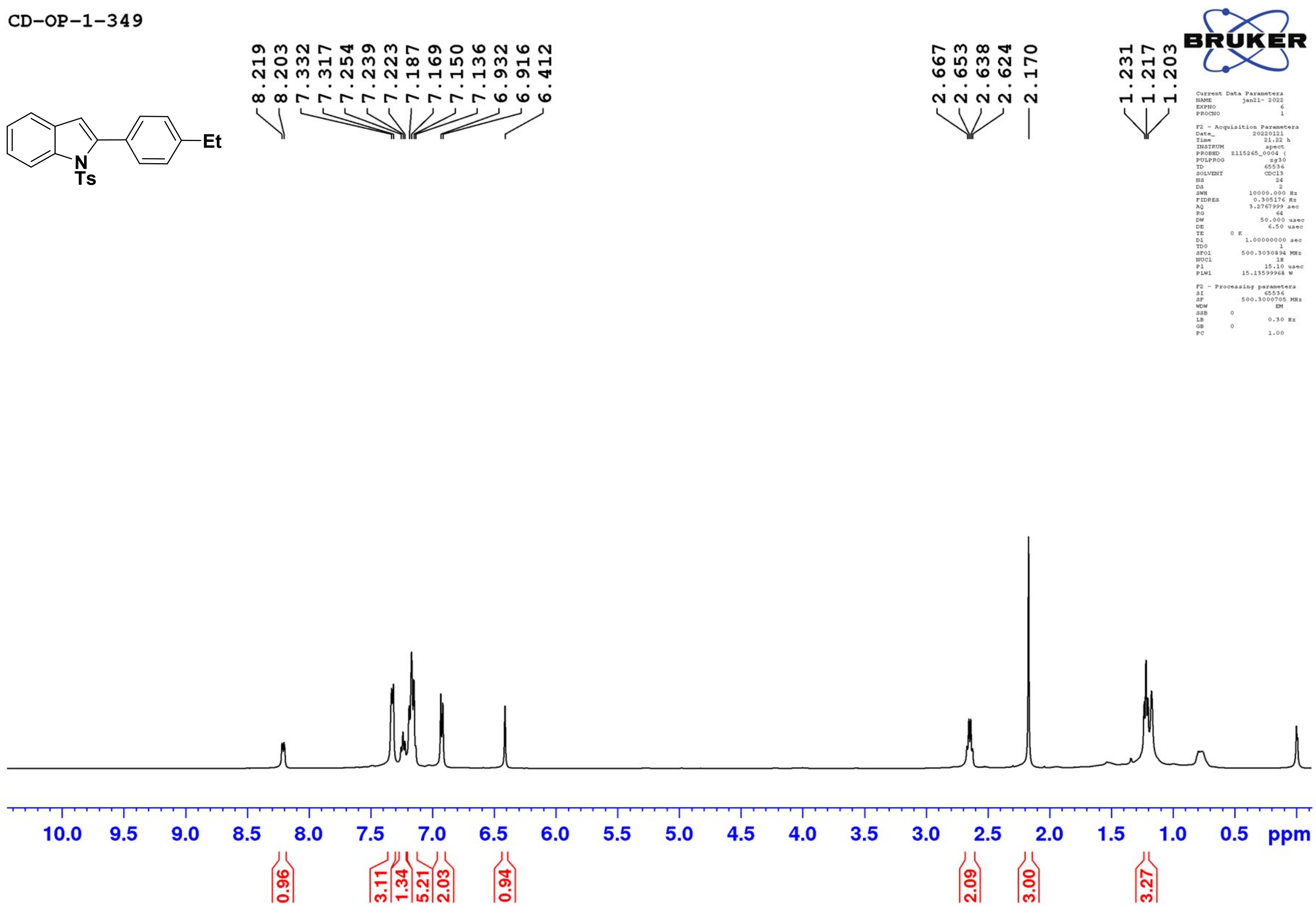

Figure S23. ^{1}H NMR spectrum of **3ak** in $CDCl_3$

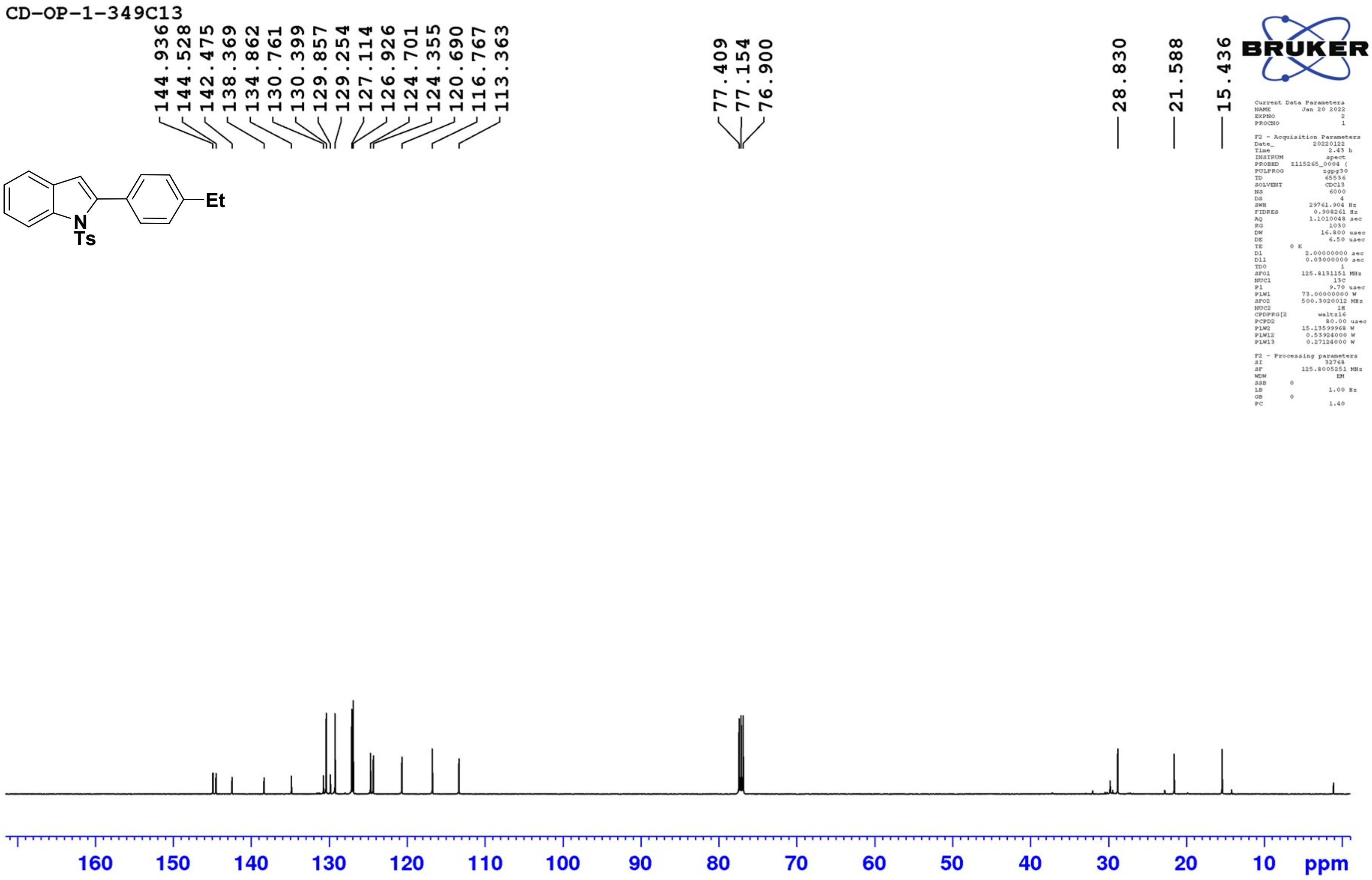

Figure S24. $^{13}C\{^{1}H\}$ NMR spectrum of **3ak** in $CDCl_3$

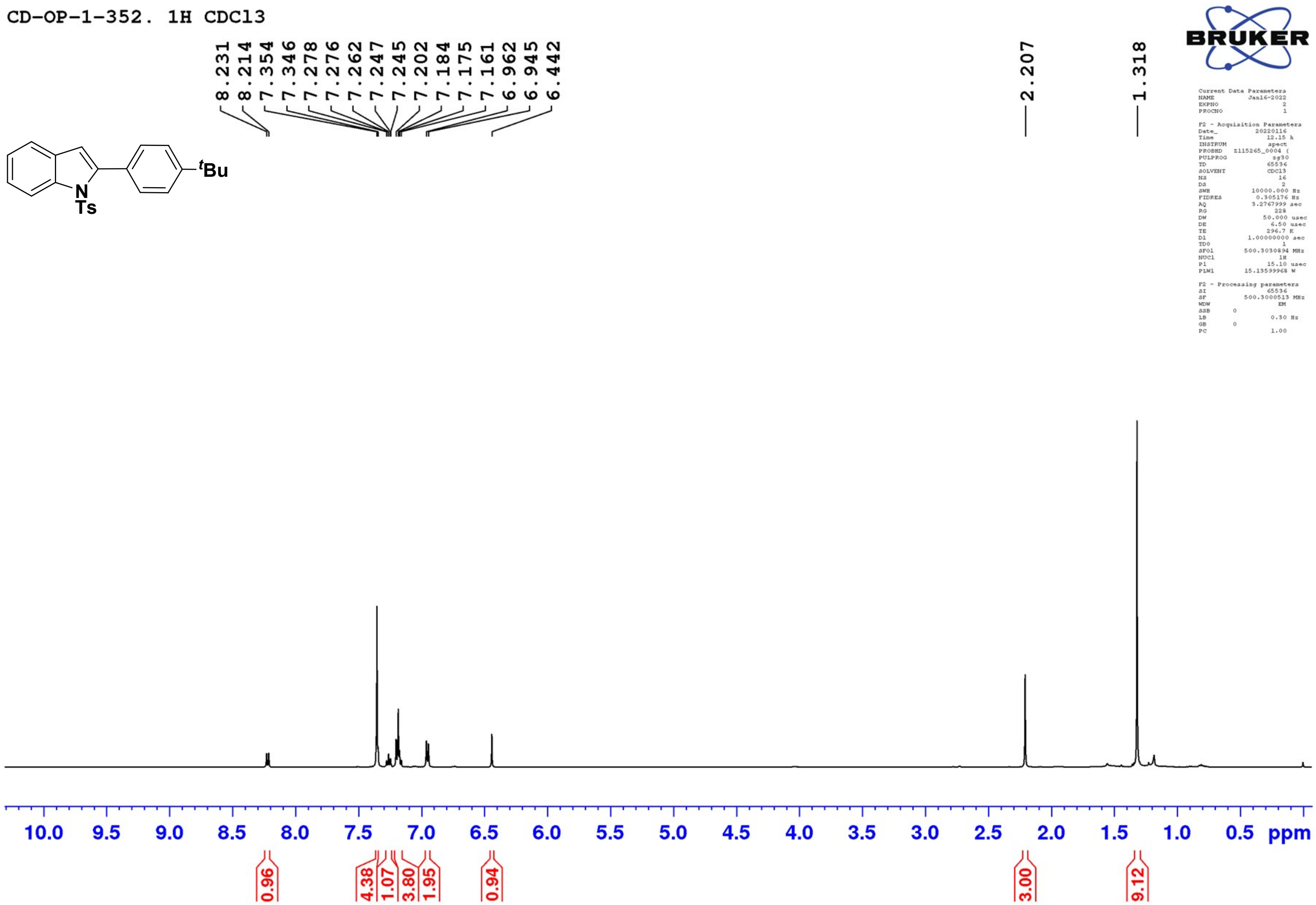

Figure S25. ^{1}H NMR spectrum of **3al** in $CDCl_3$

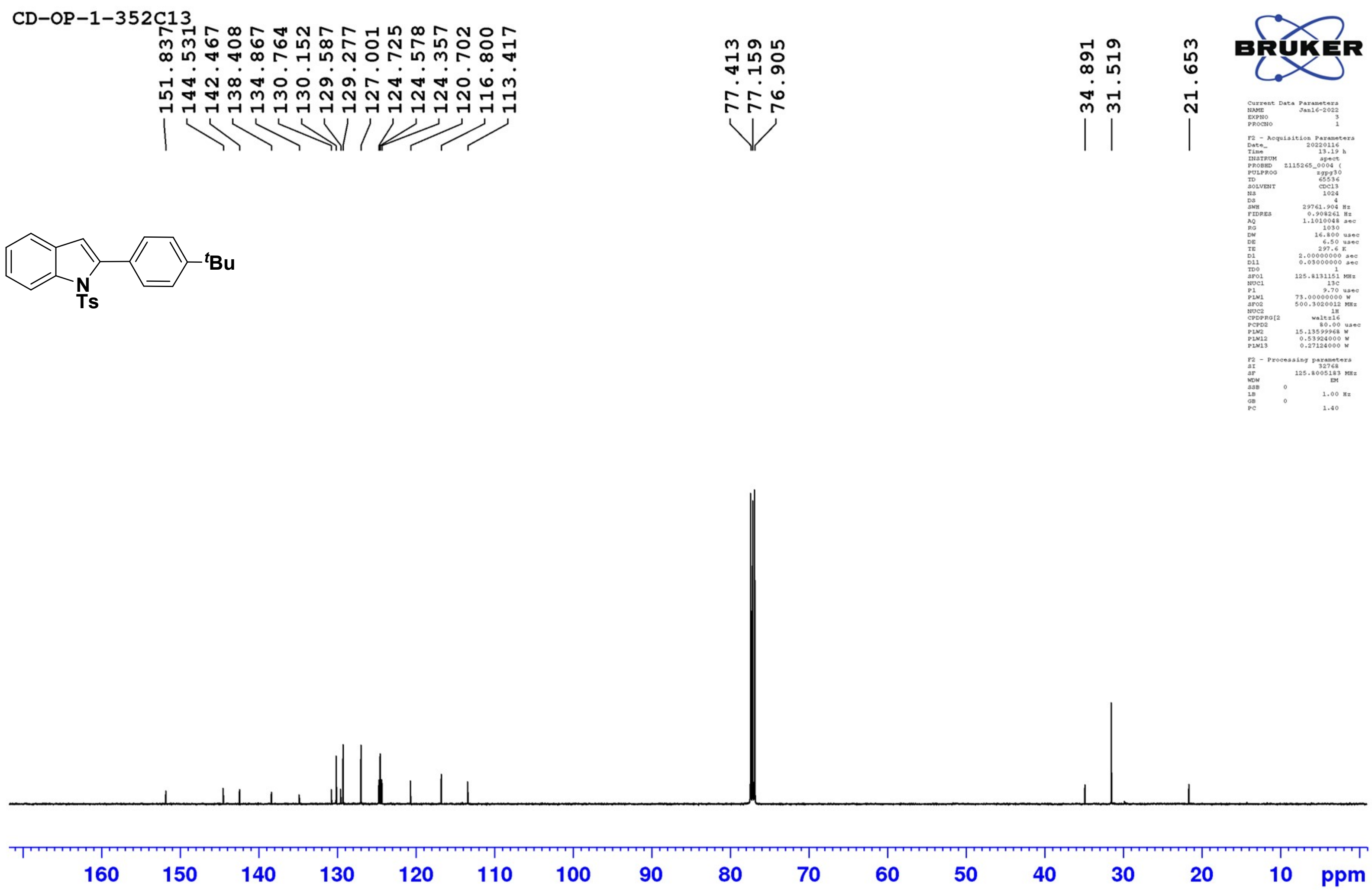

Figure S26. $^{13}C\{^1H\}$ NMR spectrum of **3al** in $CDCl_3$

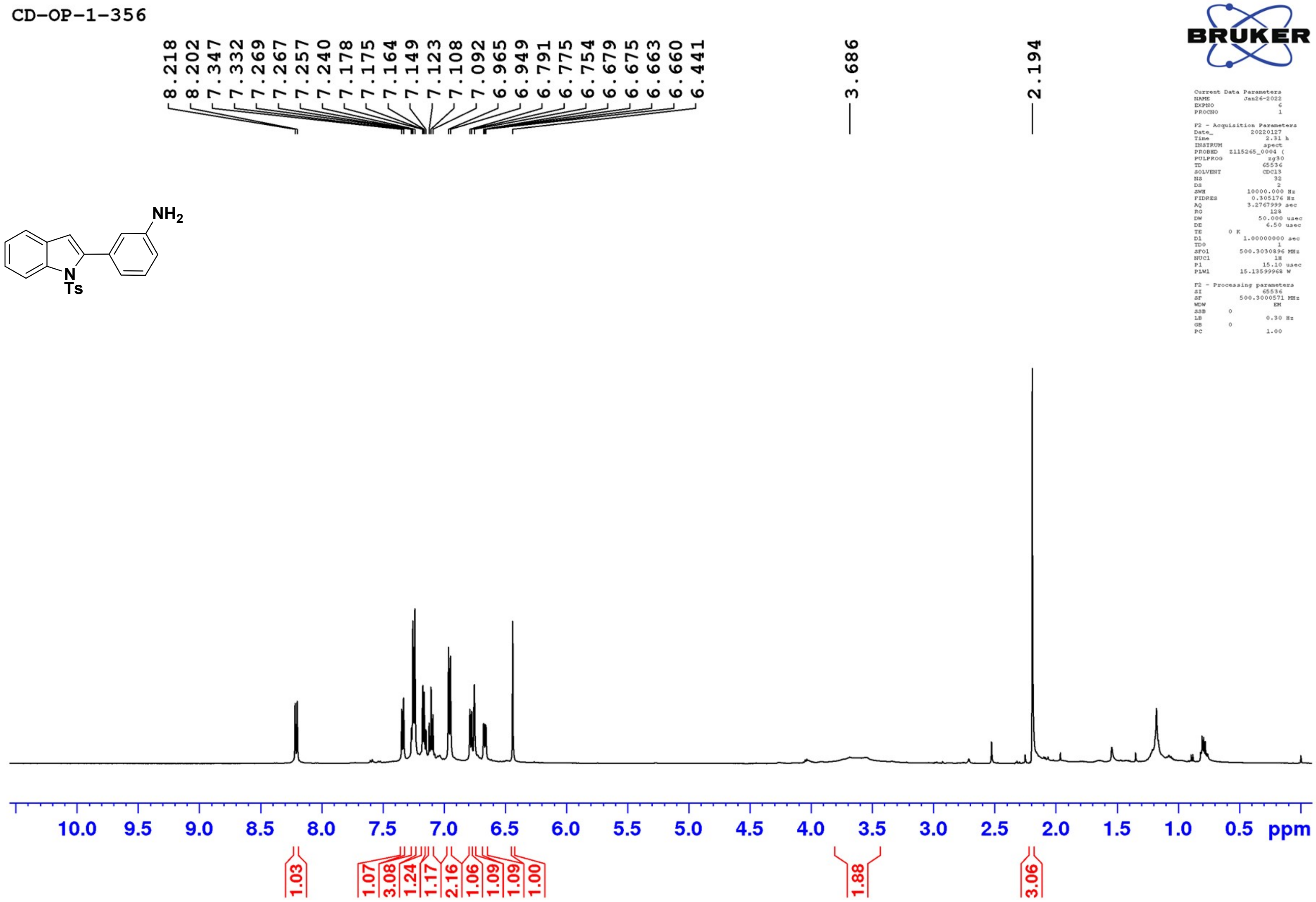

Figure S27. ^{1}H NMR spectrum of **3am** in $CDCl_3$

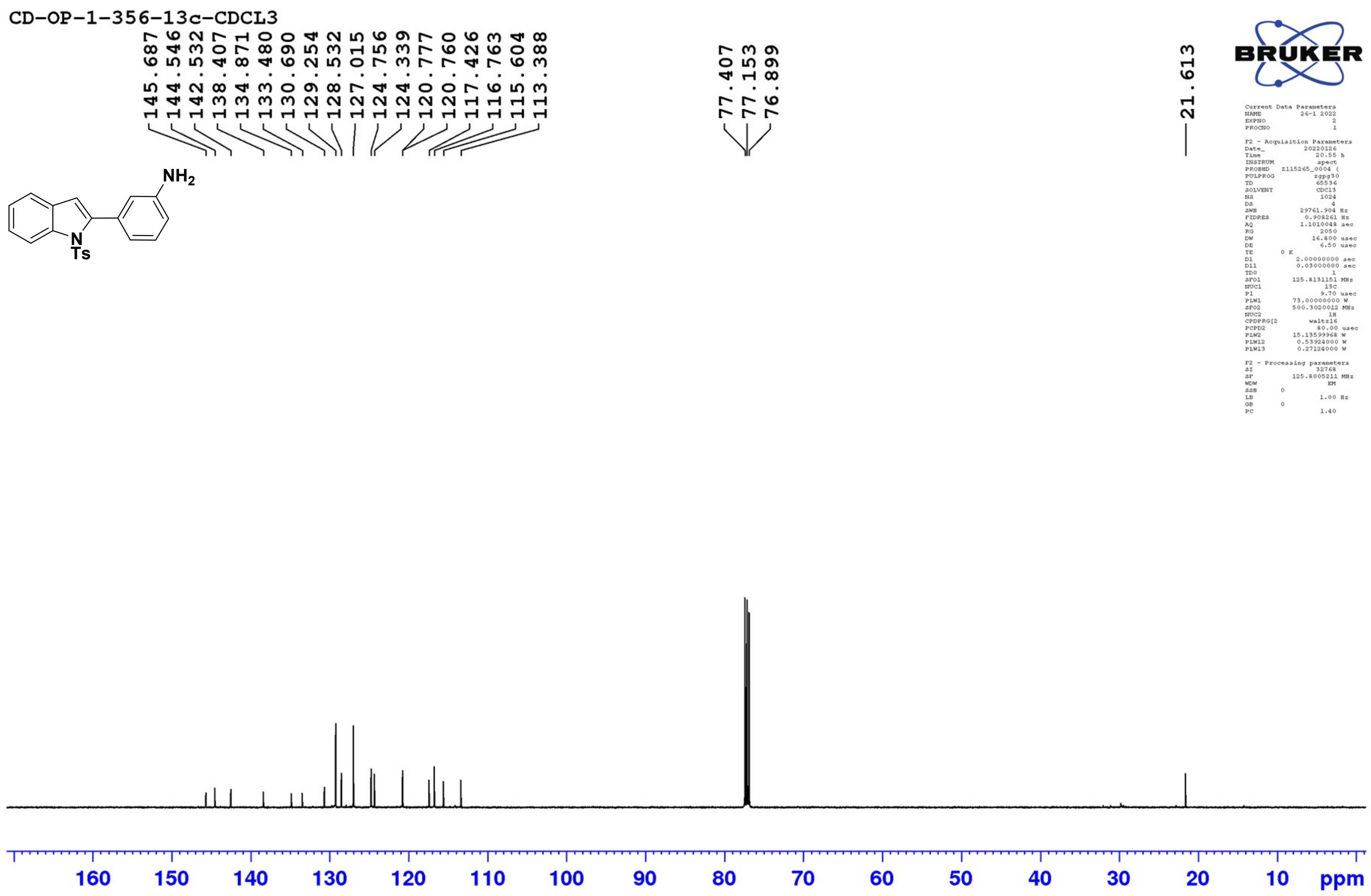

Figure S28. $^{13}C\{^{1}H\}$ NMR spectrum of **3am** in $CDCl_3$

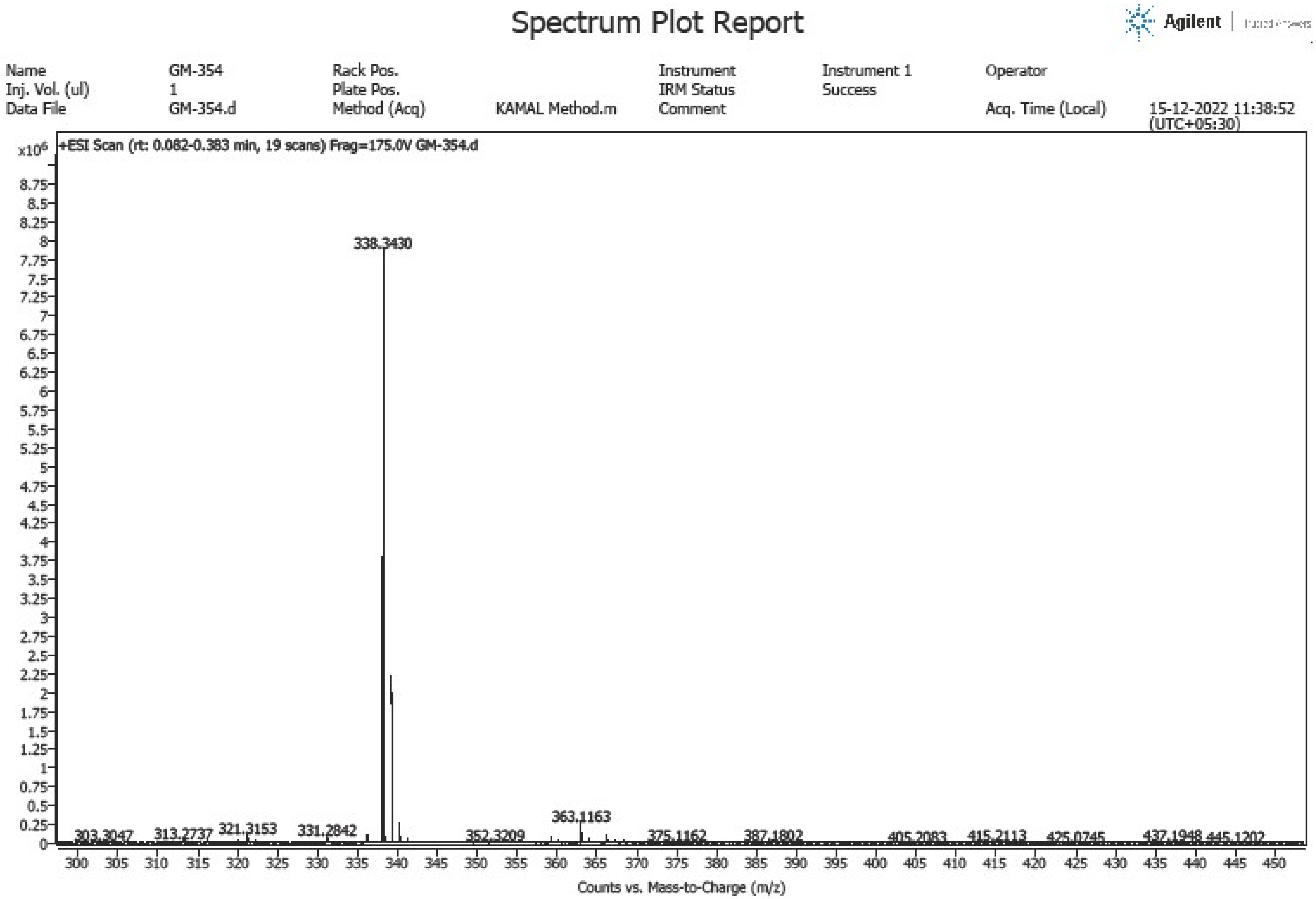

Figure S29. High Resolution Mass Spectrometry (HRMS) data of **3am**

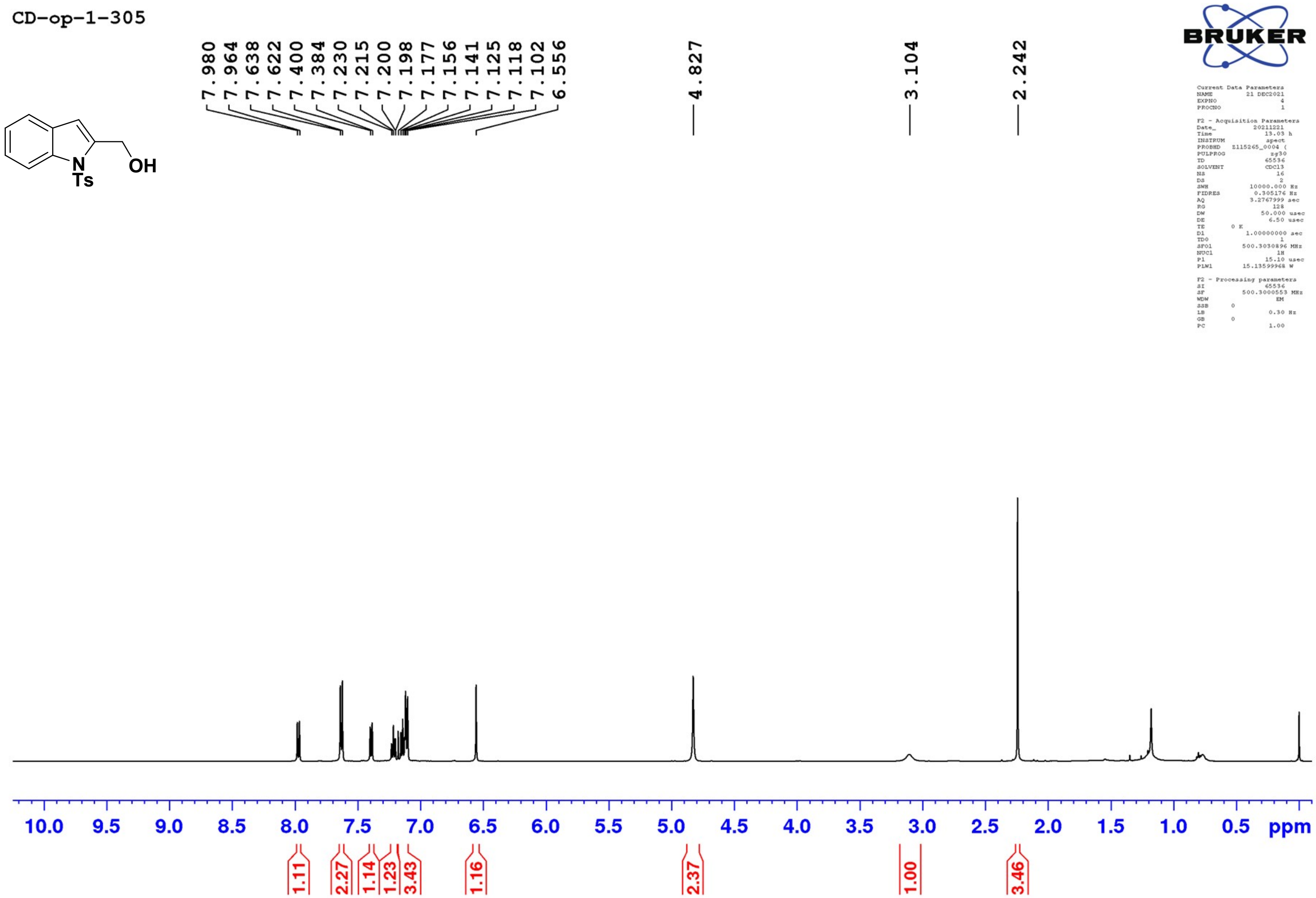

Figure S30. ^{1}H NMR spectrum of **3an** in $CDCl_3$

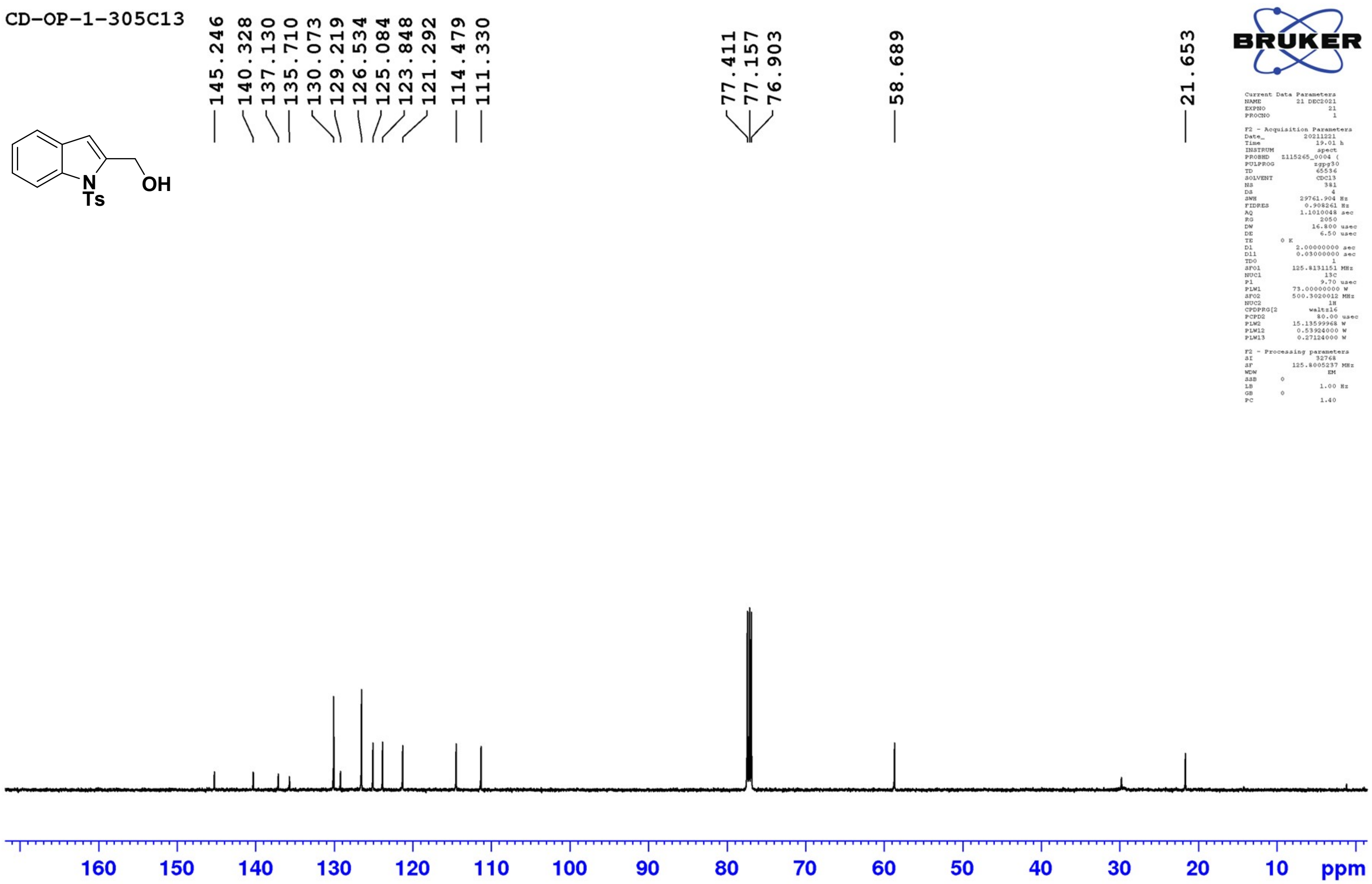

Figure S31. $^{13}C\{^{1}H\}$ NMR spectrum of **3an** in $CDCl_3$

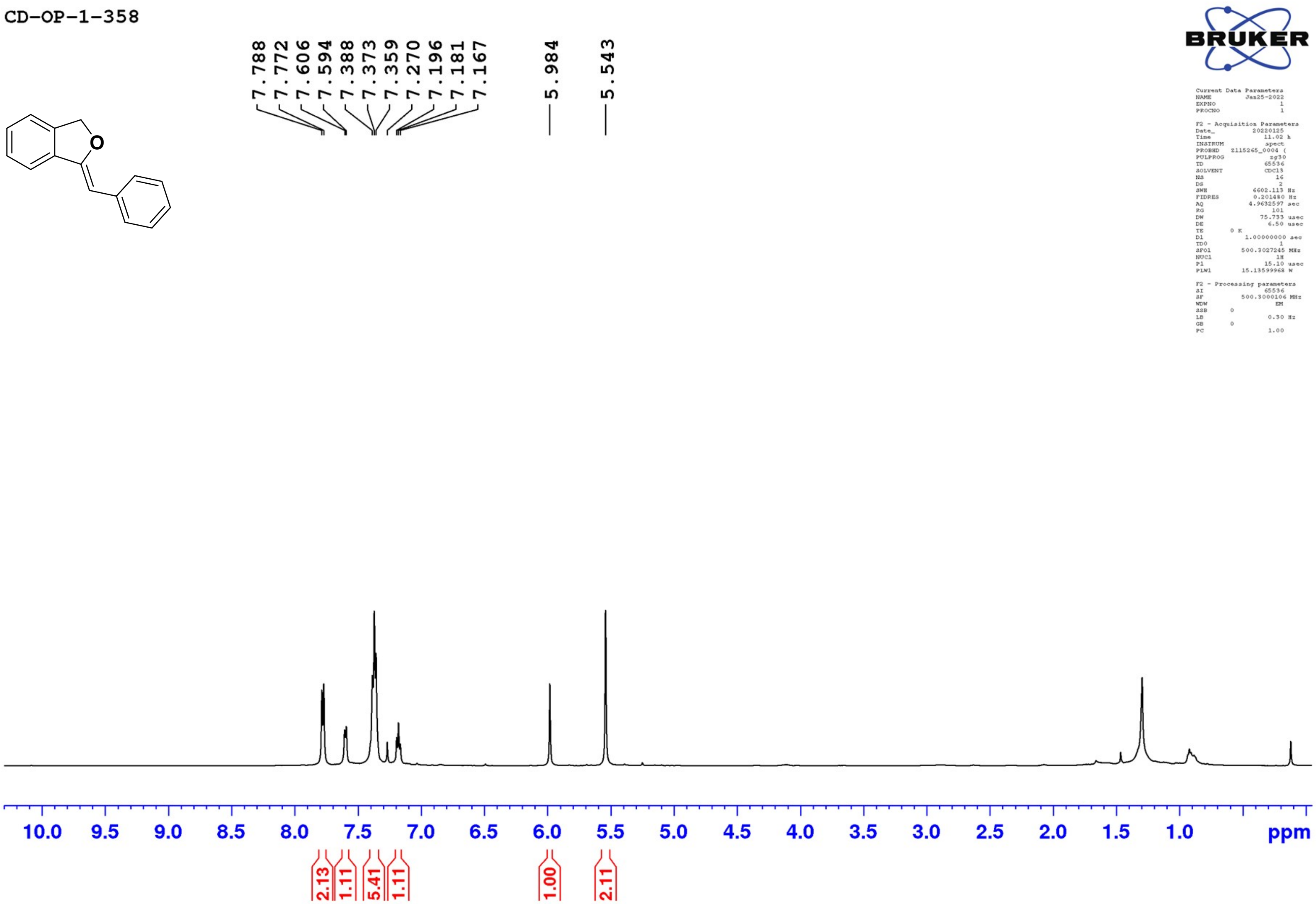

Figure S32. ^{1}H NMR spectrum of **4aa** in $CDCl_3$

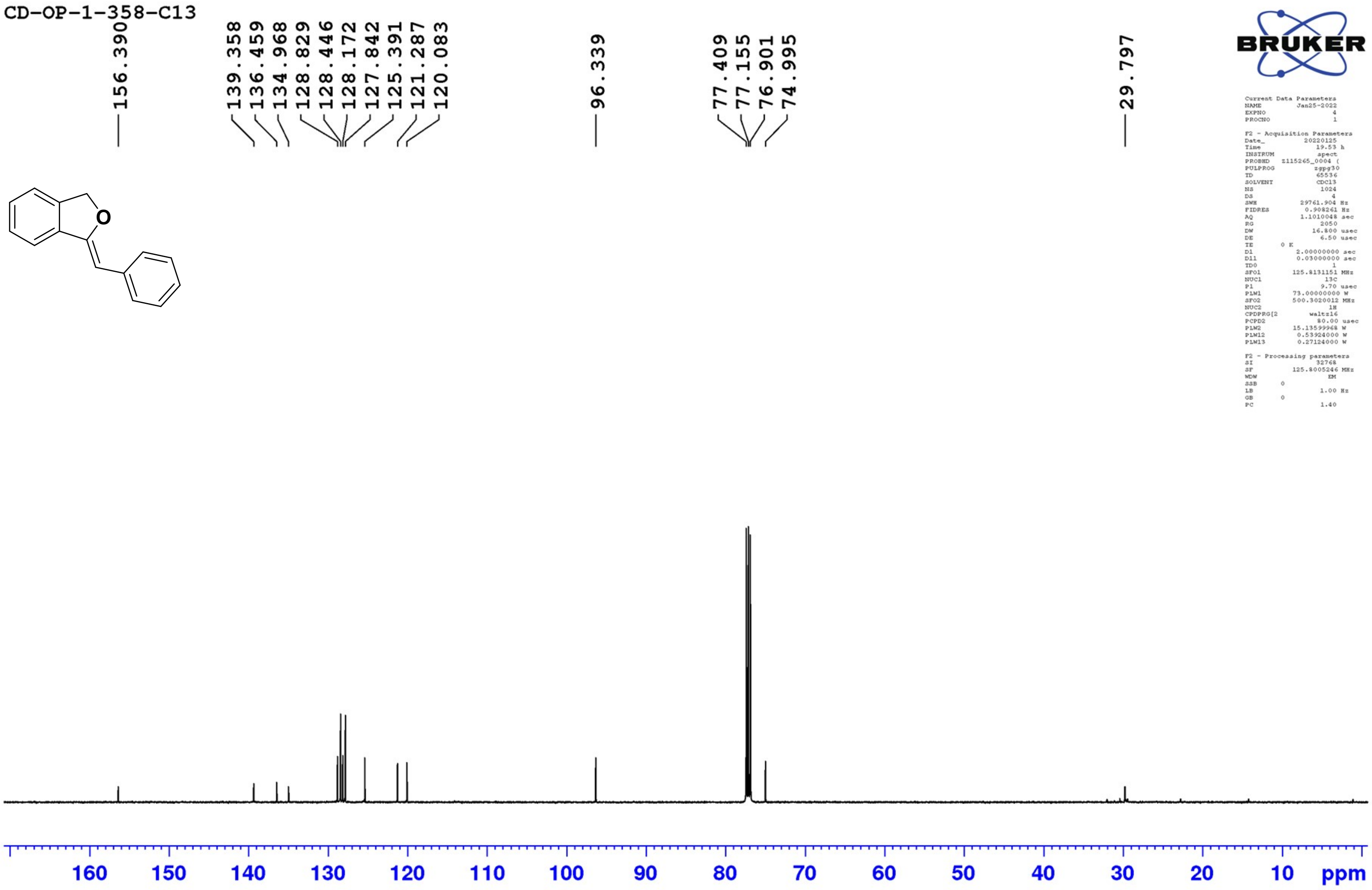

Figure S33. $^{13}C\{^{1}H\}$ NMR spectrum of **4aa** in $CDCl_3$

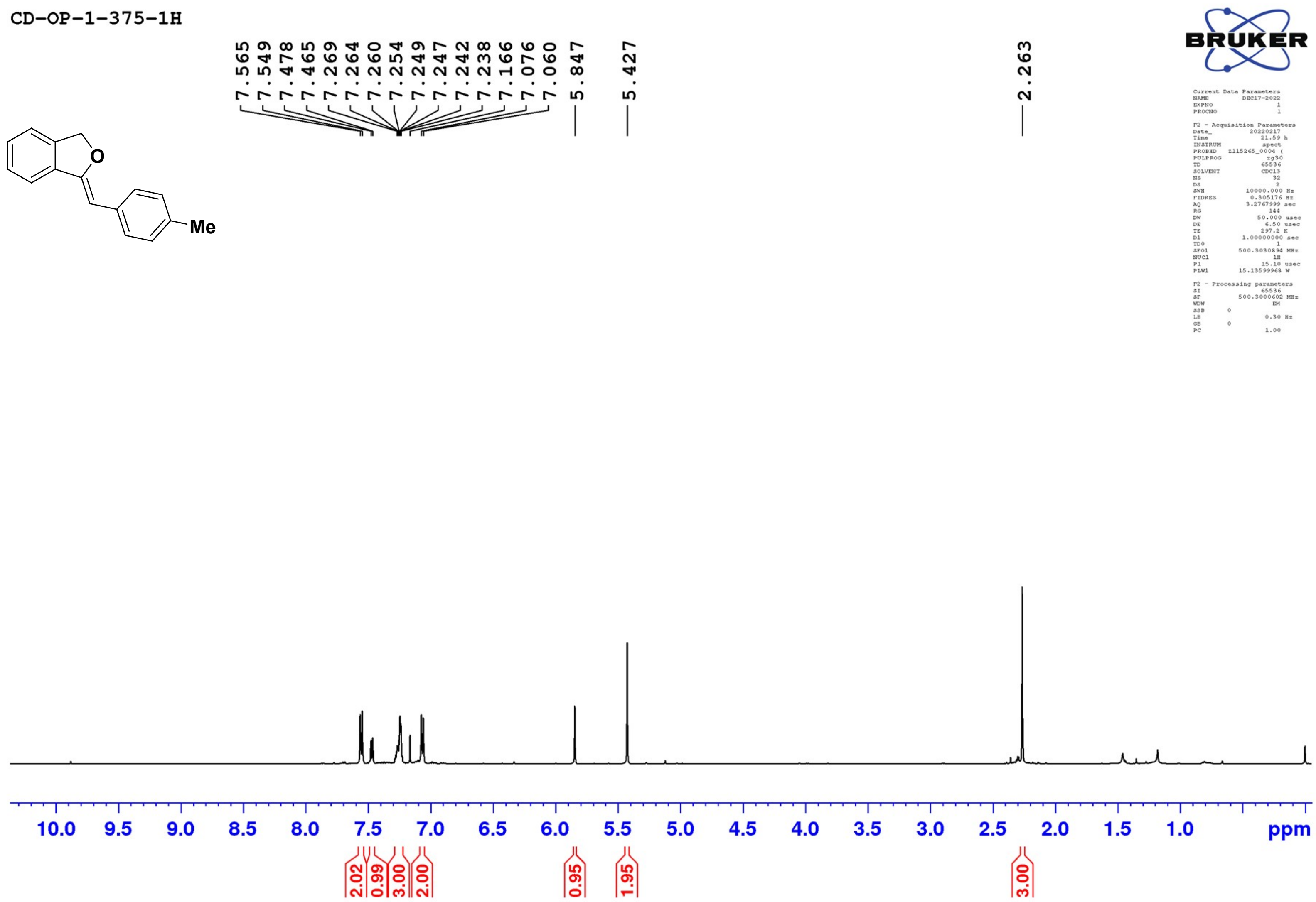

Figure S34. ^{1}H NMR spectrum of **4ab** in $CDCl_3$

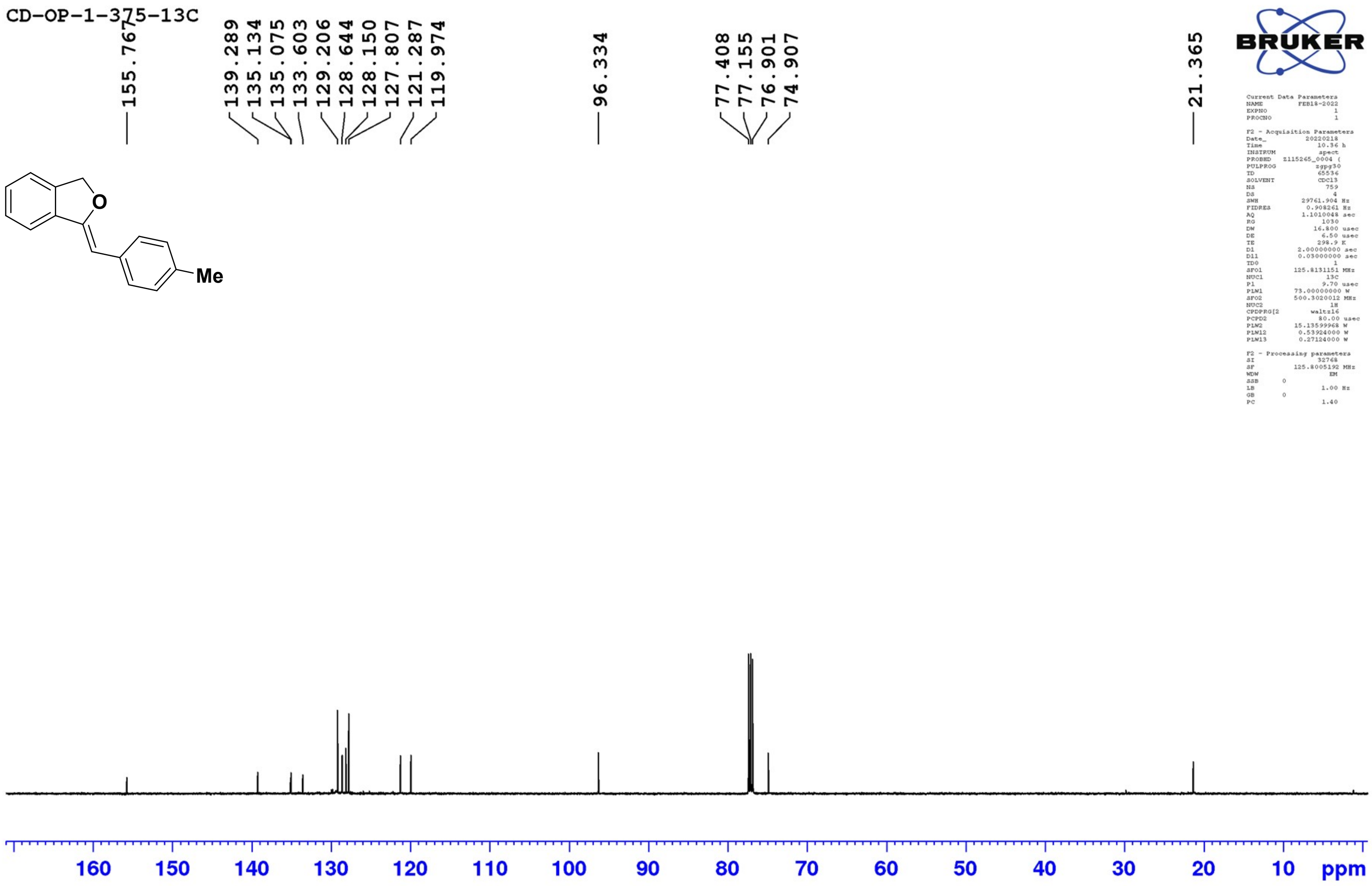

Figure S35. $^{13}C\{^{1}H\}$ NMR spectrum of **4ab** in $CDCl_3$

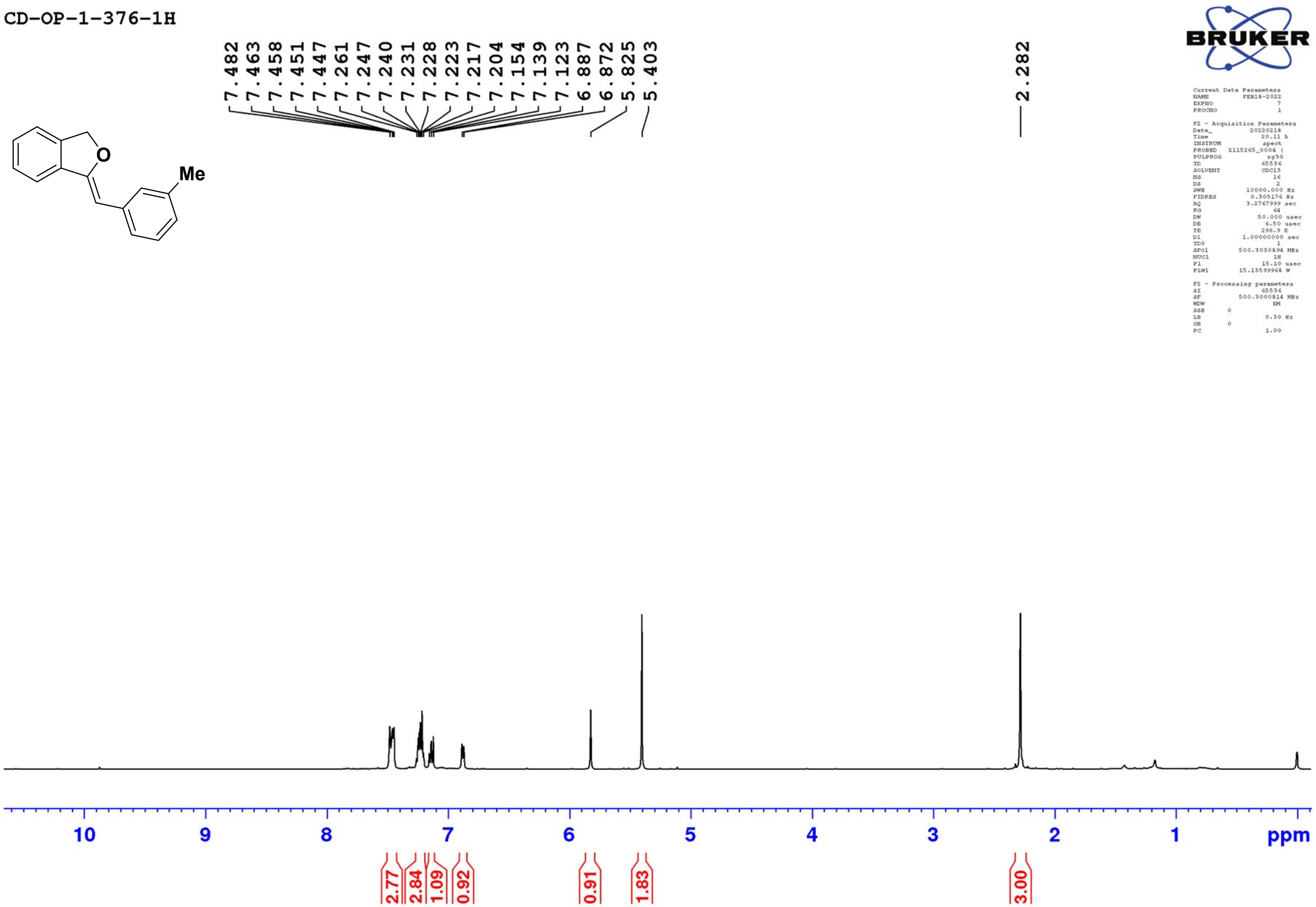

Figure S36. ^{1}H NMR spectrum of **4ac** in $CDCl_3$

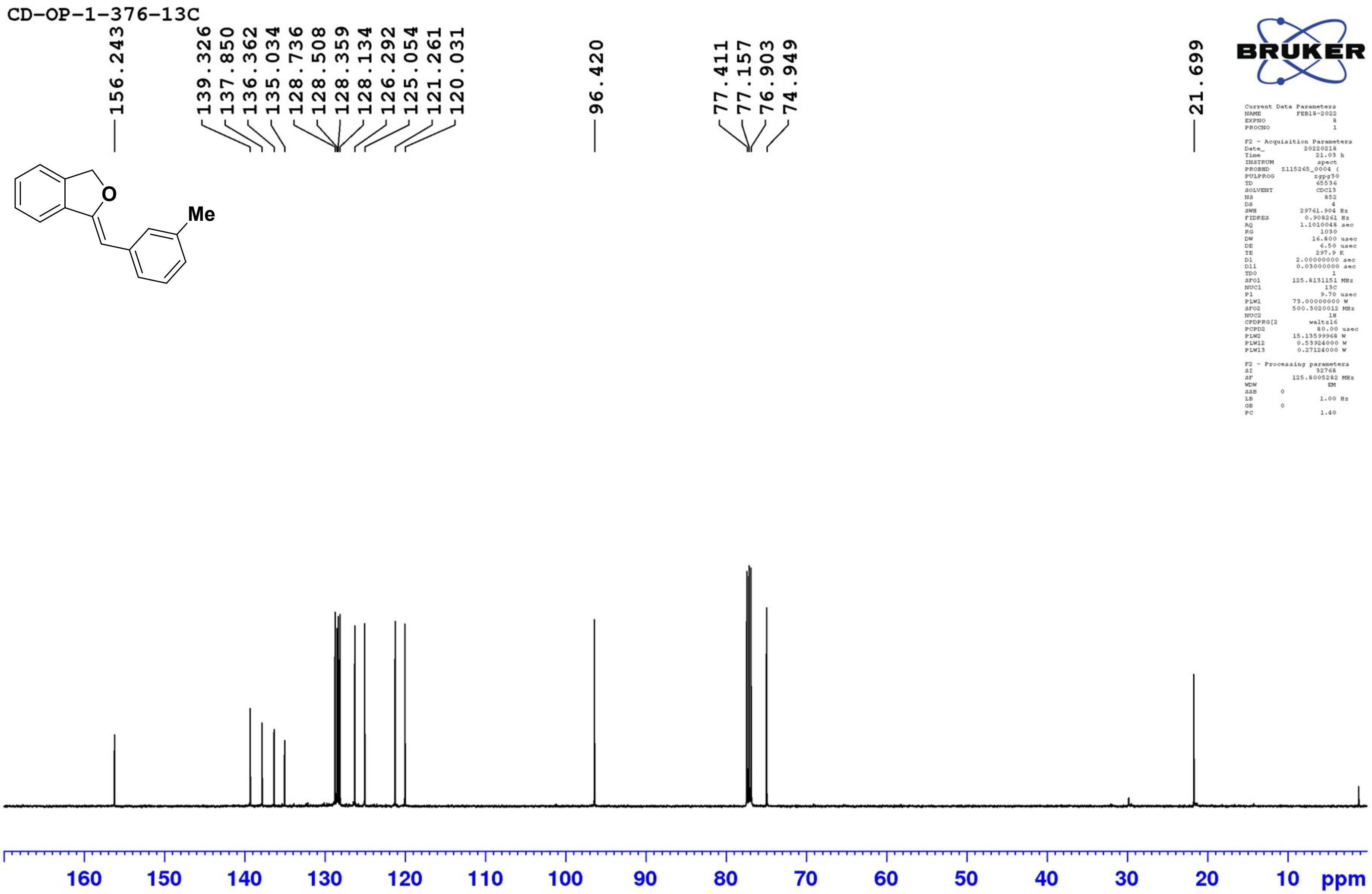

Figure S37. $^{13}C\{^{1}H\}$ NMR spectrum of **4ac** in $CDCl_3$

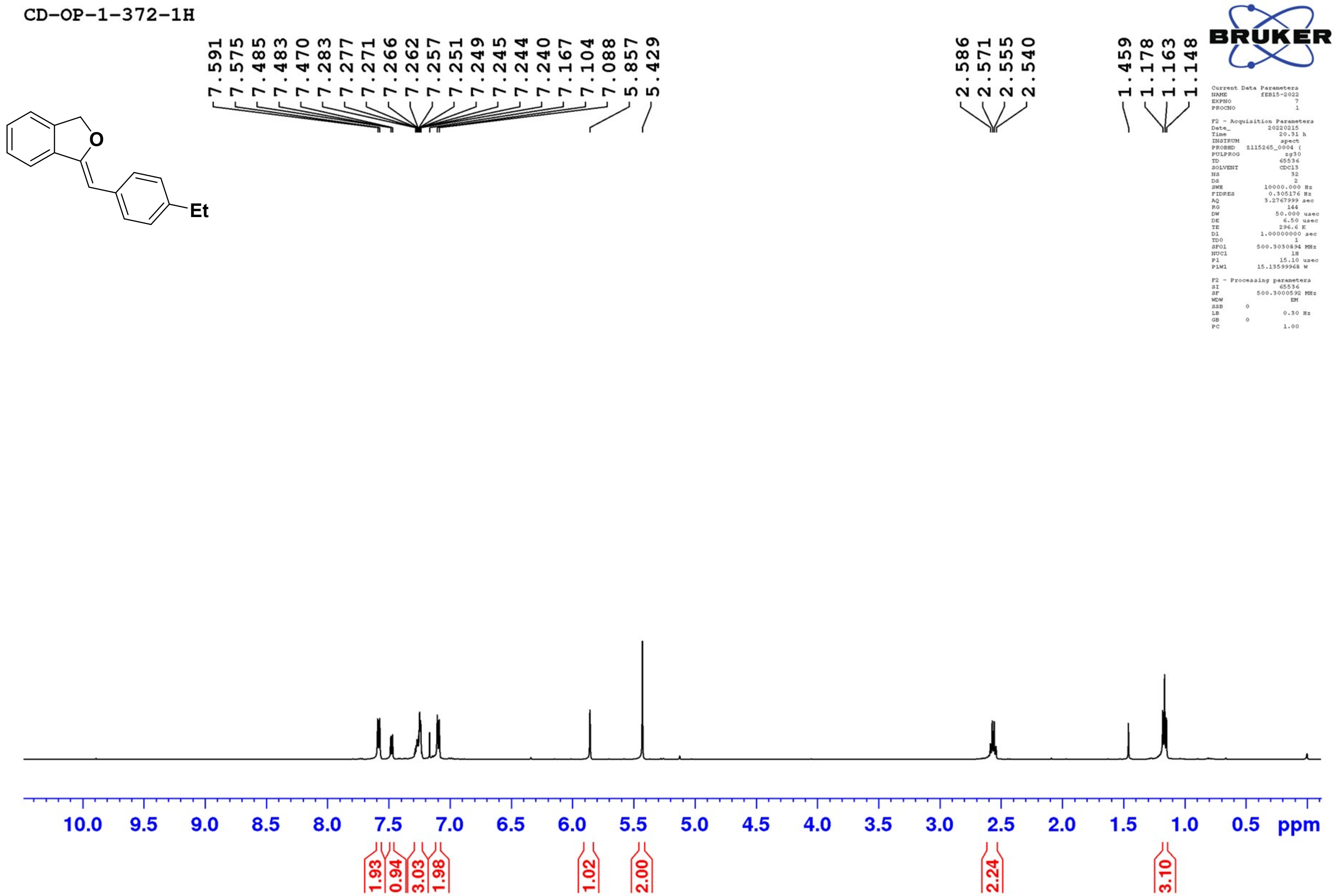

Figure S38. ^{1}H NMR spectrum of **4ad** in $CDCl_3$

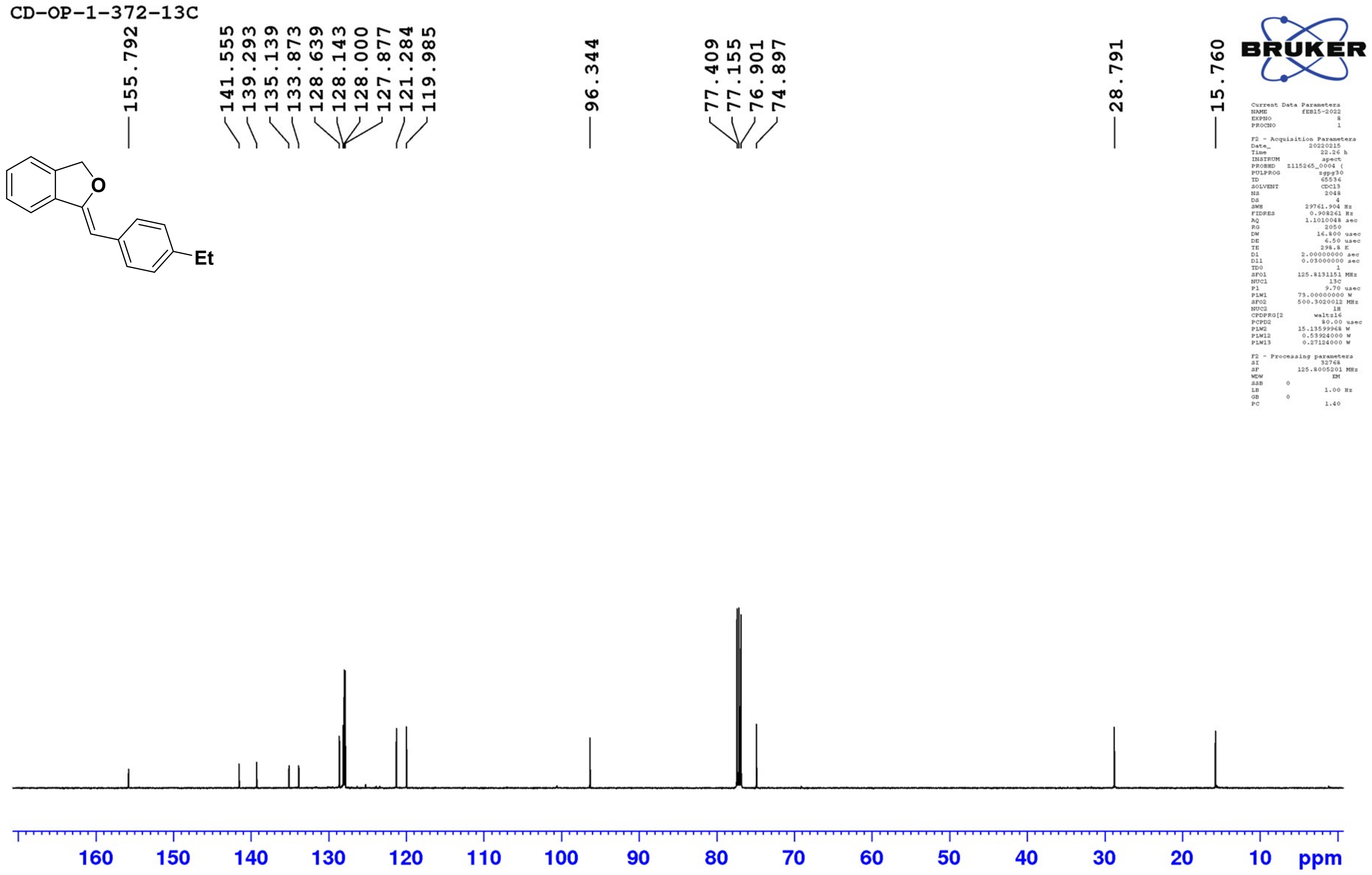

Figure S39. $^{13}C\{^1H\}$ NMR spectrum of **4ad** in $CDCl_3$

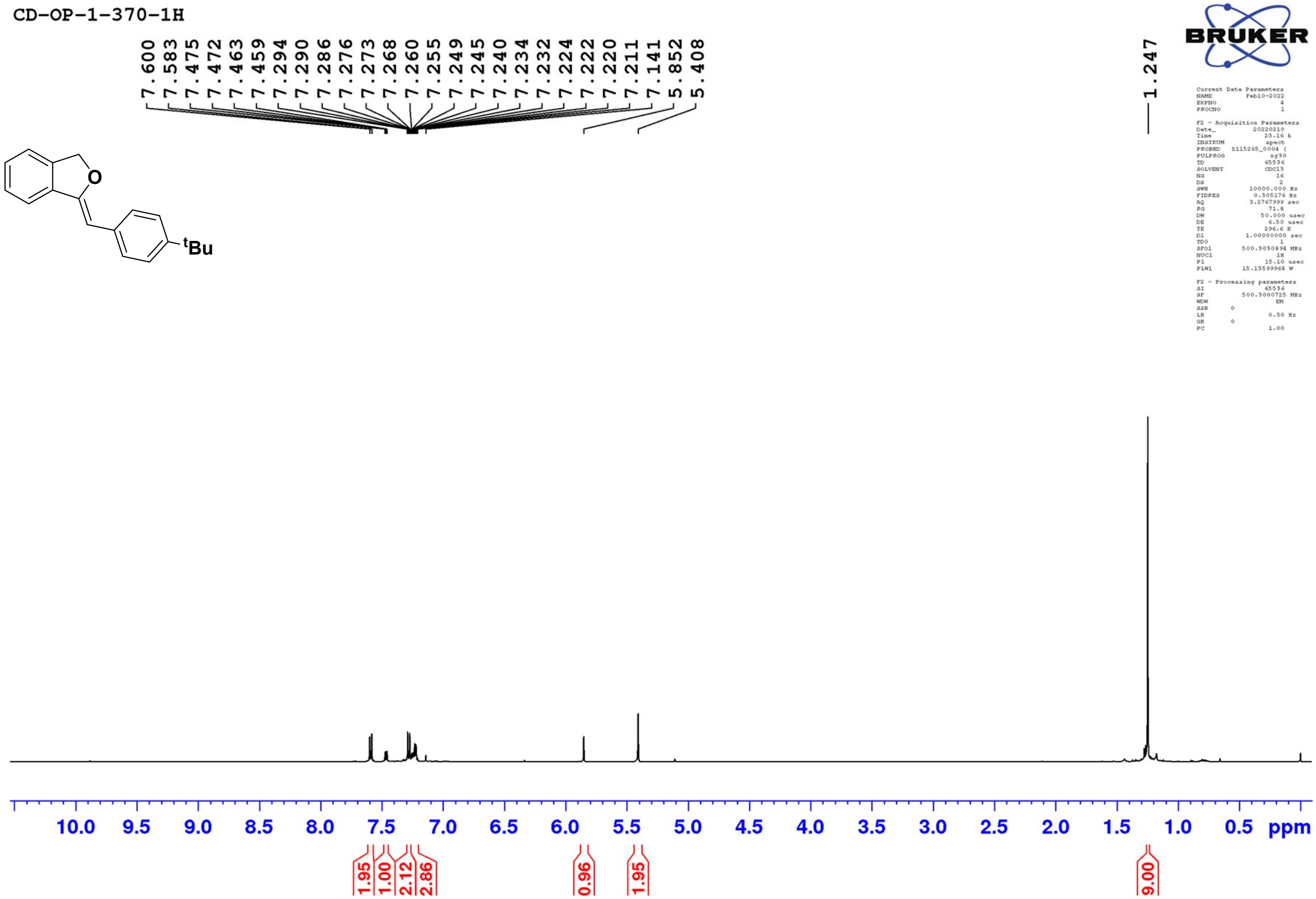

Figure S40. ^{1}H NMR spectrum of **4ae** in $CDCl_3$

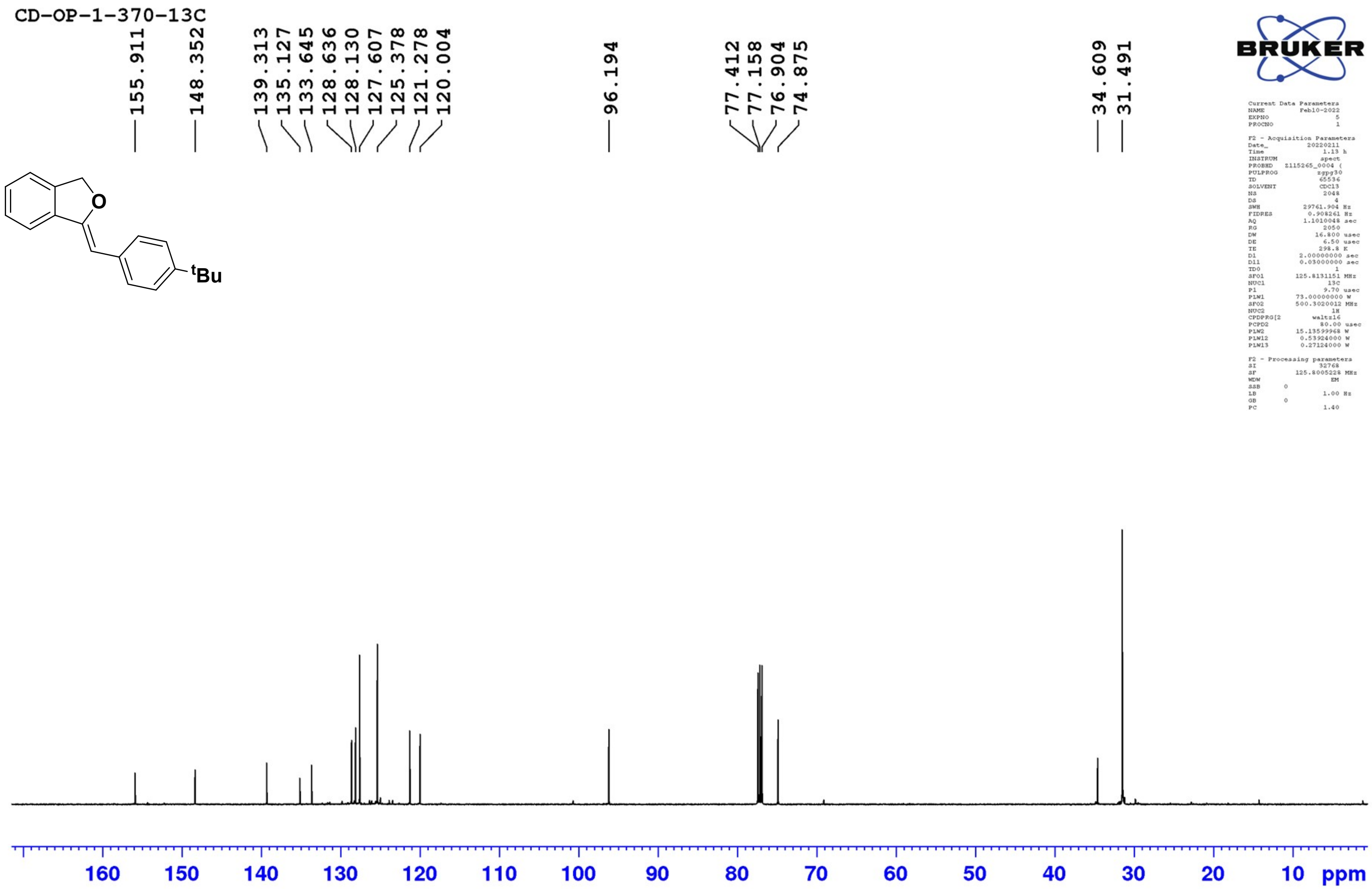

Figure S41. $^{13}C\{^1H\}$ NMR spectrum of **4ae** in $CDCl_3$

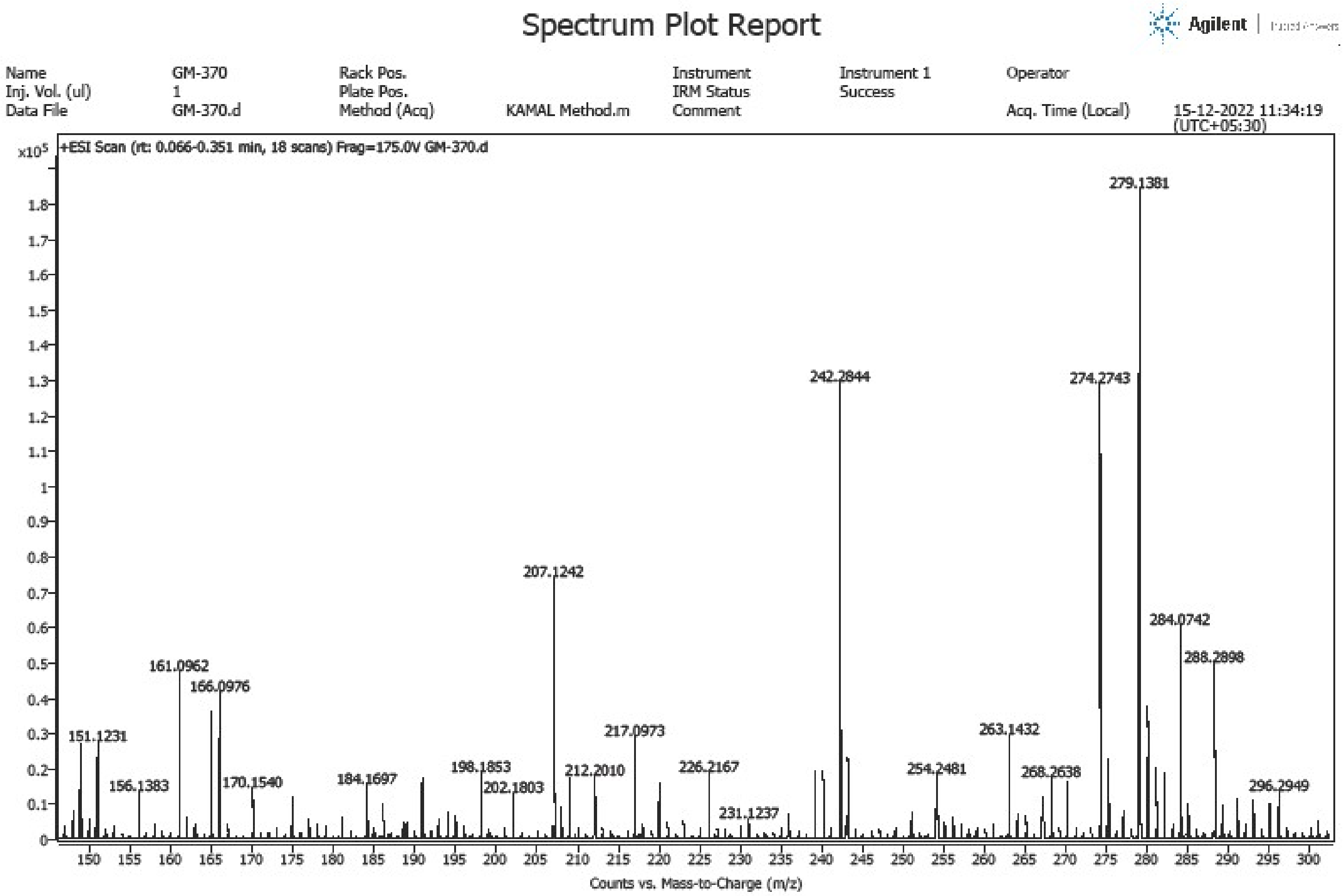

Figure S42. High Resolution Mass Spectrometry (HRMS) data of **4ae**

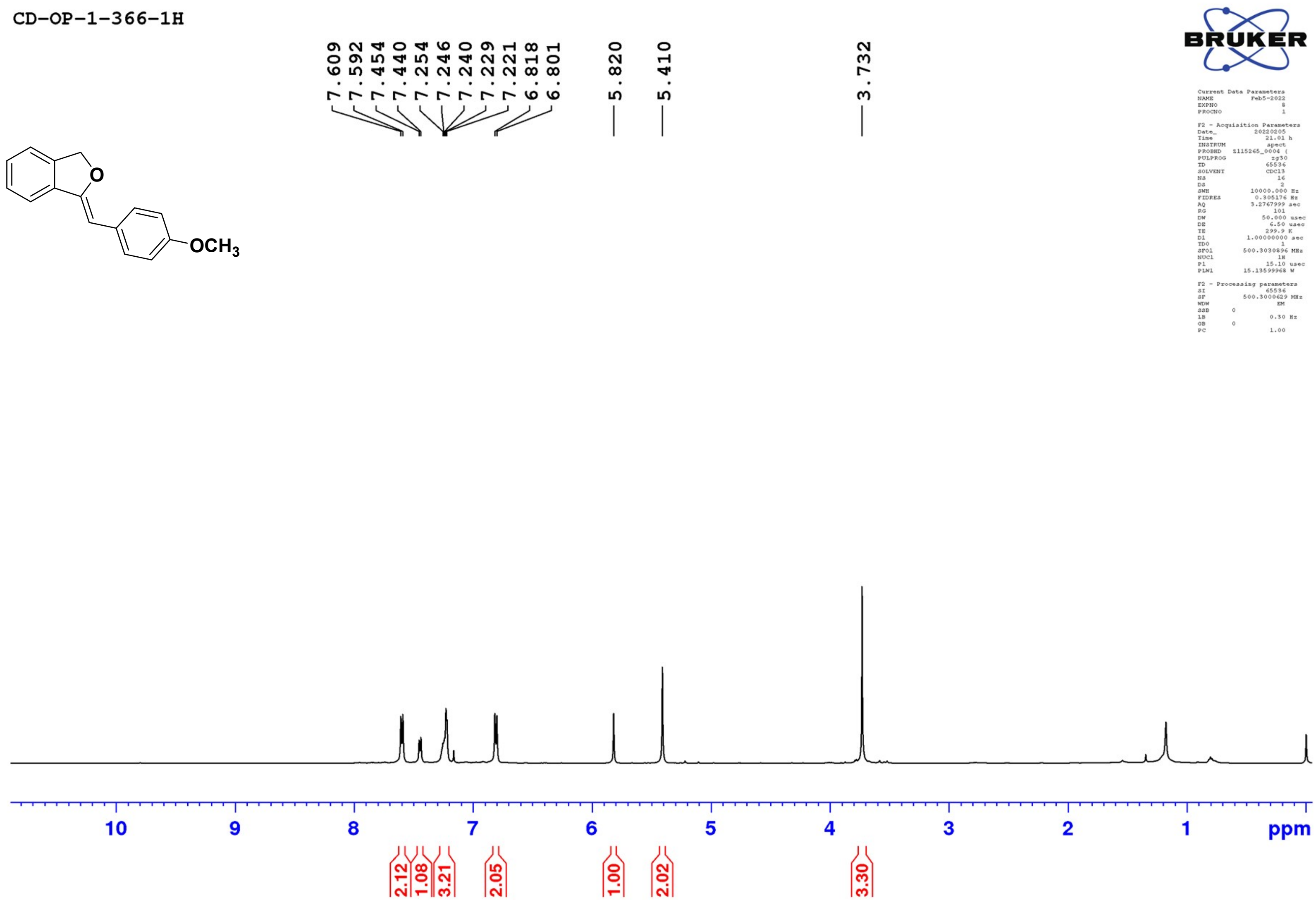

Figure S43. ^1H NMR spectrum of **4af** in $CDCl_3$

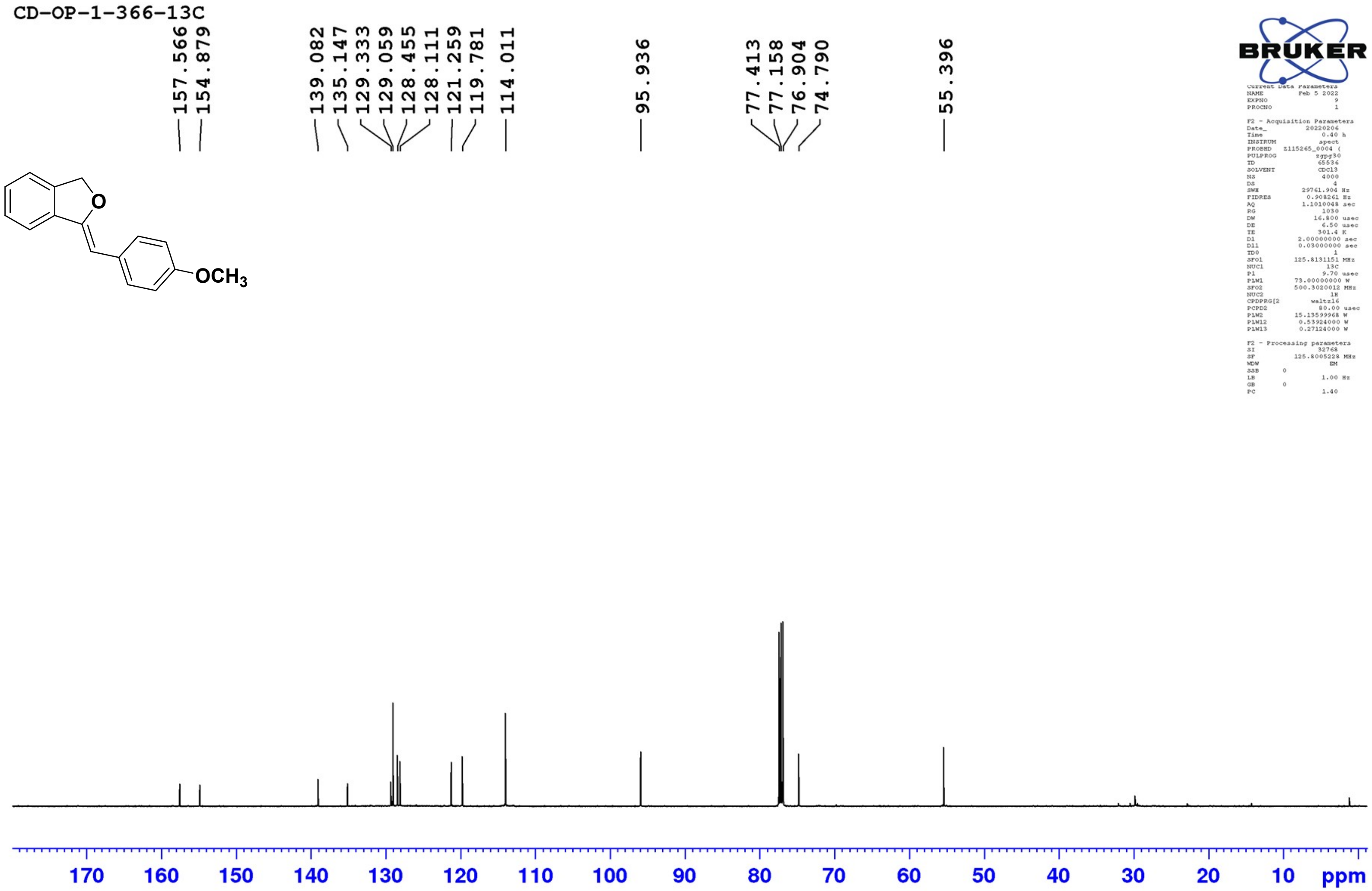

Figure S44. $^{13}C\{^{1}H\}$ NMR spectrum of **4af** in $CDCl_3$

Compound Spectra

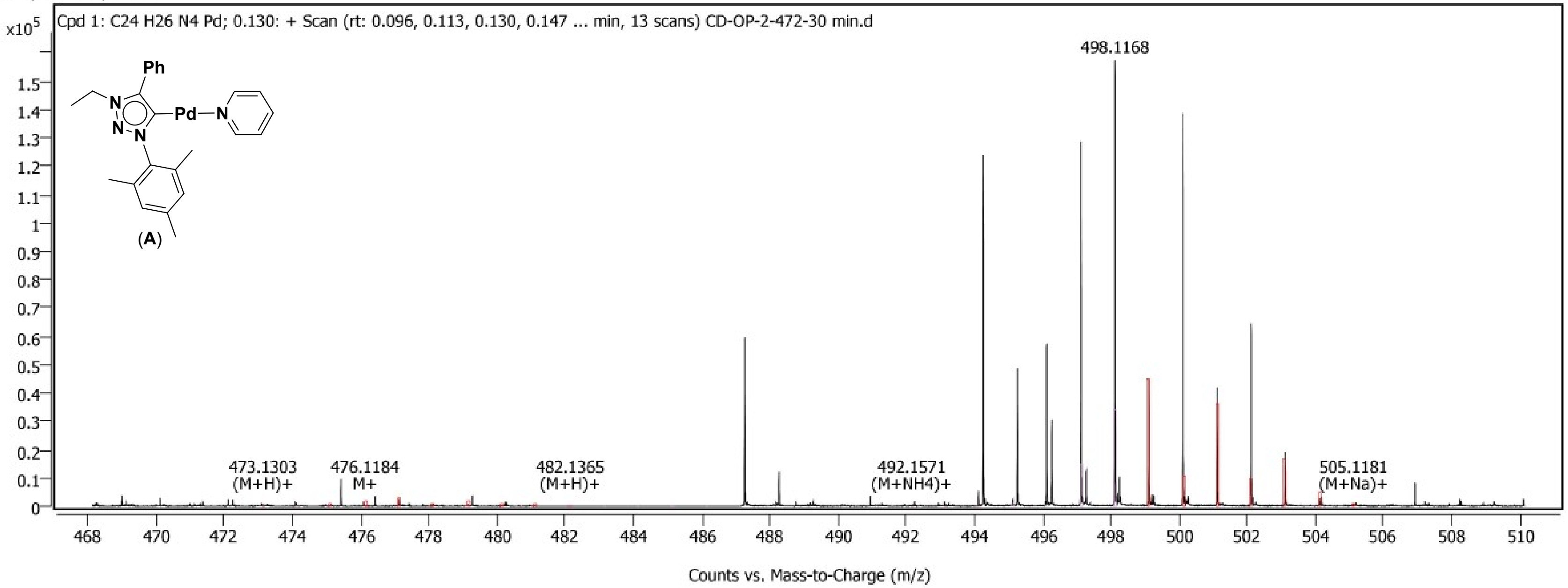

Spectrum Peaks

m/z	Z	Abund	Diff (ppm)	Height %	Height % (Calc)	Ion Species	Formula
473.1303	1	418	3.45	16.11	3.01	(M+H)+	C24H26N4Pd
475.1301	1	248	6.41	9.55	32.94	(M+H)+	C24H26N4Pd
476.1184	1	249	-2.45	100.00	100.00	M+	C24H26N4Pd
477.1257	1	2594	-3.73	100.00	100.00	(M+H)+	C24H26N4Pd
482.1365	1	157	10.77	6.06	9.82	(M+H)+	C24H26N4Pd
492.1571	1	167	6.94	48.66	32.84	(M+NH4)+	C24H26N4Pd
494.1466	1	343	-14.91	100.00	100.00	(M+NH4)+	C24H26N4Pd
499.1196	1	44955	20.50	100.00	100.00	(M+Na)+	C24H26N4Pd
505.1181	1	763	3.61	1.70	1.29	(M+Na)+	C24H26N4Pd
498.1168		158250					

Figure S45. High Resolution Mass Spectrometry (HRMS) data of Intermediate **A**

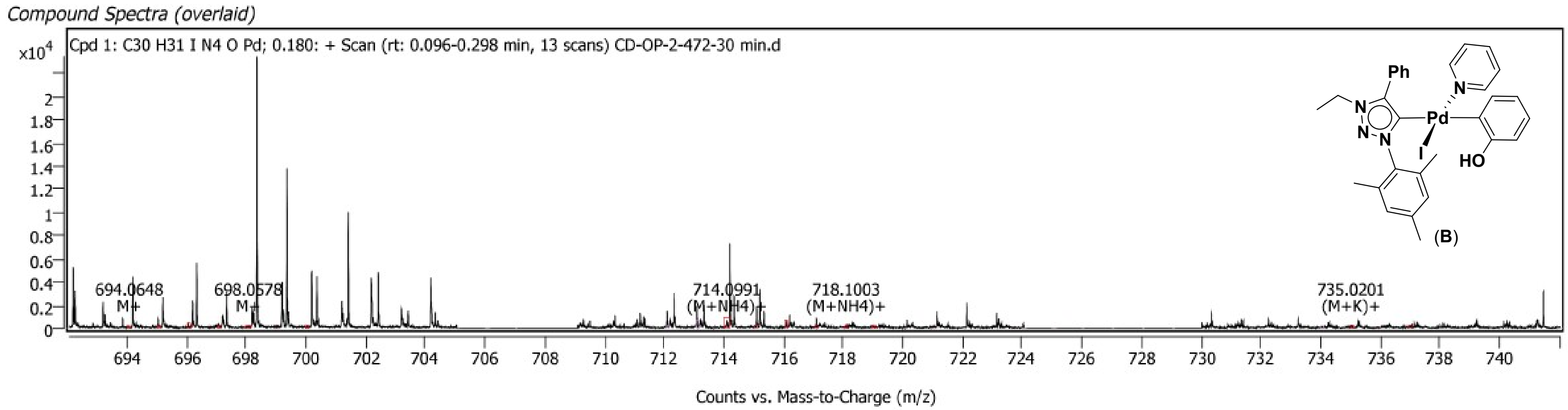

Spectrum Peaks

m/z	Z	Abund	Diff (ppm)	Height %	Height % (Calc)	Ion Species	Formula
694.0648	1	135	10.11	32.44	31.42	M+	C30H31IN4OPd
695.0669	1	331	11.21	79.74	73.38	M+	C30H31IN4OPd
696.0511	1	415	-10.38	100.00	100.00	M+	C30H31IN4OPd
698.0578	1	225	-0.22	54.34	79.20	M+	C30H31IN4OPd
699.0613	1	134	0.66	32.35	26.02	M+	C30H31IN4OPd
700.0534	1	157	-8.61	37.95	37.31	M+	C30H31IN4OPd
714.0991	1	718	9.01	98.60	100.00	(M+NH4)+	C30H31IN4OPd
716.0991	1	728	9.37	100.00	79.09	(M+NH4)+	C30H31IN4OPd
718.1003	1	366	8.99	50.22	37.31	(M+NH4)+	C30H31IN4OPd
719.0553	1	129	9.96	100.00	100.00	(M+Na)+	C30H31IN4OPd
719.1026	1	135	8.53	18.48	11.91	(M+NH4)+	C30H31IN4OPd
735.0201	1	266	-2.49	100.00	100.00	(M+K)+	C30H31IN4OPd
737.0221	1	162	0.71	60.64	84.50	(M+K)+	C30H31IN4OPd

Figure S46. High Resolution Mass Spectrometry (HRMS) data of Intermediate **B**

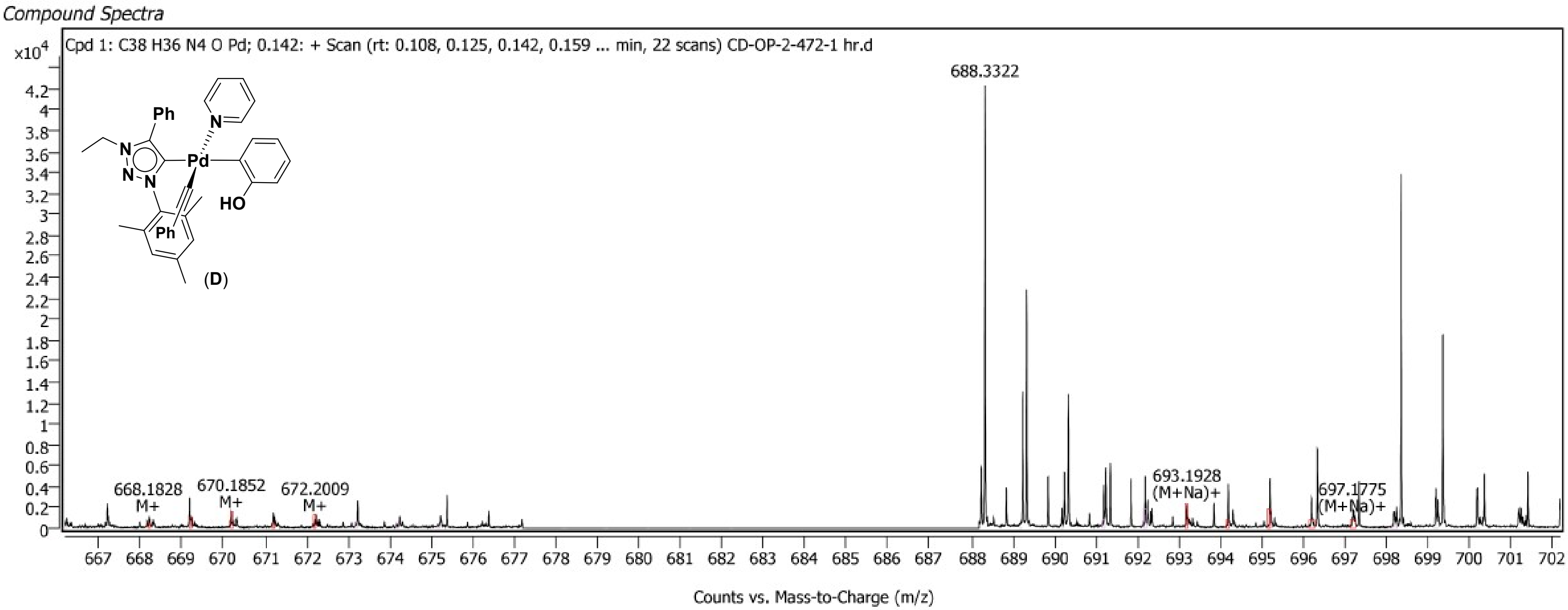

Spectrum Peaks

m/z	Z	Abund	Diff (ppm)	Height %	Height % (Calc)	Ion Species	Formula
668.1828	1	333	-14.49	21.57	29.59	M+	C38H36N4OPd
670.1852	1	1545	-11.94	100.00	100.00	M+	C38H36N4OPd
672.2009	1	855	11.91	55.31	77.16	M+	C38H36N4OPd
693.1928	1	2269	14.09	100.00	100.00	(M+Na)+	C38H36N4OPd
697.1775	1	1114	-9.54	49.11	37.41	(M+Na)+	C38H36N4OPd
688.3322		42712					

Figure S47. High Resolution Mass Spectrometry (HRMS) data of Intermediate **D**

Table S1. Crystal data and structure refinement for **2b**

Identification code	**2b**
Empirical formula	$C_{24}H_{26}N_4PdI_2$
Formula weight	730.69
Temperature/K	145(2)
Crystal system	monoclinic
Space group	$P2_1/n$
a/Å	9.5780(3)
b/Å	16.2412(6)
c/Å	33.3733(12)
α/°	90
β/°	96.3530(10)
γ/°	90
Volume/Å^3	5159.6(3)
Z	8
ρ_{calc}g/cm^3	1.881
μ/mm^{-1}	3.131
F(000)	2800.0
Crystal size/mm^3	0.2 × 0.17 × 0.14
Radiation	MoKα (λ = 0.71073)
2Θ range for data collection/°	4.456 to 51.448
Index ranges	$-11 \le h \le 10$, $-19 \le k \le 19$, $-40 \le l \le 40$
Reflections collected	52632
Independent reflections	9808 [R_{int} = 0.0509, R_{sigma} = 0.0365]
Data/restraints/parameters	9808/0/567
Goodness-of-fit on F^2	1.206
Final R indexes [I>=2σ (I)]	R_1 = 0.0430, wR_2 = 0.0883
Final R indexes [all data]	R_1 = 0.0498, wR_2 = 0.0906
Largest diff. peak/hole / e Å^{-3}	1.57/-0.87

Table S2. Bond lengths for **2b**

Atom	Atom	Length/Å	Atom	Atom	Length/Å
C1	C2	1.383(8)	C27	C28	1.383(8)
C1	N4	1.379(7)	C27	C32	1.397(8)
C1	Pd1	1.970(5)	C28	C29	1.391(9)
C2	C3	1.466(8)	C29	C30	1.374(9)
C2	N2	1.358(7)	C30	C31	1.382(9)
C3	C4	1.401(8)	C31	C32	1.379(9)
C3	C8	1.390(9)	C33	C34	1.497(10)
C4	C5	1.386(9)	C33	N6	1.478(8)
C5	C6	1.382(9)	C35	C36	1.398(9)
C6	C7	1.388(9)	C35	C40	1.384(9)
C7	C8	1.377(9)	C35	N8	1.451(8)

C9	C10	1.506(12)	C36	C37	1.387(10)
C9	N2	1.478(8)	C36	C41	1.516(10)
C11	C12	1.397(8)	C37	C38	1.410(11)
C11	C16	1.389(9)	C38	C39	1.378(10)
C11	N4	1.452(8)	C38	C42	1.523(10)
C12	C13	1.382(9)	C39	C40	1.387(9)
C12	C17	1.495(9)	C40	C43	1.510(9)
C13	C14	1.401(10)	C44	C45	1.390(9)
C14	C15	1.373(10)	C44	N5	1.321(8)
C14	C18	1.505(9)	C45	C46	1.361(10)
C15	C16	1.404(9)	C46	C47	1.382(10)
C16	C19	1.499(9)	C47	C48	1.376(9)
C20	C21	1.375(9)	C48	N5	1.347(8)
C20	N1	1.331(8)	N1	Pd1	2.116(5)
C21	C22	1.397(10)	N2	N3	1.322(7)
C22	C23	1.374(10)	N3	N4	1.328(7)
C23	C24	1.378(9)	N5	Pd2	2.107(5)
C24	N1	1.338(8)	N6	N7	1.319(7)
C25	C26	1.394(8)	N7	N8	1.331(7)
C25	N8	1.382(7)	Pd1	I1	2.6208(6)
C25	Pd2	1.980(6)	Pd1	I2	2.5973(6)
C26	C27	1.477(8)	Pd2	I3	2.5801(6)
C26	N6	1.359(7)	Pd2	I4	2.6281(6

Table S3. Bond angles for **2b**

**Atom**	**Atom**	**Atom**	**Angle/°**	**Atom**	**Atom**	**Atom**	**Angle/°**
C2	C1	Pd1	126.1(4)	C40	C35	C36	123.1(6)
N4	C1	C2	103.2(5)	C40	C35	N8	120.0(5)
N4	C1	Pd1	130.7(4)	C35	C36	C41	123.0(6)
C1	C2	C3	128.7(5)	C37	C36	C35	117.3(6)
N2	C2	C1	106.1(5)	C37	C36	C41	119.7(6)
N2	C2	C3	125.2(5)	C36	C37	C38	121.5(6)
C4	C3	C2	119.2(5)	C37	C38	C42	120.2(7)
C8	C3	C2	121.7(5)	C39	C38	C37	118.0(6)
C8	C3	C4	119.0(6)	C39	C38	C42	121.7(7)
C5	C4	C3	119.6(6)	C38	C39	C40	122.7(7)
C6	C5	C4	120.9(6)	C35	C40	C39	117.1(6)
C5	C6	C7	119.6(6)	C35	C40	C43	122.7(6)
C8	C7	C6	120.0(6)	C39	C40	C43	120.1(6)
C7	C8	C3	121.0(6)	N5	C44	C45	123.0(6)
N2	C9	C10	112.1(6)	C46	C45	C44	119.1(7)
C12	C11	N4	118.7(5)	C45	C46	C47	118.8(6)
C16	C11	C12	123.2(6)	C48	C47	C46	118.7(6)
C16	C11	N4	118.1(5)	N5	C48	C47	122.9(6)
C11	C12	C17	121.2(6)	C20	N1	C24	118.5(5)
C13	C12	C11	117.1(6)	C20	N1	Pd1	122.1(4)
C13	C12	C17	121.7(6)	C24	N1	Pd1	119.4(4)
C12	C13	C14	122.2(6)	C2	N2	C9	128.6(5)
C13	C14	C18	120.3(7)	N3	N2	C2	113.5(5)
C15	C14	C13	118.2(6)	N3	N2	C9	117.9(5)
C15	C14	C18	121.5(7)	N2	N3	N4	103.1(4)
C14	C15	C16	122.5(6)	C1	N4	C11	128.5(5)
C11	C16	C15	116.6(6)	N3	N4	C1	114.1(5)
C11	C16	C19	122.7(6)	N3	N4	C11	117.4(5)
C15	C16	C19	120.7(6)	C44	N5	C48	117.4(5)
N1	C20	C21	123.2(6)	C44	N5	Pd2	126.2(4)
C20	C21	C22	118.3(6)	C48	N5	Pd2	116.4(4)
C23	C22	C21	118.4(6)	C26	N6	C33	128.9(5)
C22	C23	C24	119.7(7)	N7	N6	C26	113.2(5)
N1	C24	C23	122.0(6)	N7	N6	C33	117.4(5)
C26	C25	Pd2	126.8(4)	N6	N7	N8	103.8(5)
N8	C25	C26	102.9(5)	C25	N8	C35	129.2(5)
N8	C25	Pd2	130.2(4)	N7	N8	C25	113.8(5)
C25	C26	C27	130.5(5)	N7	N8	C35	116.4(5)
N6	C26	C25	106.3(5)	C1	Pd1	N1	176.0(2)
N6	C26	C27	122.9(5)	C1	Pd1	I1	87.90(17)
C28	C27	C26	121.9(5)	C1	Pd1	I2	88.99(17)
C28	C27	C32	118.5(6)	N1	Pd1	I1	92.12(14)
C32	C27	C26	119.5(5)	N1	Pd1	I2	90.49(14)
C27	C28	C29	120.6(6)	I2	Pd1	I1	172.31(2)
C30	C29	C28	120.2(6)	C25	Pd2	N5	172.8(2)
C29	C30	C31	119.6(6)	C25	Pd2	I3	89.24(16)
C32	C31	C30	120.5(6)	C25	Pd2	I4	90.91(16)
C31	C32	C27	120.4(6)	N5	Pd2	I3	90.93(14)
N6	C33	C34	110.7(6)	N5	Pd2	I4	88.96(14)
C36	C35	N8	116.7(6)	I3	Pd2	I4	179.62(3)
